# Lactate‐Primed NETosis Modulates Hepatic Regeneration During Acute Liver Failure via the TLR9/KLF15/AJUBA Axis

**DOI:** 10.1111/cpr.70251

**Published:** 2026-06-22

**Authors:** Jin Guo, Xiaoya Zhang, Danmei Zhang, Chunxia Shi, Luwen Wang, Zuojiong Gong

**Affiliations:** ^1^ Department of Infectious Diseases Renmin Hospital of Wuhan University Wuhan China

**Keywords:** ALF, KLF15, lactate, liver regeneration, NETosis, TLR9

## Abstract

Acute liver failure (ALF) is characterised by massive hepatocyte death and compromised regenerative capacity, yet the metabolic‐immune crosstalk underlying these pathological processes remains poorly understood. Here, we demonstrate that lactate acts as a pivotal signal that triggers neutrophil extracellular traps (NETs) formation and release. Integrated RNA‐seq and scRNA‐seq analyses revealed profound glycolytic reprogramming in Kupffer cells (KCs) during ALF, leading to lactate accumulation within the hepatic microenvironment. Mechanistically, neutrophils import exogenous lactate into mitochondria via monocarboxylate transporter 1 (MCT1), which subsequently activates NETosis. Macrophage depletion or administration of an MCT1 inhibitor reduced NETs formation and ameliorated liver injury. Furthermore, we demonstrate that hepatocytes internalise NETs DNA, which is sensed by endosomal Toll‐like receptor 9 (TLR9). Activation of the TLR9 signalling pathway suppresses the expression of Krüppel‐like factor 15 (KLF15). This downregulation diminishes AJUBA and disrupts the KLF15‐AJUBA interaction, thereby increasing the phosphorylation of YAP1 and impeding hepatocyte proliferation. Notably, KLF15 overexpression bypassed TLR9‐mediated inhibitory signals and rescued the NETs‐induced regenerative failure in vitro. In conclusion, our study elucidates a novel KCs‐neutrophil‐hepatocyte crosstalk wherein lactate‐driven NETosis thwarts liver regeneration via the TLR9/KLF15/AJUBA axis, thereby identifying potential therapeutic targets for the clinical management of ALF.

## Graphical abstract

This study elucidates a novel Kupffer cell‐neutrophil‐hepatocyte axis that drives metabolic‐immune crosstalk during acute liver failure. Specifically, lactate‐primed NETs release links metabolic reprogramming to NETosis‐induced regenerative failure in hepatocytes via the TLR9/KLF15/AJUBA axis.

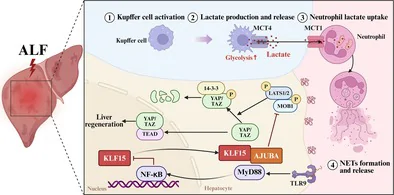

## 1 Introduction

Acute liver failure (ALF) is a severe clinical syndrome characterised by hepatic dysfunction, coagulopathy, jaundice, and encephalopathy in the absence of preexisting liver disease, frequently accompanied by rapid clinical deterioration [1]. Given the rapid progression of ALF, early identification and intervention are paramount for improving therapeutic efficacy and prognosis. Irrespective of the aetiology, ALF typically shares a conserved pathological trajectory, initially manifesting as excessive immune activation, cytokine storms, and cascading immune cell recruitment [2], which subsequently transitions into immune paralysis characterised by impaired phagocytosis and opsonization [3]. Kupffer cells (KCs), the predominant liver‐resident macrophages localized primarily within the hepatic sinusoids, play an initiating role in ALF. KCs recognize damage‐associated molecular patterns (DAMPs) released from necrotic hepatocytes, thereby secreting various pro‐inflammatory cytokines and chemokines to trigger the recruitment and activation of diverse immune cells [4]. Importantly, metabolic reprogramming is a critical hallmark of KCs' activation. Activated KCs have been reported to drive glycolysis, generating and secreting massive amounts of lactate via the dimerization of pyruvate kinase M2 (PKM2) [5] and the upregulation of lactate dehydrogenase A (LDHA) [6]. Lactate, once considered merely a metabolic byproduct, is now recognised to regulate gene transcription through post‐translational modifications (PTMs). It plays a pivotal role in modulating calcium signalling, cellular energy metabolism, and the functional activity of various ion channels and transporters [7, 8]. Furthermore, a recent study on acetaminophen (APAP)‐induced acute liver injury reported that lactate accumulation elevates mitochondrial protein lactylation levels, thereby exacerbating hepatic damage [9]. During ALF, the capacity of hepatocytes to clear lactate is markedly impaired, which subsequently reshapes the hepatic inflammatory and metabolic microenvironment, leading to the extensive recruitment of neutrophils, natural killer (NK) cells, and monocyte‐derived macrophages [10]. While the role of the inflammatory mediators (e.g., CXCL1, CXCL2, and IL‐1β) in neutrophil recruitment and activation is well‐established [11, 12, 13], whether alterations in the metabolic milieu also contribute to neutrophil infiltration and activation remains largely elusive.

Neutrophils are the most abundant innate immune cells. Upon activation, they eliminate pathogens, dead cells, and cellular debris via phagocytosis, a process intimately coupled with the generation of oxidative stress, degranulation, and the release of neutrophil extracellular traps (NETs) [14]. NETs are web‐like extracellular structures composed of a DNA backbone decorated with proteins such as myeloperoxidase (MPO), neutrophil elastase (NE), and histones, primarily serving to capture and confine invading pathogens [15]. Our previous studies demonstrated that enhanced neutrophil recruitment and excessive NETosis occur during ALF, both of which are strongly correlated with the severity of liver injury [16]. Throughout the onset and progression of ALF, NETs exert dual pathogenic effects: they induce direct bystander tissue damage via localized protease activity and subsequently amplify pro‐inflammatory signalling cascades within neighbouring immune cells [17]. While elevated NETs levels are consistently associated with hepatic injury in both animal models [18] and clinical samples [19], the specific components of this macromolecular DNA‐protein complex responsible for such damage remain poorly defined. Previous studies have reported that macrophage engulfment of NETs leads to the binding of double‐stranded DNA (dsDNA) to absent in melanoma 2 (AIM2), which triggers macrophage pyroptosis and thus accelerates the progression of hepatic inflammation and fibrosis [20]. Furthermore, compelling evidence suggests that NE facilitates insulin resistance and hepatic lipid accumulation via the AMP‐activated protein kinase (AMPK) signalling pathway, concurrently inducing the release of pro‐inflammatory cytokines [21, 22]. However, it remains incompletely understood whether NETs directly target hepatic parenchymal cells and, if so, the precise underlying molecular mechanisms. Therefore, identifying the core pathogenic components of NETs that directly target hepatocytes is of paramount importance for disrupting the cascade of NETs‐induced liver injury.

Although the liver possesses a robust regenerative capacity essential for hepatocyte repair, recovery from injury, and the resolution of inflammation [23, 24]. During acute liver injury (ALI), low‐level inflammatory signalling promotes hepatic repair. However, when liver injury progresses to ALF, it is characterised by hepatocyte cell cycle arrest or aberrant proliferation [24, 25]. The classical liver regeneration‐associated pathways include the Hippo and WNT signalling pathways. Under physiological homeostasis, the Hippo pathway is constitutively active, restricting excessive hepatocyte proliferation. Conversely, upon liver injury, the core kinases of the Hippo pathway are inhibited, allowing the key effectors YAP and TAZ to undergo dephosphorylation and nuclear translocation. This initiates the transcription of cell cycle proteins such as Cyclin D1 and c‐Myc to drive liver regeneration [26]. In the WNT signalling pathway, the activation of Dishevelled leads to the disassembly of the destruction complex, allowing β‐catenin to be dephosphorylated and translocate into the nucleus, where it initiates the transcription of target molecules such as axis inhibition protein 2 (Axin2) [27]. Recent studies have reported that modulating the cell cycle using tetrahedral framework nucleic acids [28] or activating the WNT signalling pathway via gallium‐doped MXene nanozymes [29] promotes hepatocyte proliferation and liver regeneration, thereby ameliorating liver injury. Krüppel‐like factors (KLFs) are a subfamily of zinc‐finger transcription factors, among which KLF15 is highly expressed in the liver. Previous studies have highlighted the critical regulatory role of KLF15 in metabolism, including gluconeogenesis [30], bile acid metabolism [31], corticosteroid synthesis [32], and branched‐chain amino acid (BCAA) metabolism [33]. Furthermore, it has been suggested that the overexpression of Klf15 ameliorates hepatic injury in mouse models of alcoholic liver disease [34]. However, direct evidence regarding the role of KLF15 in orchestrating liver regeneration and its association with NETs remains lacking.

In the present study, we aimed to elucidate the cross‐talk between metabolic reprogramming and immune activation during ALF. We discovered that activated KCs generate and release substantial amounts of lactate, which is subsequently internalised by neutrophils to trigger extensive NETosis. These KCs‐driven NETs then directly target hepatocytes, where NETs DNA is recognised by endosomal Toll‐like receptor 9 (TLR9), activating the NFκB p65 signalling cascade. Crucially, this inflammatory signalling leads to the downregulation of KLF15 in hepatocytes, which further suppresses the expression of its downstream target, AJUBA. The loss of AJUBA activates the Hippo pathway, consequently suppressing YAP1 activity and ultimately impeding the initiation of liver regeneration. Taken together, this study demonstrates that KCs‐derived, lactate‐mediated NETs formation serves as a critical negative regulator of hepatic regeneration. The KLF15/AJUBA/YAP1 signalling axis thus emerges as a promising therapeutic target for promoting hepatic repair and improving the clinical prognosis of patients with ALF.

## 2 Materials and Methods

### 2.1 Cell Culture, Induction, and Treatment

Human promyelocytic leukaemia HL‐60 cells (Wuhan University Cell Collection Center, China) were cultured in Iscove's Modified Dulbecco's Medium (IMDM; Gibco, USA) supplemented with 20% fetal bovine serum (FBS; Solarbio, China) and 1% penicillin–streptomycin‐gentamicin (PSG; Biosharp, China) at 37°C in a humidified atmosphere containing 5% CO_2_. Neutrophil‐like differentiation was induced by treating the cells with 1 μM all‐trans retinoic acid (ATRA; Solarbio, China) for 5 days. For lactate treatment, the pH of the lactate solution was adjusted to 7.4 using NaOH (Biosharp, China), and lactate was subsequently added according to the indicated time points and concentrations. For the monocarboxylate transporter 1 (MCT1) inhibitor group, cells were pre‐treated with 100 nM AZD3965 (MedChemExpress, USA) for 2 h prior to lactate exposure. For the phorbol 12‐myristate 13‐acetate (PMA) group, cells were treated with 100 nM PMA (Sigma‐Aldrich, USA) for 4 h. For the lipopolysaccharide (LPS) group, neutrophil‐like differentiated HL‐60 (dHL‐60) cells were treated with 1 μg/mL LPS (Sigma‐Aldrich, USA) for 24 h.

The murine macrophage cell line RAW264.7 and the human monocyte cell line THP‐1 (both from Servicebio, China) were cultured in Dulbecco's Modified Eagle Medium (DMEM; Servicebio, China) and Roswell Park Memorial Institute 1640 medium (RPMI 1640; Servicebio, China), respectively. Both media were supplemented with 10% FBS and 1% PSG, and cells were maintained at 37°C with 5% CO_2_. Macrophage‐like differentiation of THP‐1 cells was induced by treatment with 1 nM PMA for 48 h. For the LPS group, RAW264.7 and differentiated THP‐1 cells were treated with 1 μg/mL LPS for 24 h.

Human hepatoblastoma HepG2 cells and murine hepatocyte AML12 cells (both from Servicebio, China) were cultured in DMEM and DMEM/F‐12 (Servicebio, China), respectively, supplemented with 10% FBS and 1% PSG at 37°C in 5% CO_2_. The LPS/D‐galactosamine (D‐Gal) group was treated with 1 μg/mL LPS and 44 μg/mL D‐Gal (Sigma‐Aldrich, USA) for 6 or 24 h. For NETs treatment, cells were incubated with 10 or 30 μg/mL NETs for 6 or 24 h. For deoxyribonuclease I (DNase I) treatment, 15 IU/mL DNase I (Yeasen, China) was added 15 min prior to NETs stimulation.

### 2.2 Lactate Uptake Assay

Confocal dishes were pre‐coated with poly‐L‐lysine (PLL). dHL‐60 cells or mouse bone marrow‐derived neutrophils were seeded into the dishes and incubated with Cy3‐labelled lactate (Stargraydye, China), which was pre‐neutralised with NaOH. The cell membranes were subsequently stained with PKH67 to monitor the intracellular transport and dynamics of lactate at 2, 4, 8, 12, and 24 h post‐treatment. Furthermore, to elucidate the precise subcellular localization of the internalised lactate, the mitochondria of dHL‐60 cells and mouse bone marrow‐derived neutrophils were stained with MitoTracker Deep Red. The spatial co‐localization between the Cy3‐lactate fluorescence and the mitochondria was then visualised and analysed using a confocal microscope (Olympus, Japan).

### 2.3 Animals

Male C57BL/6J mice (6–8 weeks old, 20–25 g) were purchased from Vital River Laboratory (Beijing, China). The mice were housed in a specific pathogen‐free environment maintained at 22°C with 40%–70% relative humidity, under a standard 12‐h light/dark cycle. All animal procedures were approved by the Institutional Animal Care and Use Committee of Renmin Hospital of Wuhan University (approval no. WDRM [Welfare] 20240309F).

### 2.4 Isolation of Mouse Bone Marrow‐Derived Neutrophils

The tibiae and femora of the mice were carefully isolated, and the surrounding muscle tissues were removed to expose the bone marrow cavities. Bone marrow cells were collected by flushing the cavities with sterile PBS using a syringe. The cell suspension was then passed through a 70‐μm cell strainer, followed by erythrocyte depletion using a red blood cell lysis buffer. For density gradient centrifugation, Histopaque 1119 and 1077 (Sigma‐Aldrich, USA) were carefully layered in a 15 mL centrifuge tube, and an equal volume of the cell suspension was gently loaded onto the top layer. After centrifugation, the neutrophil‐rich fraction was collected, washed, and cultured in RPMI 1640 medium supplemented with 10% FBS and 1% PSG. Neutrophil purity (> 85%) was verified by flow cytometry using APC‐conjugated anti‐Ly6G and PE‐conjugated anti‐CD11b antibodies (Biolegend, USA).

### 2.5 Western Blotting (WB) and Co‐Immunoprecipitation (Co‐IP)

Cultured cells and mouse liver tissues were subjected to ultrasonic lysis and subsequently centrifuged at 12,000 rpm for 15 min at 4°C to collect the protein lysates. For Co‐IP samples, FLAG and HA immunoprecipitation kits (Beyotime, China) were utilised to capture FLAG‐ and HA‐tagged proteins, respectively. Protein concentrations were determined using a BCA protein assay kit (Yeasen, China). Following denaturation at 95°C for 10 min, equal amounts of protein (30 μg per lane) were separated via sodium dodecyl sulfate‐polyacrylamide gel electrophoresis (SDS‐PAGE) and transferred onto membranes. The membranes were blocked and subsequently incubated with specific primary antibodies. Detection was performed using horseradish peroxidase (HRP)‐conjugated secondary antibodies and visualised with a ChemiDoc imaging system (Bio‐Rad, USA). Detailed information regarding all primary antibodies used in this study is provided in Table S1. Original WB images are provided in the Supporting Information: ‘Supplementary Origin Western Blots’ file.

### 2.6 Quantitative Real‐Time PCR (qRT‐PCR)

Total RNA from mouse liver tissues and cultured cells was extracted using the TRIzol‐chloroform‐isopropanol method. The RNA pellets were washed with ethanol, air‐dried, and dissolved in diethyl pyrocarbonate (DEPC)‐treated water. Complementary DNA (cDNA) was synthesised using a reverse transcription kit (Servicebio, China). For quantitative analysis, the cDNA was mixed with specific primers and SYBR Green master mix in a 96‐well plate, and amplification was performed using the LightCycler 480 system (Roche, USA). Relative gene expression was calculated using the 2^−ΔΔ*Ct*^ method, with *ACTB* serving as the endogenous reference gene. The sequences of all primers used in this study are detailed in Table S2.

### 2.7 Assay Kits

Serum levels of alanine aminotransferase (ALT) and aspartate aminotransferase (AST) were measured using commercial assay kits (Nanjing Jiancheng, China) according to the manufacturer's protocols. Serum levels of cell‐free DNA (cfDNA), L‐lactate, and lactate dehydrogenase (LDH) were quantified using specific detection kits (Invitrogen, USA; Nanjing Jiancheng, China). Additionally, cfDNA levels in the culture supernatants of dHL‐60 cells and mouse bone marrow‐derived neutrophils were measured using the aforementioned cfDNA assay kit. All optical density or fluorescence results were recorded using a microplate reader (PerkinElmer, USA).

### 2.8 Bioinformatics Analysis

For bulk RNA sequencing (RNA‐seq) analysis, the ALF mouse dataset (GSE217659 [35]), partial hepatectomy (PHx) mouse dataset (GSE158864 [36]), APAP‐related ALF patient dataset (GSE120652), HBV‐related ALF patient dataset (GSE38941 [37]), and HBV‐related ACLF patient dataset (GSE222856 [38]) were retrieved from the Gene Expression Omnibus (GEO) database. Differential gene expression analysis was conducted using the DESeq2 package in R (version 4.3.1), applying cutoff thresholds of |log_2_FC| > 1 and *p* < 0.05. Volcano plots and heatmaps were generated using the ggplot2 package. Gene Ontology (GO) enrichment analysis was subsequently performed to identify overrepresented biological processes.

For single‐cell RNA sequencing (scRNA‐seq) analysis, data from ALF (GSE254497 [39]) and PHx (GSE158866 [36]) mouse liver tissue were retrieved from the GEO database. Data preprocessing, integration, and normalisation were performed using the Seurat package. Quality control was applied using the following filtering criteria: 300 < nFeature_RNA < 3000, red blood cell percentage < 5%, and mitochondrial percentage < 20%. Cell‐type annotation was performed based on canonical marker genes reported in the literature [40, 41]. The Slingshot package [42] was utilised to infer the pseudotime trajectories of KCs and hepatocytes. Furthermore, the CellChat package [43] was employed to explore ligand‐receptor interactions among diverse cell populations.

### 2.9 Animal Models

The ALF mouse model was induced via intraperitoneal (i.p.) injection of 100 μg/kg LPS and 400 mg/kg D‐Gal. For the macrophage depletion model, control liposomes or clodronate liposomes (10 mL/kg; Yeasen, China) were intravenously (i.v.) injected via the tail vein 24 h prior to ALF modelling. For the neutrophil depletion model, mice were administered an isotype control IgG or an anti‐Ly6G antibody (clone 1A8; 250 μg per mouse, Bio X Cell, USA) via i.p. injection 48 h before ALF induction. For the neutrophil transfer model, mouse bone marrow‐derived neutrophils (5 × 10^6^ cells/200 μL) were intravenously injected into wild‐type (WT) mice 4 h before ALF modelling. For the in vivo lactate treatment model, the pH of the lactate solution was adjusted to 7.4 using NaOH. Following ALF induction, mice were administered lactate (120 mg/kg) via i.p. injection at 24 or 6 h before sample collection.

### 2.10 Haematoxylin and Eosin (H&E) Staining

Liver tissue sections were deparaffinised and rehydrated through a graded ethanol series, followed by rinsing in distilled water. For histological staining, the sections were stained with haematoxylin for 5 min, followed by differentiation in 1% acid alcohol and bluing in warm water. The sections were subsequently counterstained with eosin, dehydrated, and mounted using neutral balsam. All sections were imaged using a light microscope (Olympus, Japan).

### 2.11 Immunohistochemistry (IHC) and Immunofluorescence (IF) Staining

Liver tissue sections were deparaffinised, rehydrated through a graded ethanol series, and subjected to antigen retrieval. Following standard permeabilization and blocking procedures, the sections were incubated with specific primary antibodies overnight at 4°C in a humidified chamber. For IHC staining, the sections were subsequently incubated with a biotinylated secondary antibody for 30 min at room temperature. After washing with PBS, a streptavidin‐biotin‐peroxidase complex was added and incubated for 30 min at room temperature. The sections were then stained with a 3,3′‐diaminobenzidine (DAB) solution for 5 min, rinsed with tap water, and counterstained with haematoxylin. Following differentiation with acid alcohol and bluing in tap water, the sections were dehydrated and mounted. Bright‐field images were captured using a light microscope (Olympus, Japan).

For IF staining, the sections were incubated with fluorescence‐conjugated secondary antibodies (Servicebio, China) for 1 h at room temperature. Following PBS washes, nuclei were counterstained with DAPI, and the fluorescence signals were observed and captured under a confocal microscope (Olympus, Japan).

### 2.12 Extraction of NETs

Following treatment with PMA for 4 h, the culture medium of dHL‐60 cells was gently discarded. The bottom of the culture dish was washed with pre‐chilled PBS, and the cells were collected into a centrifuge tube by gentle pipetting. After centrifugation at 400 g for 5 min at 4°C, the supernatant was carefully collected while the cell pellet was discarded. The obtained supernatant was further centrifuged at 16,000 rpm for 15 min at 4°C to precipitate the NETs. The NETs pellet was then resuspended in an appropriate volume of PBS, and the precise concentration was determined using a NanoDrop spectrophotometer (Sigma‐Aldrich, USA).

### 2.13 Protein Docking Analysis

The structural data of human KLF15 (UniProt ID: Q9UIH9) and AJUBA (UniProt ID: Q96IF1) were retrieved from the AlphaFold Protein Structure Database (https://alphafold.ebi.ac.uk/) [44]. Following pre‐processing and optimization, the PDB files were submitted to the HDOCK online server (http://hdock.phys.hust.edu.cn/) for protein–protein docking simulations [45]. Based on the algorithm's evaluation criteria, an absolute docking score above 200 coupled with a confidence score exceeding 0.7 indicates a highly probable physical interaction between the two proteins.

### 2.14 5‐Ethynyl‐2′‐Deoxyuridine (EdU) Incorporation Assay

Cell proliferation was evaluated using an EdU cell proliferation kit (Beyotime, China) according to the manufacturer's instructions. Briefly, HepG2 and AML12 cells were incubated with the EdU working solution for 6 h. Following PBS washes, the cells were fixed with 4% paraformaldehyde (PFA) for 15 min and permeabilized with 0.3% Triton X‐100 for 10 min. After washing, the cells were incubated with the EdU reaction cocktail for 30 min in the dark. Finally, the cells were washed with PBS, and the nuclei were counterstained with Hoechst 33342 for 10 min at room temperature. The fluorescence signals were observed and captured using a fluorescence microscope (Olympus, Japan).

### 2.15 Cell Transduction

Human embryonic kidney 293T (HEK293T) cells were maintained in DMEM supplemented with 10% FBS at 37°C in a 5% CO_2_ atmosphere. To generate lentiviral particles, HEK293T cells were co‐transfected with lentiviral transfer plasmids, encoding FLAG‐tagged KLF15 overexpression (OE), HA‐tagged AJUBA OE, or an AJUBA knockdown (Sh), alongside the requisite packaging plasmids using Lipofectamine 3000 (Invitrogen, USA). Viral supernatants were harvested at 48 h post‐transfection, filtered, and mixed with fresh culture medium at a 2:1 ratio. For viral transduction, HepG2 or AML12 cells were incubated with the viral mixture supplemented with 10 μg/mL Polybrene. Following 36 h of transduction, the medium was replaced, and stably transduced cells were selected using 2 μg/mL puromycin for 48 h prior to use in downstream experiments.

### 2.16 Statistical Analysis

All statistical analyses were performed using GraphPad Prism (v9.0) software. Quantitative data are presented as the mean ± standard deviation. Differences between two independent groups were evaluated using an unpaired Student's *t*‐test. For comparisons among three or more groups, a one‐way analysis of variance (ANOVA) followed by Tukey's post hoc test was conducted. Statistical significance was defined as follows: *p < 0.05, **p < 0.01, and ***p < 0.001; ‘ns’ denotes no significant difference.

## 3 Results

### 3.1 Lactate Uptake by Neutrophils Drives NETosis

Lactate serves as a crucial metabolic intermediate connecting glycolysis to the tricarboxylic acid (TCA) cycle, playing a pivotal role in immunomodulation and broader cellular functions [46]. Recent studies have highlighted that mitochondrial uptake and utilisation of lactate can impair mitochondrial integrity and exacerbate reactive oxygen species (ROS) production. Furthermore, lactate exerts regulatory control over cellular functions via PTMs, specifically by altering the lactylation levels of mitochondrial proteins [47, 48]. To investigate whether neutrophils can internalise exogenous lactate, we co‐incubated dHL‐60 cells with Cy3‐labelled lactate (Cy3‐lactate) and labelled the cell membranes with PKH67. Observations at 2, 4, 8, 12, and 24 h revealed that lactate was internalised into dHL‐60 cells as early as 2 h, with intracellular accumulation increasing in a time‐dependent manner (Figure 1A). To precisely determine the subcellular localization of the internalised lactate, we counterstained the dHL‐60 cells with Mito‐Tracker. The results demonstrated a high degree of spatial co‐localization between Cy3‐lactate and mitochondria, indicating that exogenous lactate is predominantly internalised into the mitochondria of dHL‐60 cells to modulate cellular functions. Furthermore, this uptake was partially abrogated by pre‐treatment with the MCT1 inhibitor AZD3965 (Figure 1B). To validate these findings, we isolated mouse bone marrow‐derived neutrophils and co‐incubated them with Cy3‐lactate, Mito‐Tracker, and PKH67. Consistent with the dHL‐60 cells, we observed the mitochondrial internalisation of lactate in neutrophils, which was similarly attenuated by AZD3965 treatment (Figure 1C).

**FIGURE 1:**
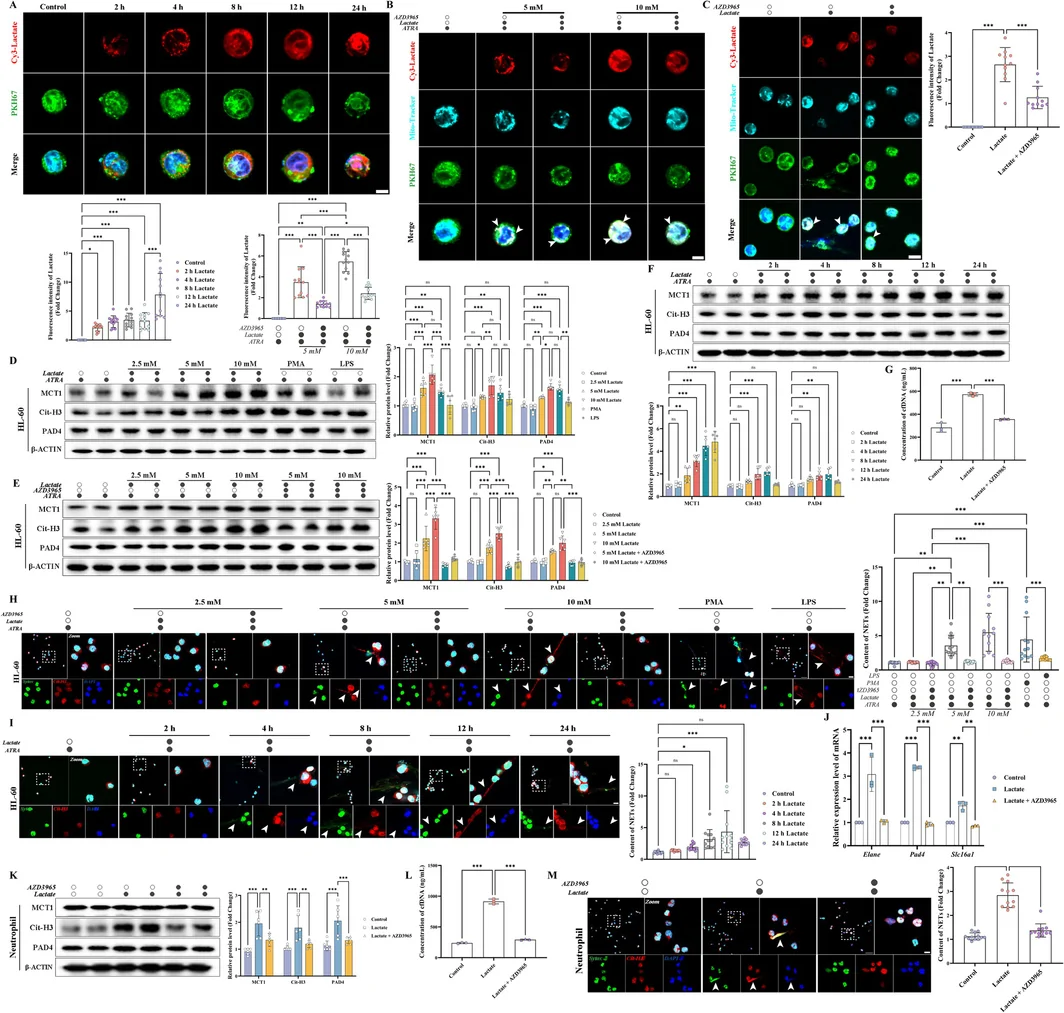
Lactate uptake by neutrophils drives NETosis. (A) IF staining of Cy3‐Lactate, PKH67, and Hoechst 33342 in dHL‐60 cells from the control, 2 h, 4 h, 8 h, 12 h, and 24 h lactate groups (*n* = 12). Scale bar = 10 μm. (B) IF staining of Cy3‐Lactate, Mito‐Tracker, PKH67, and Hoechst 33342 in dHL‐60 cells from the control, 5 mM lactate, 5 mM lactate + AZD3965, 10 mM lactate, and 10 mM lactate + AZD3965 groups (*n* = 12). Scale bar = 10 μm. (C) IF staining of Cy3‐Lactate, Mito‐Tracker, PKH67, and Hoechst 33342 in mouse bone marrow‐derived neutrophils from the control, 5 mM lactate, 5 mM lactate + AZD3965, 10 mM lactate, and 10 mM lactate + AZD3965 groups (*n* = 11). Scale bar = 10 μm. (D) WB analysis of MCT1, Cit‐H3, and PAD4 protein levels in dHL‐60 cells from the control, 2.5 mM lactate, 5 mM lactate, 10 mM lactate, PMA, and LPS groups (*n* = 6). (E) WB analysis of MCT1, Cit‐H3, and PAD4 protein levels in dHL‐60 cells from the control, 2.5 mM lactate, 5 mM lactate, 10 mM lactate, 5 mM lactate + AZD3965, and 10 mM lactate + AZD3965 groups (*n* = 6). (F) WB analysis of MCT1, Cit‐H3, and PAD4 protein levels in dHL‐60 cells from the control, 2 h, 4 h, 8 h, 12 h, and 24 h lactate groups (*n* = 6). (G) Levels of cfDNA in the culture supernatants of dHL‐60 cells from the control, lactate, and lactate + AZD3965 groups (*n* = 3). (H) IF staining of Sytox Green, Cit‐H3, and DAPI in dHL‐60 cells from the control, 2.5 mM lactate, 2.5 mM lactate + AZD3965, 5 mM lactate, 5 mM lactate + AZD3965, 10 mM lactate, 10 mM lactate + AZD3965, PMA, and LPS groups (*n* = 12). Scale bar = 10 μm. (I) IF staining of Sytox Green, Cit‐H3, and DAPI in dHL‐60 cells from the control, 2 h, 4 h, 8 h, 12 h, and 24 h lactate groups (*n* = 12). Scale bar = 10 μm. (J) qRT‐PCR analysis of *Elane* (*Ne*), *Pad4*, and *Slc16a1* (*Mct1*) mRNA levels in mouse neutrophils from the control, lactate, and lactate + AZD3965 groups (*n* = 3). (K) WB analysis of MCT1, Cit‐H3, and PAD4 protein levels in mouse neutrophils from the control, lactate, and lactate + AZD3965 groups (*n* = 6). (L) Levels of cfDNA in the culture supernatants of mouse neutrophils from the control, lactate, and lactate + AZD3965 groups (*n* = 3). (M) IF staining of Sytox Green, Cit‐H3, and DAPI in mouse neutrophils from the control, lactate, and lactate + AZD3965 groups (*n* = 12). Scale bar = 10 μm. (White dots ‘○’ represent the untreated groups, while black dots ‘●’ denote the groups receiving the indicated treatment.).

It has been previously reported that upon phagocytosing *Staphylococcus*, neutrophils can sense the bacteria‐derived lactate to induce NETosis [49]. Therefore, we further evaluated the activation of NETosis in response to varying concentrations of exogenous lactate. WB analysis revealed that treatment with 10 mM lactate elicited the highest protein levels of citrullinated histone H3 (Cit‐H3) and peptidylarginine deiminase 4 (PAD4) in dHL‐60 cells. This induction was comparable to that achieved with 100 nM PMA and was greater than that induced by 1 μg/mL LPS, suggesting that 10 mM lactate serves as a potent inducer of robust NETosis (Figure 1D). Blockade of lactate uptake using the MCT1 inhibitor AZD3965 downregulated the protein levels of Cit‐H3 and PAD4 (Figure 1E). A time‐course analysis demonstrated that the levels of Cit‐H3 and PAD4 increased progressively with prolonged lactate exposure, peaking at 12 h, with no further significant elevation observed when the treatment was extended to 24 h (Figure 1F). In addition, we quantified the levels of cell‐free DNA (cfDNA) in the culture supernatants of dHL‐60 cells, confirming that lactate treatment robustly induced cfDNA release, which was blunted by AZD3965 (Figure 1G). Furthermore, IF co‐staining using Cit‐H3, Sytox Green, and DAPI was performed to visualise NETs release. Consistent with the WB results, 10 mM lactate triggered prominent NETs formation, peaking at 12 h, while AZD3965 treatment partially rescued this phenotype (Figure 1H,I). Accordingly, upon lactate stimulation, mouse neutrophils exhibited elevated mRNA levels of *Elane* (*Ne*), *Pad4*, and *Slc16a1* (*Mct1*), as well as increased protein levels of MCT1, Cit‐H3, and PAD4. This was accompanied by augmented cfDNA release and IF staining of NETs, all of which were suppressed by AZD3965 treatment (Figure 1J–M). Taken together, these data robustly demonstrate that neutrophils internalise lactate into mitochondria, thereby triggering NETosis.

### 3.2 Hepatic Kupffer Cells Release Massive Amounts of Lactate via Enhanced Glycolysis During ALF

To further explore the association between lactate‐activated NETosis and the onset and progression of ALF, we analysed the ALF mouse liver tissue RNA‐seq dataset GSE217659. Using cutoff thresholds of |log_2_FC| > 1 and *p* < 0.05, we identified 1977 downregulated and 3669 upregulated DEGs. GO enrichment analysis revealed that these DEGs were significantly enriched in immune activation and immune cell migration pathways (Figure 2A,B). Given that glycolysis reprogramming is a hallmark of immune cell activation, we further identified glycolysis‐related DEGs within this dataset (Figure 2C). Next, we analysed mouse liver scRNA‐seq data. Following standard quality control, dimensionality reduction, and cell annotation procedures, 11 distinct cell clusters were identified (Figure S1C, Figure 2D). Using the previously identified glycolysis‐related DEGs as specific markers, we found that the overall expression of glycolysis‐related genes within KCs was significantly elevated in the ALF group (Figure 2E). Metabolic pathway analysis indicated that the glycolysis pathway was upregulated while the TCA cycle downregulated in KCs during ALF (Figure S1D,E). We further stratified the KCs into glycolysis‐positive (KC_glycolysis_pos) and negative (KC_glycolysis_neg) subclusters based on the expression levels of these DEGs. Notably, the KC_glycolysis_pos subcluster expanded significantly during ALF (Figure 2F, Figure S1F). Pseudotime trajectory analysis demonstrated that the expression dynamics of *Hk2* and *Ldha* closely aligned with the KC_glycolysis_pos subcluster, whereas *Ldhb* distribution aligned with the KC_glycolysis_neg subcluster (Figure 2G,H). Furthermore, cell–cell communication network analysis indicated a robust interaction between the KC_glycolysis_pos subcluster and neutrophils, particularly via the Ccl5‐Ccr1 signalling axis, which is fundamentally associated with neutrophil recruitment [50] (Figure 2I,J).

**FIGURE 2:**
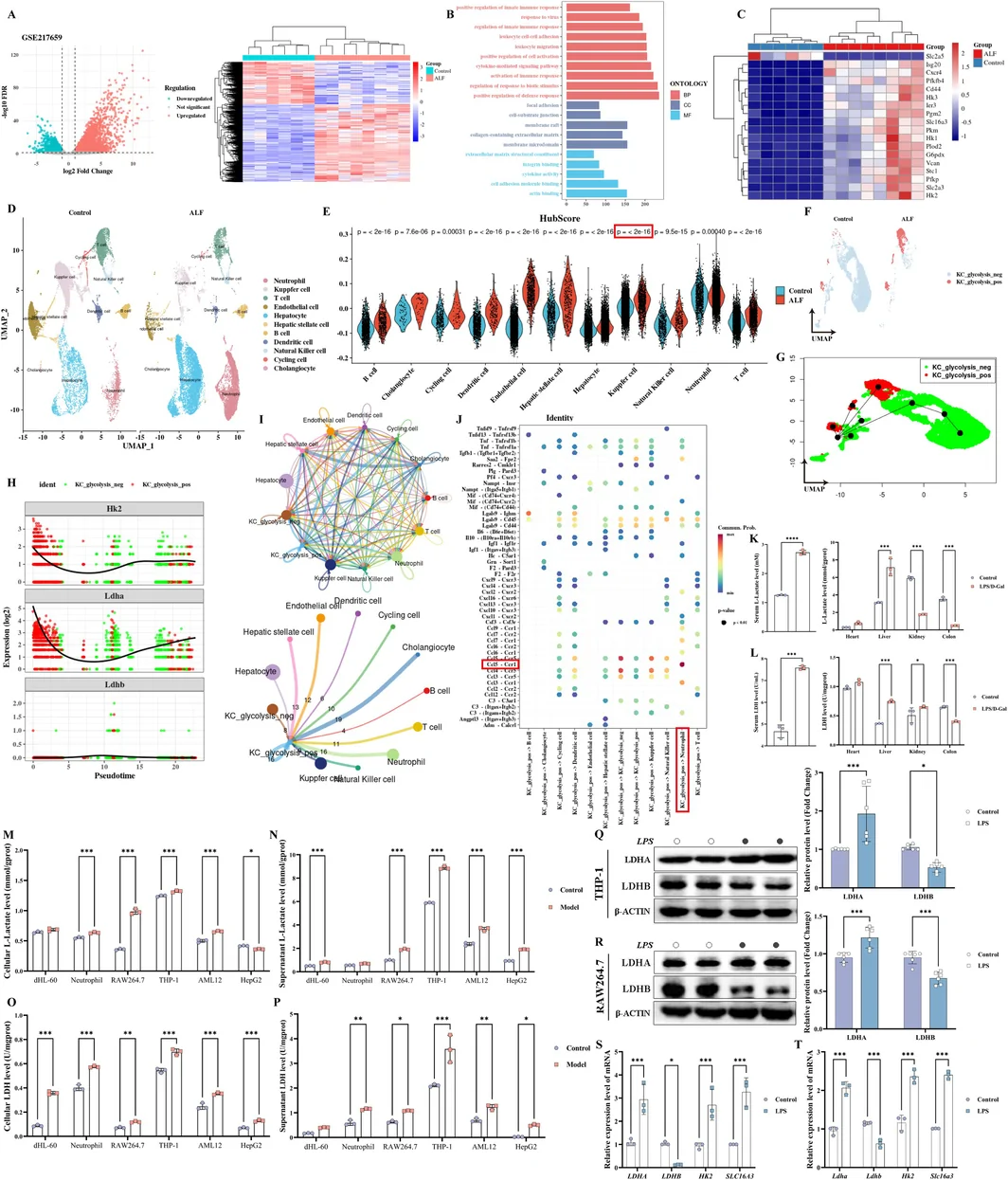
Hepatic Kupffer cells release massive amounts of lactate via enhanced glycolysis during ALF. (A) Volcano plot (left) and heatmap (right) illustrating DEGs identified from RNA‐seq of liver tissues from control and ALF mice. (B) GO enrichment analysis revealing the significantly enriched signalling pathways of the DEGs. (C) Heatmap depicting the expression profiles of glycolysis‐related DEGs in ALF. (D) UMAP plot visualisation identifying 11 cell clusters in the scRNA‐seq dataset of murine liver tissues. (E) Violin plots displaying the expression levels of the glycolysis‐related gene signature across different cell clusters in the control and ALF groups. (F) UMAP plot illustrating the distribution and abundance of the KC_glycolysis_pos and KC_glycolysis_neg clusters in control and ALF groups. (G‐H) Pseudotime trajectory analysis mapping the expression kinetics of *Hk2*, *Ldha*, and *Ldhb* along the differentiation lineages of the KC_glycolysis_pos and KC_glycolysis_neg subsets. (I) Circle plot illustrating the intercellular communication network between the KC_glycolysis_pos subpopulation and other cell clusters. (J) Bubble plot summarizing the significant signalling pathways involved in the crosstalk between the KC_glycolysis_pos subpopulation and other cell clusters. (K) Quantification of L‐lactate levels in the heart, liver, kidney, colon, and serum of mice from the control and LPS/D‐Gal groups (*n* = 3). (L) Quantification of LDH activity in the heart, liver, kidney, colon, and serum of mice from the control and LPS/D‐Gal groups (*n* = 3). (M‐N) Intracellular and culture supernatant L‐lactate levels in dHL‐60 cells, mouse neutrophils, RAW264.7, THP‐1, AML12, and HepG2 cells following respective modelling (*n* = 3). (O‐P) Intracellular and culture supernatant LDH activity in dHL‐60 cells, mouse neutrophils, RAW264.7, THP‐1, AML12, and HepG2 cells following respective modelling (*n* = 3). (Q) WB analysis of LDHA and LDHB protein levels in THP‐1 cells from the control and LPS groups (*n* = 6). (R) WB analysis of LDHA and LDHB protein levels in RAW264.7 cells from the control and LPS groups (*n* = 6). (S) qRT‐PCR analysis of *LDHA*, *LDHB*, *HK2*, and *SLC16A3* (*MCT4*) mRNA levels in THP‐1 cells from the control and LPS groups (*n* = 3). (T) qRT‐PCR analysis of *Ldha*, *Ldhb*, *Hk2*, and *Slc16a3* mRNA levels in RAW264.7 cells from the control and LPS groups (*n* = 3). (White dots ‘○’ represent the untreated groups, while black dots ‘●’ denote the groups receiving the indicated treatment.).

To validate the pivotal regulatory role of KCs in hepatic lactate homeostasis, we quantified L‐lactate and LDH levels across the heart, liver, kidney, colon, and serum. We observed that lactate and LDH levels in the liver and serum were most drastically elevated in the LPS/D‐Gal‐induced ALF model (Figure 2K,L). At the cellular level, we respectively treated dHL‐60 cells and primary neutrophils with PMA, RAW264.7 and THP‐1 cells with LPS, and AML12 and HepG2 cells with LPS/D‐Gal. Intracellular and supernatant levels of lactate and LDH were subsequently measured. While lactate levels were elevated across all evaluated cell types in the model groups, RAW264.7 cells exhibited the most pronounced intracellular lactate accumulation, and THP‐1 cells showed the most significant increase in supernatant lactate. These findings strongly suggest that macrophages produce and release massive amounts of lactate upon inflammatory stimulation (Figure 2M,N). Although intracellular LDH levels in both THP‐1 and RAW264.7 cells substantially increased following LPS stimulation, these elevations were not the most pronounced among all the cells tested. This phenomenon may be partially attributed to the rapid release of intracellular LDH into the culture supernatant following cellular activation and membrane permeabilization (Figure 2O,P). Moreover, we assessed the protein and mRNA expression levels of LDHA and LDHB in both THP‐1 and RAW264.7 cells. LPS treatment resulted in the upregulation of LDHA and the concurrent downregulation of LDHB, indicating enhanced lactate production and diminished conversion to pyruvate [51] (Figure 2Q–T). Additionally, the mRNA levels of *SLC16A3 (MCT4)* were elevated in the LPS group, pointing to an augmented lactate efflux capacity in activated macrophages [52].

### 3.3 Macrophage Depletion Attenuates NETosis and Ameliorates Liver Injury

To further evaluate the impact of macrophage‐derived lactate release on NETosis and hepatic function during ALF, we established a macrophage depletion mouse model via tail vein injection of clodronate liposomes. H&E staining revealed that mice in the LPS/D‐Gal + Lip/PBS group exhibited profound hepatic congestion, inflammatory cell infiltration, and architectural distortion, all of which were ameliorated in the LPS/D‐Gal + Lip/Clo group. The expression of F4/80, a canonical macrophage marker, was significantly elevated in the LPS/D‐Gal + Lip/PBS group but drastically reduced in the LPS/D‐Gal + Lip/Clo group. The variation in Ly6G, a neutrophil marker, paralleled that of F4/80 (Figure 3A). These findings suggest that macrophage depletion curtails neutrophil infiltration and improves hepatic morphological abnormalities. Furthermore, lactate and LDH levels were increased in the LPS/D‐Gal + Lip/PBS group but significantly diminished in the LPS/D‐Gal + Lip/Clo group (Figure 3B,C). Regarding hepatic function, the elevated liver enzyme levels observed in the LPS/D‐Gal + Lip/PBS group were effectively reversed in the LPS/D‐Gal + Lip/Clo group (Figure 3D). WB analysis also demonstrated that the protein levels of LDHA, Cit‐H3, and PAD4 were augmented in the LPS/D‐Gal + Lip/PBS group and markedly decreased in the LPS/D‐Gal + Lip/Clo group. Conversely, the protein level of LDHB was reduced in the LPS/D‐Gal + Lip/PBS group and restored in the LPS/D‐Gal + Lip/Clo group (Figure 3E). These results indicate that macrophages are the predominant source of glycolysis during ALF, and that variations in lactate levels are closely correlated with the extent of NETosis. In addition, IF staining of NETs and quantification of serum cfDNA levels confirmed that NETs release was prominently enhanced in the LPS/D‐Gal + Lip/PBS group but mitigated in the LPS/D‐Gal + Lip/Clo group (Figure 3F,G). qRT‐PCR further revealed that the mRNA levels of *Ldha*, *Hk2*, and *Slc16a3* were upregulated in the LPS/D‐Gal + Lip/PBS group and downregulated in the LPS/D‐Gal + Lip/Clo group (Figure 3H). The alterations in the mRNA levels of NETosis‐related genes (*Elane* and *Pad4*) and the lactate uptake‐related gene (*Slc16a1*) exhibited a consistent trend (Figure 3I). Collectively, these data indicate that macrophages produce lactate via glycolysis to activate NETosis during ALF, and blocking this cascade effectively ameliorates hepatic failure.

**FIGURE 3:**
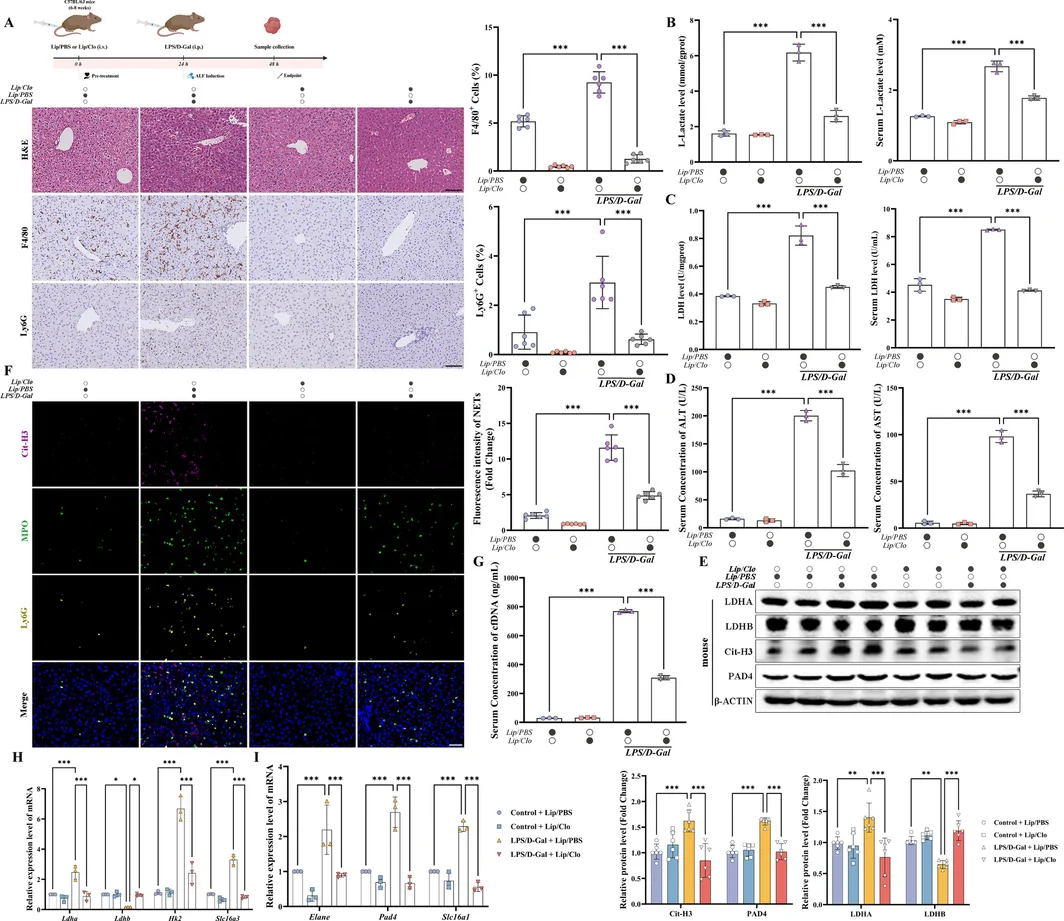
Macrophage depletion attenuates NETosis and ameliorates liver injury. (A) Liver H&E staining, and IHC staining of F4/80 and Ly6G in mice from the control + Lip/PBS, control + Lip/Clo, LPS/D‐Gal + Lip/PBS, and LPS/D‐Gal + Lip/Clo groups (*n* = 6). Scale bar = 50 μm. (B) Hepatic and serum levels of L‐lactate in mice from the control + Lip/PBS, control + Lip/Clo, LPS/D‐Gal + Lip/PBS, and LPS/D‐Gal + Lip/Clo groups (*n* = 3). (C) Hepatic and serum levels of LDH in mice from the control + Lip/PBS, control + Lip/Clo, LPS/D‐Gal + Lip/PBS, and LPS/D‐Gal + Lip/Clo groups (*n* = 3). (D) Serum levels of ALT and AST in mice from the control + Lip/PBS, control + Lip/Clo, LPS/D‐Gal + Lip/PBS, and LPS/D‐Gal + Lip/Clo groups (*n* = 3). (E) WB analysis of LDHA, LDHB, Cit‐H3, and PAD4 protein levels in liver tissues from the control + Lip/PBS, control + Lip/Clo, LPS/D‐Gal + Lip/PBS, and LPS/D‐Gal + Lip/Clo groups (*n* = 6). (F) IF staining of Mpo, Cit‐H3, Ly6G, and DAPI in liver tissues from the control + Lip/PBS, control + Lip/Clo, LPS/D‐Gal + Lip/PBS, and LPS/D‐Gal + Lip/Clo groups (*n* = 6). Scale bar = 20 μm. (G) Serum levels of cfDNA in mice from the control + Lip/PBS, control + Lip/Clo, LPS/D‐Gal + Lip/PBS, and LPS/D‐Gal + Lip/Clo groups (*n* = 3). (H) qRT‐PCR analysis of *Ldha*, *Ldhb*, *Hk2*, and *Slc16a3* mRNA levels in liver tissues from the control + Lip/PBS, control + Lip/Clo, LPS/D‐Gal + Lip/PBS, and LPS/D‐Gal + Lip/Clo groups (*n* = 3). (I) qRT‐PCR analysis of *Elane*, *Pad4*, and *Slc16a1* mRNA levels in liver tissues from the control + Lip/PBS, control + Lip/Clo, LPS/D‐Gal + Lip/PBS, and LPS/D‐Gal + Lip/Clo groups (*n* = 3). (White dots ‘○’ represent the untreated groups, while black dots ‘●’ denote the groups receiving the indicated treatment.).

### 3.4 Exogenous Lactate Administration Upregulates NETosis

To further validate the pivotal role of lactate in driving hepatic NETosis, mice were intraperitoneally injected with 120 mg/kg lactate for 6 and 24 h, respectively. H&E staining demonstrated that treatment with lactate alone did not induce substantial hepatic architectural distortion or congestion. However, the co‐administration of lactate with LPS/D‐Gal provoked more severe hepatic structural disruption compared to LPS/D‐Gal modelling alone. Consistently, the Lactate 24 h + LPS/D‐Gal group exhibited the most profound NETs release, which was significantly higher than that in the Lactate 6 h + LPS/D‐Gal group and the LPS/D‐Gal group. Notably, while lactate treatment alone failed to trigger overt hepatic morphological damage, it successfully upregulated NETs release, with the Lactate 24 h group exerting a stronger effect than the Lactate 6 h group (Figure 4A). In terms of hepatic function, liver enzyme levels peaked in the Lactate 24 h + LPS/D‐Gal group and were higher in the Lactate 6 h + LPS/D‐Gal group compared to the LPS/D‐Gal group (Figure 4B). Regarding NETosis, the Lactate 24 h + LPS/D‐Gal group displayed the highest levels of serum cfDNA, *Elane* and *Pad4* mRNA expression, as well as Cit‐H3 and PAD4 protein expression, followed successively by the Lactate 6 h + LPS/D‐Gal group and the LPS/D‐Gal group. Moreover, the Lactate 24 h group exhibited higher cfDNA levels, Cit‐H3 and PAD4 protein levels, and *Elane* and *Pad4* mRNA expression compared to the control group (Figure 4C–E). In summary, lactate is capable of inducing NETosis in vivo, and excessive lactate stimulation exacerbates liver injury and structural abnormalities during ALF.

**FIGURE 4:**
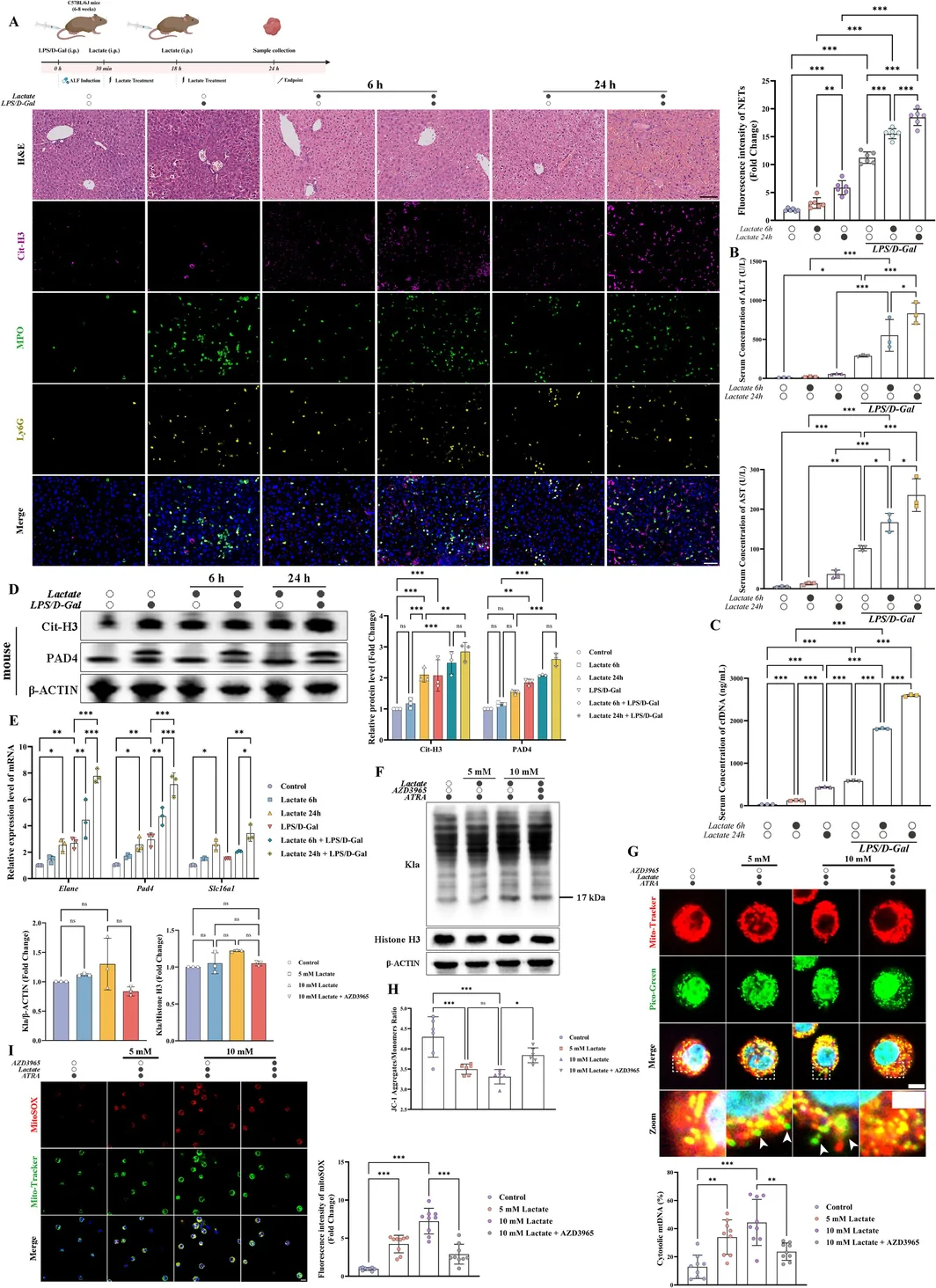
Exogenous lactate triggers NETosis and exacerbates hepatic dysfunction. (A) Liver H&E staining, and IF staining of Mpo, Cit‐H3, Ly6G, and DAPI in mice from the control + PBS, LPS/D‐Gal + PBS, Lactate 6 h + PBS, Lactate 6 h + LPS/D‐Gal, Lactate 24 h + PBS, and Lactate 24 h + LPS/D‐Gal groups (*n* = 6). Scale bar = 20 μm. (B) Serum levels of ALT and AST in mouse from the control + PBS, LPS/D‐Gal + PBS, Lactate 6 h + PBS, Lactate 6 h + LPS/D‐Gal, Lactate 24 h + PBS, and Lactate 24 h + LPS/D‐Gal groups (*n* = 3). (C) Serum levels of cfDNA in mouse from the control + PBS, LPS/D‐Gal + PBS, Lactate 6 h + PBS, Lactate 6 h + LPS/D‐Gal, Lactate 24 h + PBS, and Lactate 24 h + LPS/D‐Gal groups (*n* = 3). (D) WB analysis of Cit‐H3 and PAD4 protein levels in liver tissues from the control + PBS, LPS/D‐Gal + PBS, Lactate 6 h + PBS, Lactate 6 h + LPS/D‐Gal, Lactate 24 h + PBS, and Lactate 24 h + LPS/D‐Gal groups (*n* = 3). (E) qRT‐PCR analysis of *Elane*, *Pad4*, and *Slc16a1* mRNA levels in liver tissues from the control + PBS, LPS/D‐Gal + PBS, Lactate 6 h + PBS, Lactate 6 h + LPS/D‐Gal, Lactate 24 h + PBS, and Lactate 24 h + LPS/D‐Gal groups (*n* = 3). (F) WB analysis of Kla and Histone H3 protein levels in dHL‐60 cells from the control, 5 mM lactate, 10 mM lactate, and 10 mM lactate + AZD3965 groups (*n* = 3). (G) IF staining of Mito‐Tracker, Pico‐Green, and Hoechst 33342 in dHL‐60 cells from the control, 5 mM lactate, 10 mM lactate, and 10 mM lactate + AZD3965 groups (*n* = 9). Scale bar = 5 μm. (H) JC‐1 staining of dHL‐60 cells from the control, 5 mM lactate, 10 mM lactate, and 10 mM lactate + AZD3965 groups (*n* = 6). (I) IF staining of MitoSOX, Mito‐Tracker, and Hoechst 33342 in dHL‐60 cells from the control, 5 mM lactate, 10 mM lactate, and 10 mM lactate + AZD3965 groups (*n* = 9). Scale bar = 20 μm. (White dots ‘○’ represent the untreated groups, while black dots ‘●’ denote the groups receiving the indicated treatment.).

Recent studies have reported that lactate participates in various pathophysiological processes by modulating protein lactylation levels, oxidative homeostasis, and mitochondrial function. Therefore, we further investigated the specific mechanisms underlying lactate‐driven NETosis. It has been proposed that lactate may facilitate chromatin decondensation to promote NETosis via the lactylation of histone H3 at lysine 18 (H3K18la) [53]. However, WB analysis of lactyl‐lysine (Kla) levels in whole‐cell lysates of dHL‐60 cells revealed no significant changes in overall lactylation following lactate stimulation. Although histone lactylation at approximately 17 kDa was elevated upon treatment with 10 mM lactate (Figure 4F), our preceding results demonstrated that 5 mM lactate was sufficient to induce NETosis. This concentration discordance suggests that alterations in histone lactylation are not the primary driver for inducing NETosis in our study. Alternatively, lactate accumulation may induce mitochondrial DNA (mtDNA) damage and leakage, thereby activating the cGAS‐STING signalling pathway, which is intimately intertwined with inflammation and NETosis [54, 55]. Indeed, co‐staining with Mito‐Tracker and Pico‐Green revealed cytosolic mtDNA leakage in the 5 mM lactate group, which was further exacerbated at the 10 mM lactate group. Notably, this leakage was effectively attenuated by treatment with AZD3965 (Figure 4G). Consistently, JC‐1 staining indicated that the loss of mitochondrial membrane potential paralleled these findings (Figure 4H). Furthermore, intracellular lactate may be converted into pyruvate, subsequently generating ROS that exacerbate mitochondrial oxidative stress and trigger NETosis [49]. Accordingly, we assessed mitochondrial oxidative stress using the MitoSOX staining. The results demonstrated a marked elevation in mitochondrial ROS (mtROS) levels in the 10 mM lactate group, which was partially suppressed by AZD3965 (Figure 4I). Collectively, these findings indicate that lactate triggers mtDNA leakage and mtROS overproduction, culminating in NETosis and exacerbating hepatic injury during ALF.

### 3.5 NETs DNA Activates the TLR9 Signalling Pathway in Hepatocytes

Our in vivo findings from the macrophage depletion and lactate + LPS/D‐Gal mouse models indicated that the extent of NETosis paralleled the severity of liver injury. Therefore, we sought to determine which specific component of NETs mediates hepatocyte damage. To elucidate this, HepG2 cells were co‐incubated with NETs for 6, 12, or 24 h, and subsequently subjected to IF staining for Cit‐H3, MPO, NE, Mito‐Tracker, Sytox Green, and TOM20. At 6 h, abundant NETs DNA (visualised by Sytox Green and DAPI) was observed enveloping the periphery of the hepatocytes. As the incubation time extended to 24 h, the peri‐membrane DNA fluorescence diminished, concomitantly with the appearance of intracellular fluorescent puncta. This dynamic shift suggests that NETs DNA is a critical functional component internalised by hepatocytes (Figure 5A). To dynamically confirm this internalisation process, we performed co‐staining with Sytox Green, TOM20, and DAPI across the 6, 12, and 24 h time points, with or without the addition of DNase I. In both HepG2 and AML12 cells, we captured the progressive dynamic internalisation of NETs DNA from the extracellular membrane into the intracellular space over time, a process that was effectively abolished by DNase I‐mediated DNA degradation (Figure 5B,C). Cellular DNA sensing systems primarily include the cyclic GMP‐AMP synthase‐stimulator of interferon genes (cGAS‐STING) and TLR9 pathways [56]. While cGAS‐STING typically acts as a cytosolic DNA sensor, TLR9 is predominantly localized within the endosomal compartment to detect internalised DNA. Thus, we investigated the impact of NETs internalisation on the TLR9 signalling cascade. qRT‐PCR and WB analyses revealed that NETs treatment upregulated TLR9 expression in hepatocytes, an effect abrogated by DNase I. Furthermore, the protein levels of myeloid differentiation primary response 88 (MYD88) and phosphorylated nuclear factor‐κB p65 (p‐NFκB p65) were robustly elevated in the NETs group but substantially reduced in the NETs + DNase I group (Figure 5D–I). These data indicate that NETs DNA specifically activates the endosomal TLR9 signalling pathway in hepatocytes.

**FIGURE 5:**
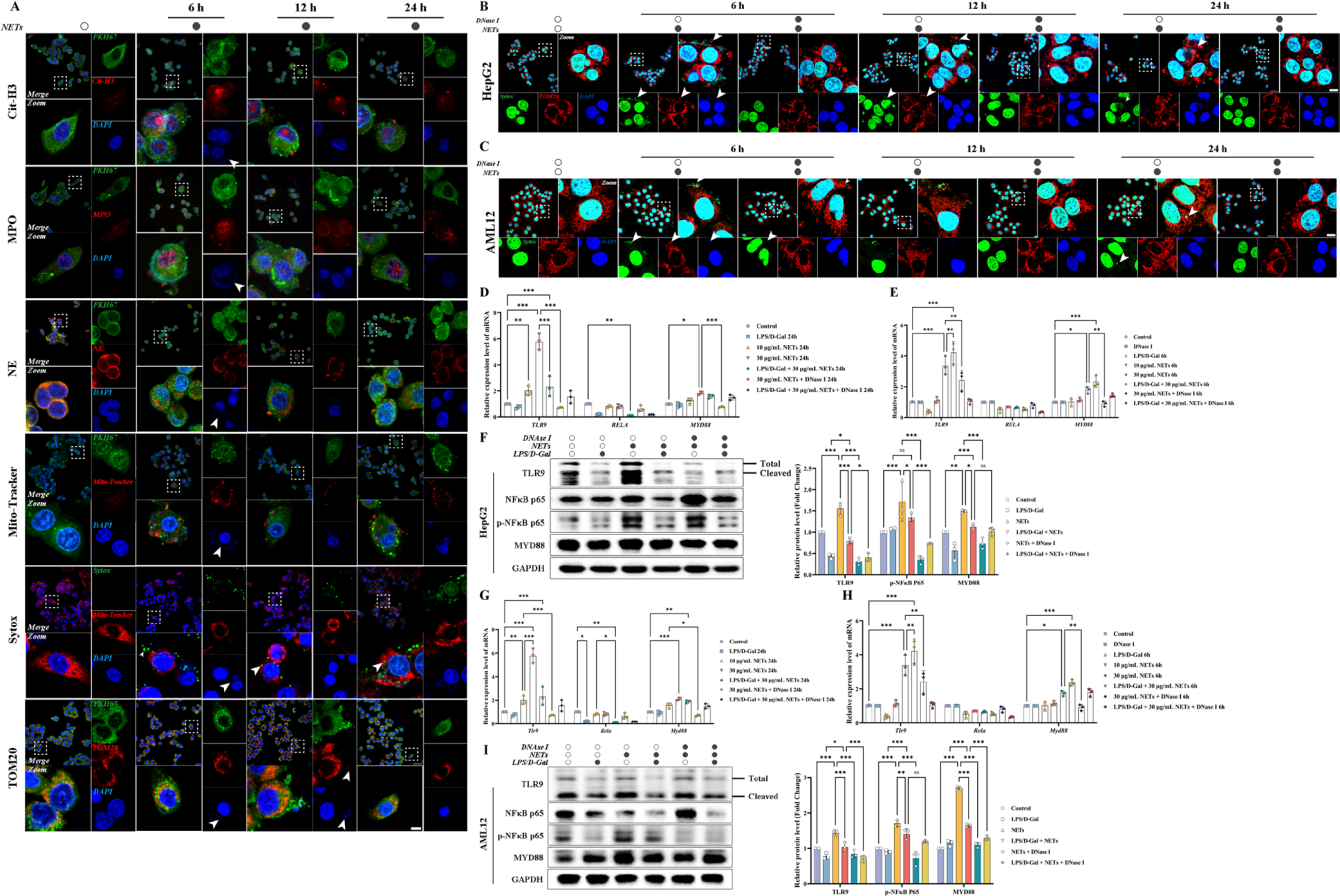
NETs DNA activates the TLR9 signalling pathway in hepatocytes. (A) IF staining of NETs components (Cit‐H3, MPO, NE, and Sytox Green) and mitochondria (MitoTracker and TOM20) in HepG2 cells from the control, NETs 6 h, NETs 12 h, and NETs 24 h groups. Scale bar = 10 μm. (B) IF staining of Sytox Green, TOM20, and DAPI in HepG2 cells from the control, NETs 6 h, NETs 6 h + DNase I, NETs 12 h, NETs 12 h + DNase I, NETs 24 h, and NETs 24 h + DNase I groups. Scale bar = 10 μm. (C) IF staining of Sytox Green, TOM20, and DAPI in AML12 cells from the control, NETs 6 h, NETs 6 h + DNase I, NETs 12 h, NETs 12 h + DNase I, NETs 24 h, and NETs 24 h + DNase I groups. Scale bar = 10 μm. (D) qRT‐PCR analysis of *TLR9*, *RELA*, and *MYD88* mRNA levels in HepG2 cells from the control, LPS/D‐Gal 24 h, 10 μg/mL NETs 24 h, 30 μg/mL NETs 24 h, LPS/D‐Gal + 30 μg/mL NETs 24 h, 30 μg/mL NETs + DNase I 24 h, and LPS/D‐Gal + 30 μg/mL NETs + DNase I 24 h groups (*n* = 3). (E) qRT‐PCR analysis of *TLR9*, *RELA*, and *MYD88* mRNA levels in HepG2 cells from the control, DNase I, LPS/D‐Gal 6 h, 10 μg/mL NETs 6 h, 30 μg/mL NETs 6 h, LPS/D‐Gal + 30 μg/mL NETs 6 h, 30 μg/mL NETs + DNase I 6 h, and LPS/D‐Gal + 30 μg/mL NETs + DNase I 6 h groups (*n* = 3). (F) WB analysis of TLR9, NFκB p65, p‐NFκB p65, and MYD88 protein levels in HepG2 cells from the control, LPS/D‐Gal, NETs, LPS/D‐Gal + NETs, NETs + DNase I, and LPS/D‐Gal + NETs + DNase I groups (*n* = 3). (G) qRT‐PCR analysis of *Tlr9*, *Rela*, and *Myd88* mRNA levels in AML12 cells from the control, LPS/D‐Gal 24 h, 10 μg/mL NETs 24 h, 30 μg/mL NETs 24 h, LPS/D‐Gal + 30 μg/mL NETs 24 h, 30 μg/mL NETs + DNase I 24 h, and LPS/D‐Gal + 30 μg/mL NETs + DNase I 24 h groups (*n* = 3). (H) qRT‐PCR analysis of *Tlr9*, *Rela*, and *Myd88* mRNA levels in AML12 cells from the control, DNase I, LPS/D‐Gal 6 h, 10 μg/mL NETs 6 h, 30 μg/mL NETs 6 h, LPS/D‐Gal + 30 μg/mL NETs 6 h, 30 μg/mL NETs + DNase I 6 h, and LPS/D‐Gal + 30 μg/mL NETs + DNase I 6 h groups (*n* = 3). (I) WB analysis of TLR9, NFκB p65, p‐NFκB p65, and MYD88 protein levels in AML12 cells from the control, LPS/D‐Gal, NETs, LPS/D‐Gal + NETs, NETs + DNase I, and LPS/D‐Gal + NETs + DNase I groups (*n* = 3). (White dots ‘○’ represent the untreated groups, while black dots ‘●’ denote the groups receiving the indicated treatment).

To further validate the regulatory role of NETosis in liver injury and the TLR9 pathway in vivo, we established a neutrophil depletion mouse model via the i.p. injection of the anti‐Ly6G antibody (clone 1A8). Compared to the LPS/D‐Gal + IgG group, mice in the LPS/D‐Gal +1A8 group exhibited alleviated hepatic congestion, improved structural integrity, and reduced NETs release (Figure 6A). Additionally, serum liver enzyme and cfDNA levels were significantly decreased in the LPS/D‐Gal + 1A8 group relative to the LPS/D‐Gal + IgG group (Figure 6B,C). qRT‐PCR and WB assays further demonstrated that the protein levels of Cit‐H3, PAD4, TLR9, MYD88, and p‐NFκB p65 were significantly elevated in the LPS/D‐Gal + IgG group but diminished in the LPS/D‐Gal +1A8 group, indicating that mitigating NETosis attenuates TLR9 pathway activation (Figure 6D–G).

**FIGURE 6:**
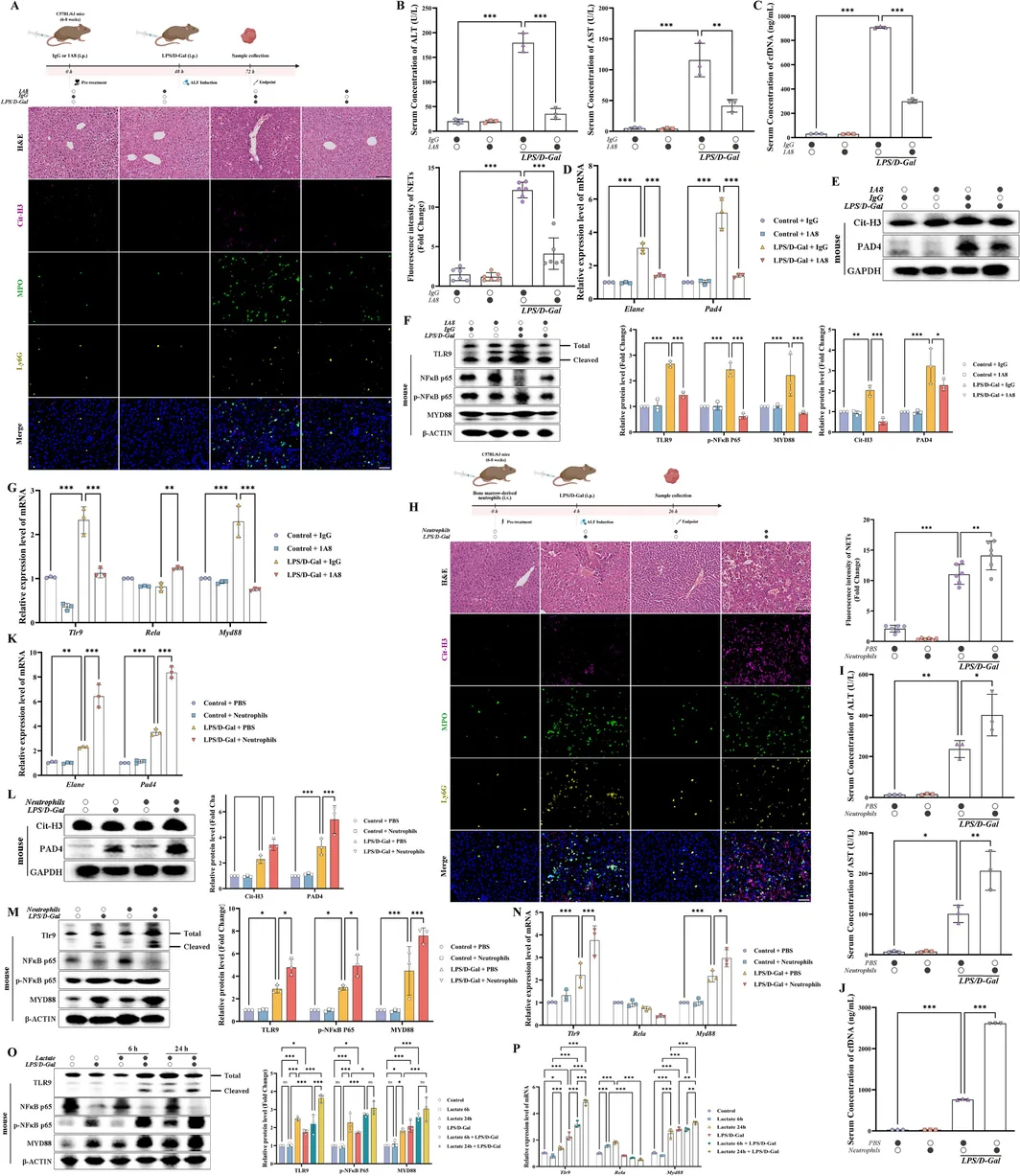
NETs DNA provokes the TLR9 pathway in vivo. (A) Liver H&E staining, and IF staining of Mpo, Cit‐H3, Ly6G, and DAPI in liver tissues from the control + IgG, LPS/D‐Gal + IgG, control + 1A8, and LPS/D‐Gal + 1A8 groups (*n* = 6). Scale bar = 20 μm. (B) Serum levels of ALT and AST in mouse from the control + IgG, LPS/D‐Gal + IgG, control +1A8, and LPS/D‐Gal +1A8 groups (*n* = 3). (C) Serum levels of cfDNA in mouse from the control + IgG, LPS/D‐Gal + IgG, control + 1A8, and LPS/D‐Gal + 1A8 groups (*n* = 3). (D) qRT‐PCR analysis of *Elane*, *Pad4*, and *Slc16a1* mRNA levels in liver tissues from the control + IgG, control + 1A8, LPS/D‐Gal + IgG, and LPS/D‐Gal + 1A8 groups (*n* = 3). (E) WB analysis of Cit‐H3 and PAD4 protein levels in liver tissues from the control + IgG, control + 1A8, LPS/D‐Gal + IgG, and LPS/D‐Gal + 1A8 groups (*n* = 3). (F) WB analysis of TLR9, NFκB p65, p‐NFκB p65, and MYD88 protein levels in liver tissues from the control + IgG, control + 1A8, LPS/D‐Gal + IgG, and LPS/D‐Gal + 1A8 groups (*n* = 3). (G) qRT‐PCR analysis of *Tlr9*, *Rela*, and *Myd88* mRNA levels in liver tissues from the control + IgG, control + 1A8, LPS/D‐Gal + IgG, and LPS/D‐Gal + 1A8 groups (*n* = 3). (H) Liver H&E staining, and IF staining of Mpo, Cit‐H3, Ly6G, and DAPI in the control + PBS, LPS/D‐Gal + PBS, control + Neutrophils, and LPS/D‐Gal + Neutrophils groups (*n* = 6). Scale bar = 20 μm. (I) Serum levels of ALT and AST in mouse from the control + PBS, LPS/D‐Gal + PBS, control + Neutrophils, and LPS/D‐Gal + Neutrophils groups (*n* = 3). (J) Serum levels of cfDNA in mouse from the control + PBS, LPS/D‐Gal + PBS, control + Neutrophils, and LPS/D‐Gal + Neutrophils groups (*n* = 3). (K) qRT‐PCR analysis of *Elane*, *Pad4*, and *Slc16a1* mRNA levels in liver tissue from the control + PBS, LPS/D‐Gal + PBS, control + Neutrophils, and LPS/D‐Gal + Neutrophils groups (*n* = 3). (L) WB analysis of Cit‐H3 and PAD4 protein levels in liver tissue from the control + PBS, LPS/D‐Gal + PBS, control + Neutrophils, and LPS/D‐Gal + Neutrophils groups (*n* = 3). (M) WB analysis of TLR9, NFκB p65, p‐NFκB p65, and MYD88 protein levels in liver tissue from the control + PBS, LPS/D‐Gal + PBS, control + Neutrophils, and LPS/D‐Gal + Neutrophils groups (*n* = 3). (N) qRT‐PCR analysis of *Tlr9*, *Rela*, and *Myd88* mRNA levels in the control + PBS, LPS/D‐Gal + PBS, control + Neutrophils, and LPS/D‐Gal + Neutrophils groups (*n* = 3). (O) WB analysis of TLR9, NFκB p65, p‐NFκB p65, and MYD88 protein levels in liver tissues from the control + PBS, LPS/D‐Gal + PBS, Lactate 6 h + PBS, Lactate 6 h + LPS/D‐Gal, Lactate 24 h + PBS, and Lactate 24 h + LPS/D‐Gal groups (*n* = 3). (P) qRT‐PCR analysis of *Elane*, *Pad4*, and *Slc16a1* mRNA levels in liver tissue from the control + PBS, LPS/D‐Gal + PBS, Lactate 6 h + PBS, Lactate 6 h + LPS/D‐Gal, Lactate 24 h + PBS, and Lactate 24 h + LPS/D‐Gal groups (*n* = 3). (White dots ‘○’ represent the untreated groups, while black dots ‘●’ denote the groups receiving the indicated treatment.).

Moreover, we performed an adoptive transfer of mouse bone marrow‐derived neutrophils via i.v. injection. While the transfer of neutrophils alone did not induce overt liver injury, it exacerbated hepatic structural distortion when coupled with LPS/D‐Gal modelling. Regarding NETs release, the LPS/D‐Gal + Neutrophils group displayed significantly more robust NETosis (Figure 6H). Consistently, serum ALT, AST and cfDNA levels were further elevated in the LPS/D‐Gal + Neutrophils group compared to the LPS/D‐Gal + PBS group (Figure 6I,J). Correspondingly, the mRNA levels of *Elane*, *Pad4*, *Tlr9*, and *Myd88*, as well as the protein levels of Cit‐H3, PAD4, TLR9, MYD88, and p‐NFκB p65, exhibited the same exacerbating trend (Figure 6K–N). Moreover, in the lactate treatment model, the protein levels of TLR9, MYD88, and p‐NFκB p65 peaked in the Lactate 24 h + LPS/D‐Gal group, followed by the Lactate 6 h + LPS/D‐Gal group, both of which were significantly higher than the LPS/D‐Gal group (Figure 6O,P). Taken together, these data robustly demonstrate that NETs DNA aggravates liver injury by activating the TLR9 signalling pathway in hepatocytes.

### 3.6 KLF15 Acts as a Pivotal Regulator in Liver Regeneration

Liver exhibits profound regenerative potential following PHx [57]. Conversely, ALF is frequently accompanied by impaired hepatic regeneration and repair, which constitutes a critical factor leading to the failure of liver function recovery. Therefore, we sought to explore the key DEGs distinguishing PHx from ALF. Using the mouse liver tissue RNA‐seq dataset GSE158864 with cutoff thresholds of |log_2_FC| > 1 and *p* < 0.05, we identified 1559 downregulated and 2021 upregulated DEGs. Notably, pathways related to repair and proliferation were significantly enriched during PHx (Figure 7A–C). We subsequently intersected the DEGs from ALF and PHx, identifying 173 genes upregulated in PHx but downregulated in ALF, and 277 genes downregulated in PHx but upregulated in ALF. The former gene set was primarily enriched in repair and regeneration pathways, whereas the latter was enriched in inflammation and leukocyte migration (Figure 7D). Motif prediction analysis of the 173 genes (upregulated in PHx while downregulated in ALF) using HOMER revealed the top 14 predicted transcription factors. Among them, only *Klf15* exhibited significant differential expression. Additionally, among its potential downstream targets, *Ajuba* and *Wnt9b* were upregulated during PHx but downregulated during ALF (Figure 7E,F). Correspondingly, the pseudotime trajectory analysis of extracted hepatocyte subclusters from mouse PHx scRNA‐seq data demonstrated that KLF15 distribution aligned consistently with the differentiation potential of hepatocytes, hinting at a crucial link between KLF15 and liver regeneration (Figure 7G,H, Figure S2A).

**FIGURE 7:**
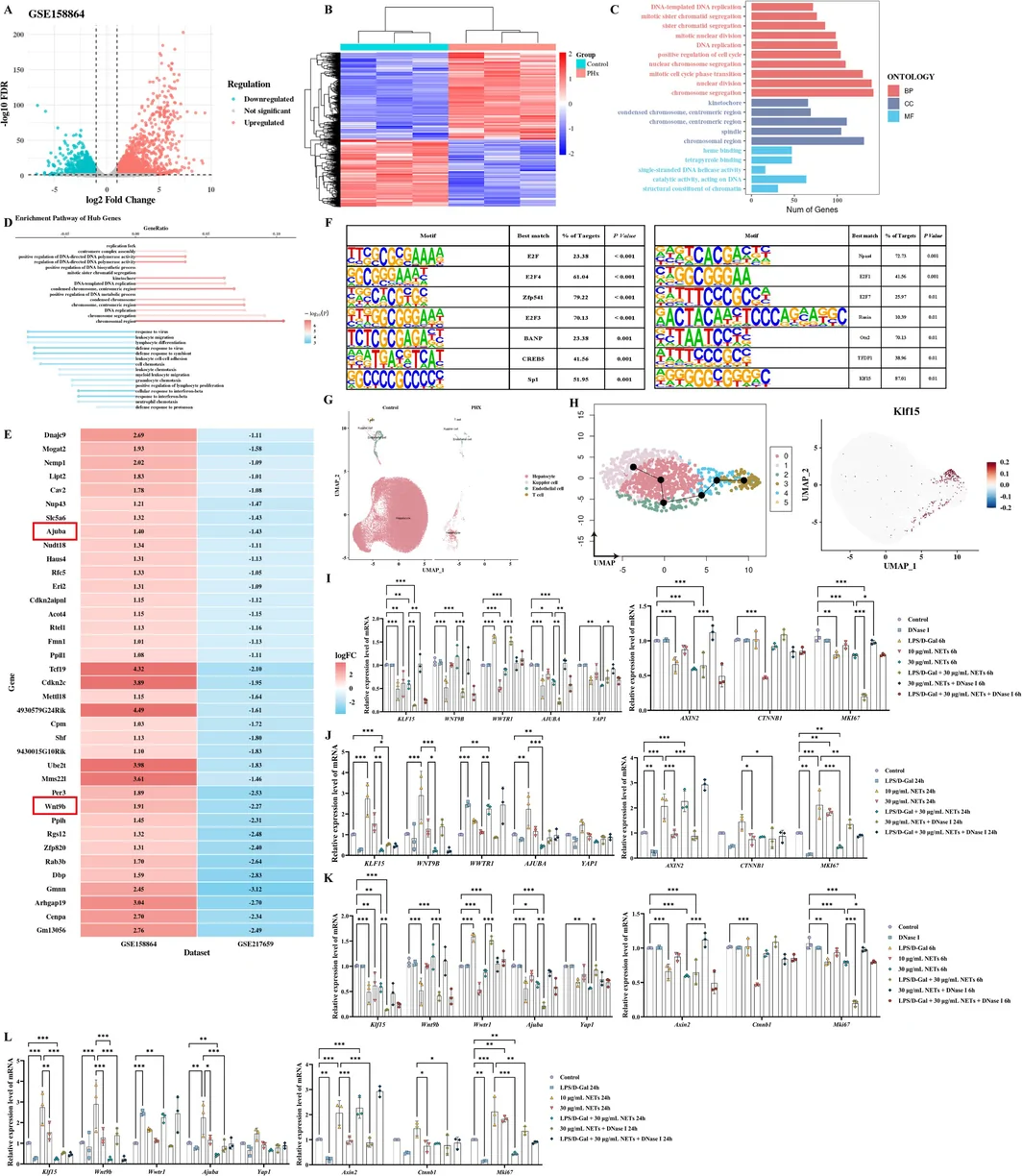
KLF15 acts as a pivotal regulator in liver regeneration. (A, B) Volcano plot (left) and heatmap (right) illustrating DEGs identified from RNA‐seq of liver tissues from the control and PHx mice (*n* = 3). (C) GO enrichment analysis illustrating the biological pathways enriched in the liver tissues of PHx group. (D) KEGG enrichment analysis of the DEGs exhibiting discordant expression patterns between the PHx and ALF groups (*n* = 3). (E) Heatmap displaying the intersection of genes that are significantly upregulated in PHx yet downregulated in ALF (*n* = 3). (F) Top transcription factor binding motifs predicted by HOMER analysis within the promoter regions of the identified discordant genes. (G) UMAP plot visualisation identifying distinct cell clusters in the scRNA‐seq dataset of murine liver tissues. (H) Pseudotime trajectory analysis of the hepatocyte cluster overlaying the expression dynamics of KLF15. (I) qRT‐PCR analysis of *KLF15*, *WNT9B*, *WWTR1*, *AJUBA*, *YAP1*, *AXIN2*, *CTNNB1*, and *MKI67* mRNA levels in HepG2 cells from the control, DNase I, LPS/D‐Gal 6 h, 10 μg/mL NETs 6 h, 30 μg/mL NETs 6 h, LPS/D‐Gal + 30 μg/mL NETs 6 h, 30 μg/mL NETs + DNase I 6 h, and LPS/D‐Gal + 30 μg/mL NETs + DNase I 6 h groups (*n* = 3). (J) qRT‐PCR analysis of *KLF15*, *WNT9B*, *WWTR1*, *AJUBA*, *YAP1*, *AXIN2*, *CTNNB1*, and *MKI67* mRNA levels in HepG2 cells from the control, LPS/D‐Gal 24 h, 10 μg/mL NETs 24 h, 30 μg/mL NETs 24 h, LPS/D‐Gal + 30 μg/mL NETs 24 h, 30 μg/mL NETs + DNase I 24 h, and LPS/D‐Gal + 30 μg/mL NETs + DNase I 24 h groups (*n* = 3). (K) qRT‐PCR analysis of *Klf15*, *Wnt9b*, *Wwtr1*, *Ajuba*, *Yap1*, *Axin2*, *Ctnnb1*, and *Mki67* mRNA levels in AML12 cells from the control, DNase I, LPS/D‐Gal 6 h, 10 μg/mL NETs 6 h, 30 μg/mL NETs 6 h, LPS/D‐Gal + 30 μg/mL NETs 6 h, 30 μg/mL NETs + DNase I 6 h, and LPS/D‐Gal + 30 μg/mL NETs + DNase I 6 h groups (*n* = 3). (L) qRT‐PCR analysis of *Klf15*, *Wnt9b*, *Wwtr1*, *Ajuba*, *Yap1*, *Axin2*, *Ctnnb1*, and *Mki67* mRNA levels in AML12 cells from the control, LPS/D‐Gal 24 h, 10 μg/mL NETs 24 h, 30 μg/mL NETs 24 h, LPS/D‐Gal + 30 μg/mL NETs 24 h, 30 μg/mL NETs + DNase I 24 h, and LPS/D‐Gal + 30 μg/mL NETs + DNase I 24 h groups (*n* = 3).

AJUBA is a member of the LIM domain protein family that functions by forming multi‐protein complexes within the cytoplasm [58]. Previous studies have established that AJUBA promotes cell proliferation by inhibiting LATS/Warts‐mediated phosphorylation of YAP1, thereby facilitating YAP1 nuclear translocation [59]. WNT9B, which belongs to the Wnt ligand family and is highly expressed in endothelial cells, is critically associated with the initiation of hepatocyte regeneration following PHx [60]. To further investigate the associations among NETosis, KLF15, WNT9B, and AJUBA in liver regeneration, we treated hepatocytes with LPS/D‐Gal, NETs, or a combination of both at different time points (6 and 24 h). qRT‐PCR results indicated that LPS/D‐Gal treatment consistently suppressed the expression of *KLF15*, *AJUBA*, and *MKI67* across different time points. Notably, NETs treatment exerted a bidirectional regulatory effect on hepatic regeneration depending on the concentration and duration: treatment with 10 μg/mL NETs for 24 h upregulated *KLF15*, *AJUBA*, and *MKI67*, whereas 30 μg/mL NETs for 6 h downregulated their expression. This effect was efficiently reversed by degrading NETs with DNase I. Furthermore, the co‐treatment of LPS/D‐Gal and NETs significantly suppressed *KLF15*, *AJUBA*, and *MKI67* expression regardless of the NETs treatment duration (6 or 24 h) (Figure 7I–L). Taken together, KLF15 exerts a pivotal regulatory role in liver regeneration, while NETs exhibit a bidirectional modulatory effect on this regenerative process.

### 3.7 NETs Impede Liver Regeneration by Suppressing the KLF15/AJUBA Pathway

To definitively delineate the downstream targets of KLF15, we performed molecular docking simulations for KLF15 with AJUBA and WNT9B using the HDOCK server. The simulated docking scores indicated a stronger binding affinity between KLF15 and AJUBA (Figure 8A). Subsequent Co‐IP assays explicitly confirmed the direct physical interaction between KLF15 and AJUBA (Figure 8B). In HepG2 and AML12 cells, treatment with LPS/D‐Gal or NETs reduced the protein levels of KLF15, AJUBA, and MKI67, which were further profoundly diminished in the LPS/D‐Gal + NETs group. Conversely, the protein levels of p‐YAP1 exhibited an opposing trend. Degradation of NETs DNA using DNase I successfully rescued the protein levels of KLF15, AJUBA, and MKI67, while concurrently reducing p‐YAP1 expression (Figure 8C,D). IF staining revealed that the spatial co‐localization between KLF15 and AJUBA was attenuated in the LPS/D‐Gal and NETs groups, reaching its lowest level in the LPS/D‐Gal + NETs group, but was effectively restored by DNase I treatment (Figure 8E,F). EdU incorporation assays correspondingly demonstrated that hepatocyte proliferation was impaired by LPS/D‐Gal and NETs, was most severely hampered in the LPS/D‐Gal + NETs group, and was rescued upon DNase I treatment (Figure 8G,H).

**FIGURE 8:**
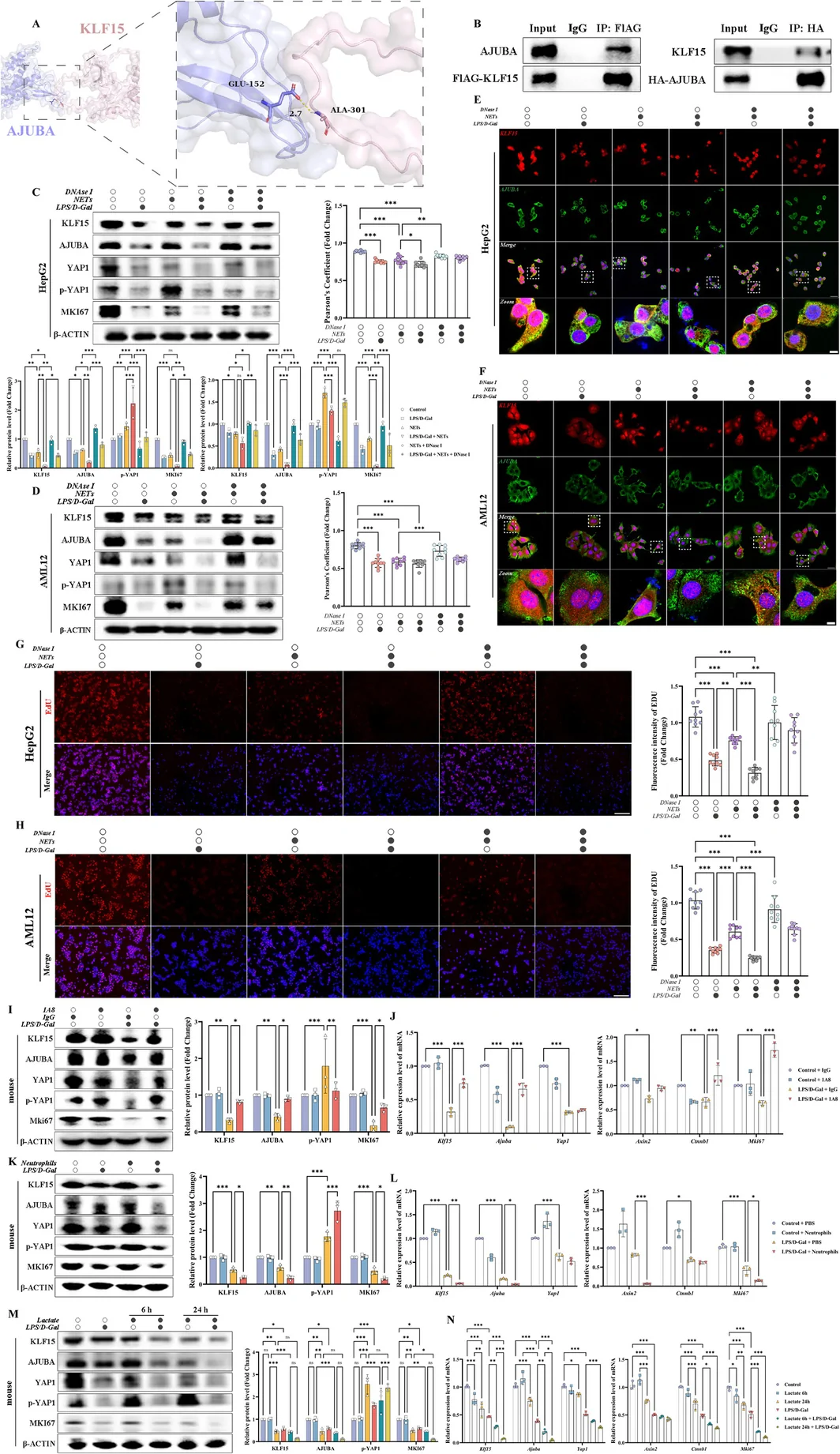
NETs DNA disrupt the KLF15/AJUBA axis to arrest hepatocyte proliferation. (A) Molecular docking analysis predicting the binding interface between KLF15 and AJUBA. (B) Co‐IP assay confirming the physical interaction between KLF15 and AJUBA (*n* = 3). (C) WB analysis of KLF15, AJUBA, YAP1, p‐YAP1, and MKI67 protein levels in HepG2 cells from the control, LPS/D‐Gal, NETs, LPS/D‐Gal + NETs, NETs + DNase I, and LPS/D‐Gal + NETs + DNase I groups (*n* = 3). (D) WB analysis of KLF15, AJUBA, YAP1, p‐YAP1, and MKI67 protein levels in AML12 cells from the control, LPS/D‐Gal, NETs, LPS/D‐Gal + NETs, NETs + DNase I, and LPS/D‐Gal + NETs + DNase I groups (*n* = 3). (E) IF staining of KLF15, AJUBA, and DAPI in HepG2 cells from the control, LPS/D‐Gal, NETs, LPS/D‐Gal + NETs, NETs + DNase I, and LPS/D‐Gal + NETs + DNase I groups (*n* = 9). Scale bar = 10 μm. (F) IF staining of KLF15, AJUBA, and DAPI in AML12 cells from the control, LPS/D‐Gal, NETs, LPS/D‐Gal + NETs, NETs + DNase I, and LPS/D‐Gal + NETs + DNase I groups (*n* = 9). Scale bar = 10 μm. (G) EdU incorporation assay in HepG2 cells from the control, LPS/D‐Gal, NETs, LPS/D‐Gal + NETs, NETs + DNase I, and LPS/D‐Gal + NETs + DNase I groups (*n* = 9). Scale bar = 50 μm. (H) EdU incorporation assay in AML12 cells from the control, LPS/D‐Gal, NETs, LPS/D‐Gal + NETs, NETs + DNase I, and LPS/D‐Gal + NETs + DNase I groups (*n* = 9). Scale bar = 50 μm. (I) WB analysis of KLF15, AJUBA, YAP1, p‐YAP1, and MKI67 protein levels in liver tissues from the control + IgG, LPS/D‐Gal + IgG, control + 1A8, and LPS/D‐Gal + 1A8 groups (*n* = 3). (J) qRT‐PCR analysis of *Klf15*, *Ajuba*, *Yap1*, *Axin2*, *Ctnnb1*, and *Mki67* mRNA levels in the control + IgG, LPS/D‐Gal + IgG, control + 1A8, and LPS/D‐Gal + 1A8 groups (*n* = 3). (K) WB analysis of KLF15, AJUBA, YAP1, p‐YAP1, and MKI67 protein levels in the control + PBS, LPS/D‐Gal + PBS, control + Neutrophils, and LPS/D‐Gal + Neutrophils groups (*n* = 3). (L) qRT‐PCR analysis of *Klf15*, *Ajuba*, *Yap1*, *Axin2*, *Ctnnb1*, and *Mki67* mRNA levels in the control + PBS, LPS/D‐Gal + PBS, control + Neutrophils, and LPS/D‐Gal + Neutrophils groups (*n* = 3). (M) WB analysis of KLF15, AJUBA, YAP1, p‐YAP1, and MKI67 protein levels in the control + PBS, LPS/D‐Gal + PBS, Lactate 6 h + PBS, Lactate 6 h + LPS/D‐Gal, Lactate 24 h + PBS, and Lactate 24 h + LPS/D‐Gal groups (*n* = 3). (N) qRT‐PCR analysis of *Klf15*, *Ajuba*, *Yap1*, *Axin2*, *Ctnnb1*, and *Mki67* mRNA levels in the control + PBS, LPS/D‐Gal + PBS, Lactate 6 h + PBS, Lactate 6 h + LPS/D‐Gal, Lactate 24 h + PBS, and Lactate 24 h + LPS/D‐Gal groups (*n* = 3). (White dots ‘○’ represent the untreated groups, while black dots ‘●’ denote the groups receiving the indicated treatment.).

In vivo, compared to the LPS/D‐Gal + IgG group, neutrophil depletion (LPS/D‐Gal + 1A8) successfully restored the protein and mRNA levels of KLF15, AJUBA, and MKI67, reduced the expression of p‐YAP1 (Figure 8I,J). Conversely, adoptive transfer of neutrophils (LPS/D‐Gal + Neutrophils) further exacerbated the suppression of KLF15, AJUBA, and MKI67, elevated p‐YAP1 levels compared to the LPS/D‐Gal + PBS group (Figure 8K,L). Moreover, in the lactate‐induced models, the Lactate 24 h + LPS/D‐Gal group exhibited the most profound suppression of KLF15, AJUBA, and MKI67 expression, with the Lactate 6 h + LPS/D‐Gal group also showing a reduction compared to the LPS/D‐Gal group; p‐YAP1 protein levels displayed a consistently inverse trend (Figure 8M,N). Collectively, these findings validate that NETs suppress liver regeneration by disrupting the KLF15/AJUBA axis.

### 3.8 KLF15 Overexpression Promotes Liver Regeneration and Reverses NETs‐Induced Regenerative Inhibition

To further validate the critical regulatory role of the KLF15/AJUBA pathway in liver regeneration, we established KLF15 OE and AJUBA OE HepG2 cells. Compared to the CMV control group, KLF15 OE upregulated the expression of AJUBA and MKI67 while downregulating p‐YAP1, thereby promoting liver regeneration (Figure 9A,B). Conversely, AJUBA OE did not alter KLF15 expression levels but successfully upregulated MKI67 and downregulated p‐YAP1 to promote regeneration (Figure S2B,C), indicating that AJUBA functions downstream of KLF15. Furthermore, WB and qRT‐PCR analyses revealed that while AJUBA Sh did not alter KLF15 expression levels, it led to an increase in p‐YAP1 and a marked reduction in MKI67, ultimately inhibiting liver regeneration (Figure 9E,F). Similarly, in the AML12 cells, Klf15 OE upregulated AJUBA and MKI67, and downregulated p‐YAP1 to foster regeneration (Figure 9C,D). In contrast, Ajuba Sh did not affect KLF15 levels but upregulated p‐YAP1, accompanied by the downregulation of MKI67, thereby impeding liver regeneration (Figure 9G,H). EdU incorporation and scratch wound‐healing assays further demonstrated that KLF15 OE and AJUBA OE promoted cellular proliferation and repair, whereas AJUBA Sh significantly suppressed these regenerative processes (Figure 9I,J, Figure S2D–F).

**FIGURE 9:**
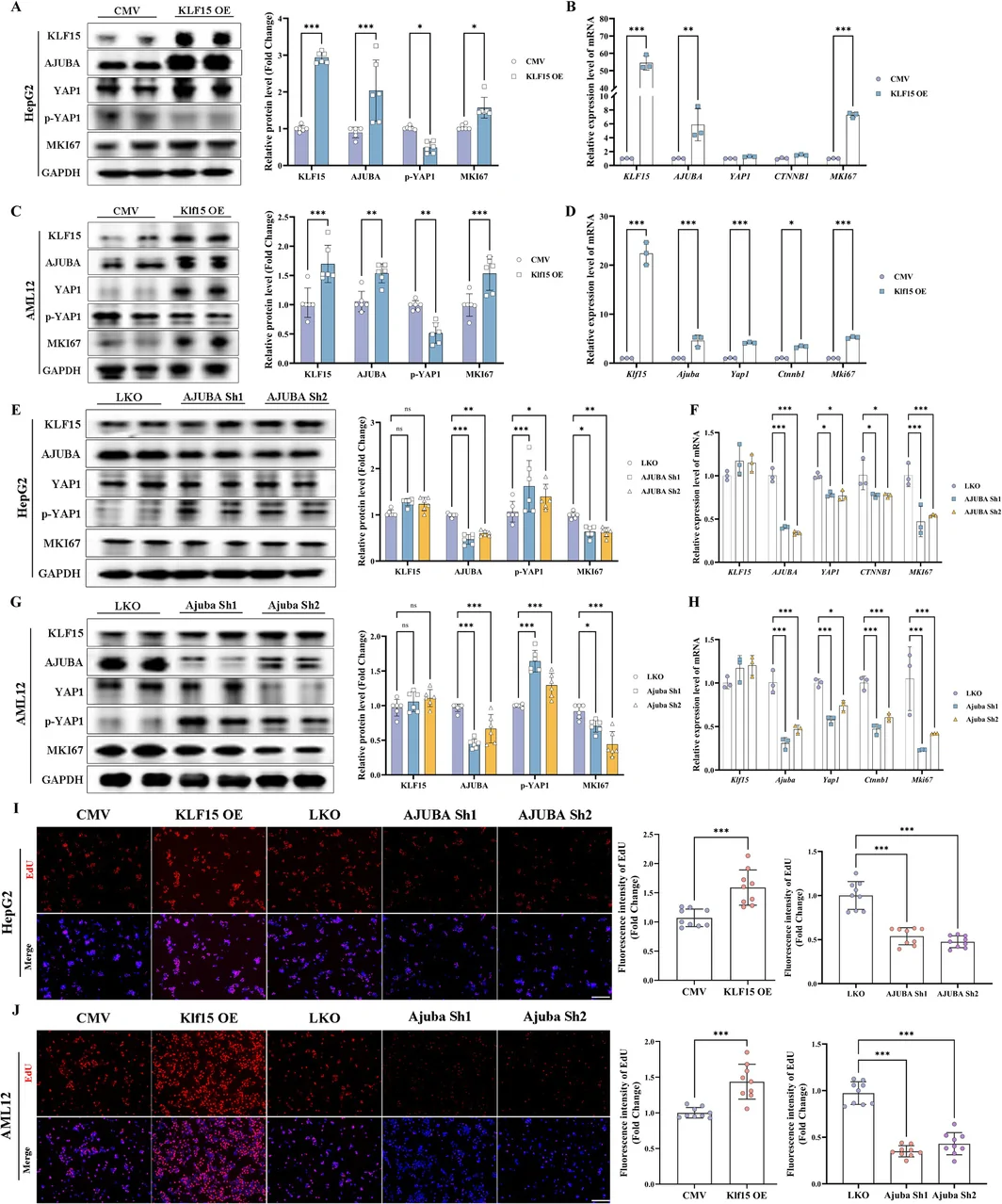
KLF15 overexpression promotes while AJUBA knockdown inhibits hepatocyte proliferation. (A) WB analysis of KLF15, AJUBA, YAP1, p‐YAP1, and MKI67 protein levels in HepG2 cells from the CMV and KLF15 OE groups (*n* = 6). (B) qRT‐PCR analysis of *KLF15*, *AJUBA*, *YAP1*, and *MKI67* mRNA levels in HepG2 cells from the CMV and KLF15 OE groups (*n* = 3). (C) WB analysis of KLF15, AJUBA, YAP1, p‐YAP1, and MKI67 protein levels in AML12 cells from the CMV and Klf15 OE groups (*n* = 6). (D) qRT‐PCR analysis of *Klf15*, *Ajuba*, *Yap1*, and *Mki67* mRNA levels in AML12 cells from the CMV and Klf15 OE groups (*n* = 3). (E) WB analysis of KLF15, AJUBA, YAP1, p‐YAP1, and MKI67 protein levels in HepG2 cells from the LKO and AJUBA Sh groups (*n* = 6). (F) qRT‐PCR analysis of *KLF15*, *AJUBA*, *YAP1*, and *MKI67* mRNA levels in HepG2 cells from the LKO and AJUBA Sh groups (*n* = 3). (G) WB analysis of KLF15, AJUBA, YAP1, p‐YAP1, and MKI67 protein levels in AML12 cells from the LKO, and Ajuba Sh groups (*n* = 6). (H) qRT‐PCR analysis of *Klf15*, *Ajuba*, *Yap1*, and *Mki67* mRNA levels in AML12 cells from the LKO, and Ajuba Sh groups (*n* = 3). (I) EdU incorporation assay in HepG2 cells from the CMV, KLF15 OE, LKO, and AJUBA Sh groups (*n* = 9). Scale bar = 50 μm. (J) EdU incorporation assay in AML12 cells from the CMV, Klf15 OE, LKO, and Ajuba Sh groups (*n* = 9). Scale bar = 50 μm.

To definitively verify the pivotal role of KLF15 in sustaining liver regeneration, we exposed KLF15 OE HepG2 and AML12 cells to NETs. We found that in both the KLF15 OE and AJUBA OE groups, the expression levels of AJUBA and MKI67 were significantly elevated compared to the CMV control group, while p‐YAP1 levels were decreased. Notably, these regenerative effects occurred without altering the mRNA levels of upstream TLR9 or MYD88 (Figure 10A–F, Figure S2G,H). EdU staining assays consistently indicated that compared to the CMV group cells subjected to LPS/D‐Gal, NETs, or LPS/D‐Gal + NETs treatment, KLF15 OE significantly promoted cell proliferation under the corresponding stress conditions (Figure 10G,H). Taken together, KLF15 OE upregulates AJUBA and effectively rescues the hepatic regenerative capacity that is otherwise impaired by NETs, thereby facilitating the repair of acute liver injury.

**FIGURE 10:**
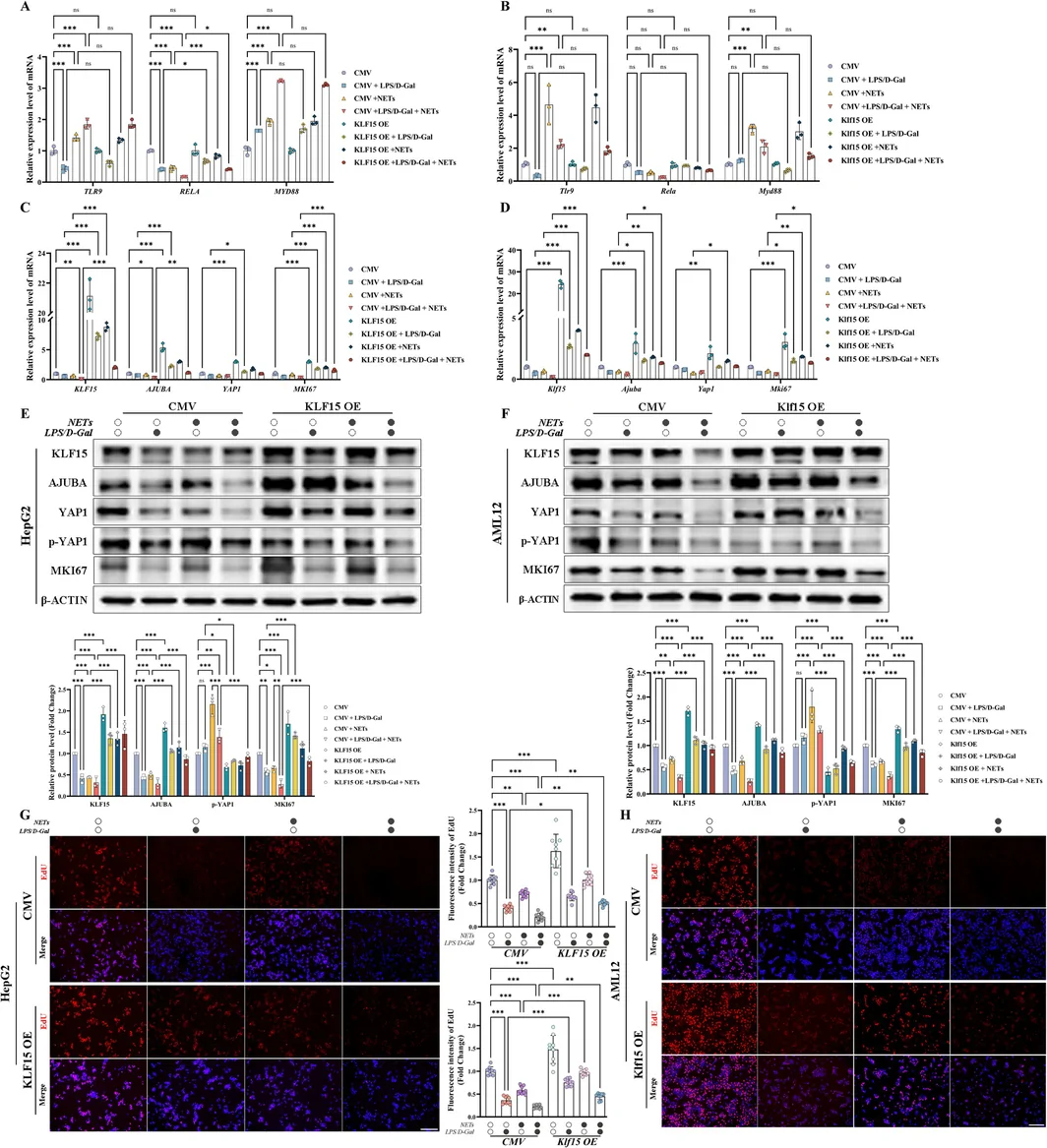
KLF15 overexpression rescues NETs‐induced regenerative inhibition. (A) qRT‐PCR analysis of *TLR9*, *RELA*, and *MYD88* mRNA levels in HepG2 cells from the CMV, CMV + LPS/D‐Gal, CMV + NETs, CMV + LPS/D‐Gal + NETs, KLF15 OE, KLF15 OE + LPS/D‐Gal, KLF15 OE + NETs, and KLF15 OE + LPS/D‐Gal + NETs groups (*n* = 3). (B) qRT‐PCR analysis of *Tlr9*, *Rela*, and *Myd88* mRNA levels in AML12 cells from the CMV, CMV + LPS/D‐Gal, CMV + NETs, CMV + LPS/D‐Gal + NETs, Klf15 OE, Klf15 OE + LPS/D‐Gal, Klf15 OE + NETs, and Klf15 OE + LPS/D‐Gal + NETs groups (*n* = 3). (C) qRT‐PCR analysis of *KLF15*, *AJUBA*, *YAP1*, and *MKI67* mRNA levels in HepG2 cells from the CMV, CMV + LPS/D‐Gal, CMV + NETs, CMV + LPS/D‐Gal + NETs, KLF15 OE, KLF15 OE + LPS/D‐Gal, KLF15 OE + NETs, and KLF15 OE + LPS/D‐Gal + NETs groups (*n* = 3). (D) qRT‐PCR analysis of *Klf15*, *Ajuba*, *Yap1*, and *Mki67* mRNA levels in AML12 cells from the CMV, CMV + LPS/D‐Gal, CMV + NETs, CMV + LPS/D‐Gal + NETs, Klf15 OE, Klf15 OE + LPS/D‐Gal, Klf15 OE + NETs, and Klf15 OE + LPS/D‐Gal + NETs groups (*n* = 3). (E) WB analysis of KLF15, AJUBA, YAP1, p‐YAP1, and MKI67 protein levels in HepG2 cells from the CMV, CMV + LPS/D‐Gal, CMV + NETs, CMV + LPS/D‐Gal + NETs, KLF15 OE, KLF15 OE + LPS/D‐Gal, KLF15 OE + NETs, and KLF15 OE + LPS/D‐Gal + NETs groups (*n* = 3). (F) WB analysis of KLF15, AJUBA, YAP1, p‐YAP1, and MKI67 protein levels in AML12 cells from the CMV, CMV + LPS/D‐Gal, CMV + NETs, CMV + LPS/D‐Gal + NETs, Klf15 OE, Klf15 OE + LPS/D‐Gal, Klf15 OE + NETs, and Klf15 OE + LPS/D‐Gal + NETs groups (*n* = 3). (G) EdU incorporation assay in HepG2 cells from the CMV, CMV + LPS/D‐Gal, CMV + NETs, CMV + LPS/D‐Gal + NETs, KLF15 OE, KLF15 OE + LPS/D‐Gal, KLF15 OE + NETs, and KLF15 OE + LPS/D‐Gal + NETs groups (*n* = 9). Scale bar = 50 μm. (H) EdU incorporation assay in AML12 cells from the CMV, CMV + LPS/D‐Gal, CMV + NETs, CMV + LPS/D‐Gal + NETs, Klf15 OE, Klf15 OE + LPS/D‐Gal, Klf15 OE + NETs, and Klf15 OE + LPS/D‐Gal + NETs groups (*n* = 9). Scale bar = 50 μm. (White dots ‘○’ represent the untreated groups, while black dots ‘●’ denote the groups receiving the indicated treatment.)

Moreover, to validate the clinical relevance of our findings, we evaluated the bulk transcriptomic profiles of liver tissues derived from patients with APAP‐induced ALF, HBV‐related ALF, and HBV‐associated ACLF (Figure S3A–D). By assessing the key target genes identified in our study, we observed a profound downregulation of *KLF15* and *AJUBA* across the cohorts, which was particularly pronounced in patients with HBV‐ALF. Consistent with this, *YAP1* was also significantly downregulated in HBV‐ALF, further corroborating the clinical involvement of the KLF15/AJUBA signalling axis. Notably, the expression of the proliferation marker *MKI67* exhibited heterogeneity; it was robustly upregulated in both ALF cohorts. This suggests a compensatory activation of hepatic regeneration following severe liver injury, potentially reflecting differences in the magnitude of injury between clinical patients and murine models. Regarding glycolytic metabolism, *HK2* was significantly upregulated in HBV‐ALF and HBV‐ACLF, accompanied by an elevation in *SLC16A3* (*MCT4*) expression. This indicates enhanced glycolysis and increased lactate efflux during intense inflammatory activation. Interestingly, *LDHB* expression was consistently upregulated across all three types of liver failure, which may represent a compensatory mechanism by the surviving liver parenchyma to clear excessive systemic lactate. Collectively, these clinical transcriptomic signatures align with our experimental models, underscoring that NETs‐induced impairment of the KLF15/AJUBA axis is a critical pathological event during liver failure.

## 4 Discussion

ALF is a severe, life‐threatening clinical syndrome. Despite presenting distinct characteristics across various stages of its onset and progression, immunomodulation remains a highly promising therapeutic strategy [61]. In the present study, we demonstrate that the activation of KCs drives the generation of lactate via enhanced glycolysis, thereby orchestrating a cascading response of neutrophil recruitment and activation, ultimately leading to the robust release of NETs. The DNA derived from these NETs is subsequently recognised by hepatocytes via the TLR9 signalling pathway, which in turn modulates the downstream KLF15/AJUBA/YAP1 signalling axis to dictate the precise balance between liver regeneration and hepatic injury.

Historically regarded merely as a metabolic byproduct, lactate has recently garnered increasing attention for its critical roles in immunomodulation and epigenetic modification [62]. Our findings demonstrate that neutrophils can internalise exogenous lactate into mitochondria through the upregulation of MCT1, thereby triggering NETosis. This process was effectively blunted by the MCT1 inhibitor AZD3965. A recent study reported a parallel phenomenon wherein neutrophil mitochondria sense *Staphylococcus*‐derived lactate to activate NETosis, specifically characterised by the phagocytosis of lactate‐producing *Staphylococcus aureus* accompanied by MCT1 expression, thus underscoring the potent activating effect of bacteria‐derived lactate on NETosis [49]. Furthermore, our findings indicate that NETosis triggered by mitochondrial‐internalised lactate is closely associated with the cytosolic leakage of mtDNA, as well as the accumulation and release of mtROS. Consistent with this, a study on Sjögren's syndrome reported that lactate induces mtDNA damage and cytosolic leakage in acinar epithelial‐like cells, ultimately activating the cGAS‐STING pathway [55]. Moreover, it has been documented that lactate can fuel the electron transport chain, promoting mitochondrial oxidative phosphorylation and ATP production—a process inevitably accompanied by ROS generation, which is indispensable for the initiation of NETosis [63, 64]. Notably, the ectopic release of mtDNA not only reflects compromised mitochondrial integrity but also acts as a robust inflammatory mediator. Upon recognition by the host neutrophil or adjacent cells, it potently amplifies the NETosis cascade [65]. Moreover, the overproduction of mtROS drives mtDNA leakage and concurrently oxidizes the DNA, thereby significantly enhancing its pro‐inflammatory immunogenicity [54]. In addition, while our study observed that 10 mM lactate treatment promoted histone lactylation, the global changes in histone lactylation levels did not parallel the induction of NETosis, suggesting that global histone lactylation is not the primary driver of NETosis in our model. However, a study on myocardial ischemia–reperfusion injury reported that lactate triggers NETosis by promoting the histone lactylation‐mediated transcriptional activation of *MICU3*. Although that study observed activation at 5 mM lactate, it did not evaluate global histone lactylation levels across a lactate gradient [66]. Nevertheless, our study has yet to fully elucidate the precise molecular targets and downstream signalling pathways driving this process, which warrants further investigation.

In our in vivo models, independent lactate stimulation for 24 h augmented neutrophil infiltration and NETosis levels without inducing overt hepatic morphological disruption. However, the co‐administration of lactate with LPS/D‐Gal provoked substantially more severe NETosis and profound liver injury. These data suggest that the synergistic alteration of the metabolic and immune microenvironments is pivotal for amplifying the inflammatory cascade and exacerbating hepatic damage. However, we must acknowledge the physiological limitations of the systemic lactate administration model used in this study. Hepatic sinusoids exhibit distinct functional zonation characterised by heterogeneous cellular compositions, which establish complex gradients of metabolic substrates, products, hormones, and oxygen [67]. While intraperitoneal lactate injection effectively demonstrates the pro‐inflammatory and NETosis‐inducing potential of lactate overload, it may not fully recapitulate the intricate, localized paracrine concentration gradients that naturally evolve within the hepatic sinusoids during acute liver injury.

As the predominant liver‐resident innate immune cells, KCs are robustly activated during ALF, releasing an array of cytokines and metabolites into the hepatic microenvironment to ignite downstream immune cell cascades [68]. Through scRNA‐seq analysis, we observed that the transcriptional reprogramming of key glycolysis‐related genes during ALF was distinctly concentrated within KCs, which exhibited a significant interactome correlation with neutrophils. In vitro, LPS stimulation upregulated the expression of glycolysis‐associated genes and the lactate efflux transporter MCT4 in macrophages, consequently propelling the robust release of lactate into the culture supernatant. In vivo, macrophage‐depleted mice exhibited an amelioration of liver injury. Notably, this macrophage depletion was accompanied by a concomitant precipitous decline in hepatic lactate levels, neutrophil infiltration, and the extent of NETosis, strongly indicating a tightly coupled cascade linking KCs activation, lactate accumulation, and NETosis. Although our study did not encompass comprehensive profiling of the cytokine milieu—which to some extent limits our ability to definitively exclude the contributory roles of classical pro‐inflammatory cytokines [69] (e.g., TNF‐α, IL‐1β) in neutrophil activation—our lactate and lactate plus LPS/D‐Gal treatment models collectively substantiate the direct action of lactate on neutrophil recruitment and NETosis. This unequivocally demonstrates that lactate is not merely a passive byproduct of inflammation, but rather a potent ‘second signal’ capable of independently modulating or significantly amplifying the neutrophil‐mediated immune response.

NETs are macromolecular DNA‐protein complexes. Our in vitro experiments demonstrated that NETs DNA initially adheres to hepatocytes and, with prolonged incubation, is subsequently internalised, as evidenced by diminished peri‐membrane DNA fluorescence. Consistently, this internalisation is accompanied by the upregulation of TLR9 expression, as well as elevated levels of p‐NFκB p65 and MYD88, indicating that the internalisation of NETs DNA and subsequent signal activation are predominantly mediated by the TLR9 pathway. In our in vivo models, neutrophil depletion under LPS/D‐Gal treatment resulted in the clearance of NETosis, suppression of the TLR9 signalling cascade, and marked amelioration of liver injury. Conversely, the transfer of bone marrow‐derived neutrophils further exacerbated TLR9 pathway activation and liver injury upon LPS/D‐Gal challenge. Although cGAS serves as a canonical cytosolic DNA sensor, TLR9 exhibited a more pronounced relevance within the specific context of our study. Functionally, TLR9 can sense both pathogen‐derived and endogenous host DNA. Notably, TLR9 deficiency in ALD has been shown to exacerbate oxidative stress and mitochondrial dysfunction [70]. Interestingly, our in vitro data revealed that direct LPS/D‐Gal exposure downregulated basal TLR9 levels in HepG2 and AML12 cells, likely due to the D‐Gal‐induced blockade of de novo protein synthesis [71]. In stark contrast, exposure to NETs dramatically upregulated the TLR9 signalling axis. This dynamic divergence is further supported by expression trends in clinical transcriptomic datasets, where the TLR9 signalling cascade exhibits a general downward trend in toxin‐induced APAP‐ALF, but an overall upward pattern in immune‐driven HBV‐ACLF. Together, these findings reinforce the concept that the hepatic TLR9 cascade is primarily triggered by immune‐derived DAMPs (such as NETs and mtDNA) during intense sterile inflammation, rather than by direct chemical hepatotoxicity. Nevertheless, future studies utilising TLR9‐specific inhibitors or knockout cell lines are essential to definitively elucidate its indispensable role in the recognition of NETs DNA.

KLF15 is a pivotal transcriptional regulator in maintaining hepatic metabolic homeostasis [31]. In our study, we identified KLF15 as a core regeneration‐associated candidate factor via HOMER motif prediction, based on its pronounced upregulation in PHx mouse livers and concomitant low expression in ALF. While previous studies have highlighted the critical role of KLF15 in glucose and lipid metabolism and its hepatoprotective effects during alcoholic liver injury, we further identified WNT9B and AJUBA as its potential downstream targets. Subsequent qRT‐PCR, molecular docking simulations and Co‐IP assays revealed a robust functional association with AJUBA. Our in vitro assays revealed that NETs exerted pro‐regenerative properties by upregulating KLF15 and AJUBA at low concentrations (10 μg/mL) or with prolonged stimulation (24 h). Conversely, high‐concentration (30 μg/mL) or short‐term (6 h) NETs stimulation downregulated KLF15 and AJUBA, thereby impeding liver regeneration. Notably, co‐treatment with LPS/D‐Gal and NETs robustly downregulated KLF15 and AJUBA to suppress liver regeneration, irrespective of NETs treatment duration. This phenomenon posits the existence of a critical ‘inflammation‐regeneration homeostatic threshold’ in the liver: moderate NETs signalling may act as DAMPs to initiate early reparative mechanisms. However, once the inflammatory burden exceeds this threshold, excessive TLR9‐mediated inflammatory signalling downregulates KLF15. This downregulation aberrantly reactivates the Hippo pathway (by alleviating AJUBA‐mediated suppression of LATS), ultimately precipitating hepatocyte cell cycle arrest. Furthermore, KLF15 OE hepatocytes upregulated AJUBA and MKI67 without altering the TLR9 signalling pathway, whereas AJUBA knockdown upregulated p‐YAP1 and suppressed hepatocyte proliferation without affecting KLF15 levels. Thus, the KLF15/AJUBA/YAP1 cascade exerts a profound regulatory role on liver regeneration. Collectively, these data indicate that KLF15 regulates AJUBA through both transcriptional activation and direct protein–protein interactions. However, it is important to acknowledge that while our current data strongly support this dual regulation, definitive evidence for direct promoter binding—such as through chromatin immunoprecipitation or dual‐luciferase reporter assays—remains to be established. Further investigations are warranted to precisely dissect the relative contributions of transcriptional activation versus post‐translational interactions in the context of ALF.

## 5 Conclusion

In summary, our study elucidates a tightly orchestrated regulatory mechanism linking metabolic, immune, and regenerative signalling cascades, which is mutually driven by KCs, neutrophils, and hepatocytes. This crosstalk provides a novel perspective on how the inflammatory and metabolic microenvironments dictate hepatic tissue regeneration during ALF. Consequently, targeting any pivotal node within this integrated cascade holds substantial promise as a potential therapeutic strategy for patients with ALF.

## Author Contributions

**Jin Guo**, **Xiaoya Zhang:** conceptualization, writing – original draft, writing – review and editing, methodology. **Danmei Zhang:** methodology, investigation, writing – review and editing. **Chunxia Shi:** methodology, investigation, conceptualization, writing – review and editing. **Luwen Wang:** supervision, writing – review and editing. **Zuojiong Gong:** project administration, supervision, writing – review and editing.

## Funding

This article was funded by the National Natural Science Foundation of China (grant no. 82270627).

## Conflicts of Interest

The authors declare no conflicts of interest.

## Supporting information


**Figure S1:** Validation of neutrophil purity and metabolic profiling of ALF scRNA‐seq data. (A) Chemical structure of Cy3‐labelled lactate. (B) Flow cytometry analysis illustrating the percentage of Ly6g^+^CD11b^+^ cells in the isolated neutrophil population (*n* = 3). (C) UMAP plot visualisation and dot plot showing the expression of representative marker genes used for the identification of distinct cell subclusters. (D) UMAP plots demonstrating the dynamic shift in glycolysis across cell clusters between the Control and ALF groups. (E) UMAP plots illustrating the heterogeneity of the TCA cycle across cell clusters in the Control and ALF groups. (F) UMAP plots comparing the signature of glycolysis between the KC_glycolysis_pos and KC_glycolysis_neg clusters within the Control and ALF groups.

**Figure S2:** Annotation of PHx scRNA‐seq data and effects of AJUBA OE on hepatocyte proliferation. (A) UMAP plot visualisation and dot plot displaying the expression levels of representative marker genes used for cell cluster identification in the Control and PHx groups. (B) WB analysis of KLF15, AJUBA, YAP1, p‐YAP1, and MKI67 protein levels in HepG2 cells from the CMV and AJUBA OE groups (*n* = 6). (C) qRT‐PCR analysis of *KLF15*, *AJUBA*, *YAP1*, and *MKI67* mRNA levels in HepG2 cells from the CMV and AJUBA OE groups (*n* = 3). (D) EdU incorporation assay in HepG2 cells from the CMV and AJUBA OE groups (*n* = 9). Scale bar = 50 μm. (E) Wound healing assay in HepG2 cells from the CMV, KLF15 OE, LKO, and AJUBA Sh groups (*n* = 6). Scale bar = 100 μm. (F) Wound healing assay in AML12 cells from the CMV, Klf15 OE, LKO, and Ajuba Sh groups (*n* = 6). Scale bar = 100 μm. (G) qRT‐PCR analysis of *TLR9*, *RELA*, and *MYD88* mRNA levels in HepG2 cells from the CMV, CMV + LPS/D‐Gal, CMV + NETs, CMV + LPS/D‐Gal + NETs, AJUBA OE, AJUBA OE + LPS/D‐Gal, AJUBA OE + NETs, and AJUBA OE + LPS/D‐Gal + NETs groups (*n* = 3). (H) qRT‐PCR analysis of *KLF15*, *AJUBA*, *YAP1*, and *MKI6*7 mRNA levels in HepG2 cells from the CMV, CMV + LPS/D‐Gal, CMV + NETs, CMV + LPS/D‐Gal + NETs, AJUBA OE, AJUBA OE + LPS/D‐Gal, AJUBA OE + NETs, and AJUBA OE + LPS/D‐Gal + NETs groups (*n* = 3).

**Figure S3:** Transcriptomic analysis of clinical liver tissues validates the expression changes of key markers during hepatic failure. (A) Volcano plot (left) and heatmap (right) illustrating DEGs identified from transcriptome data of liver tissues from control and APAP‐induced ALF patients. (B) Volcano plot (left) and heatmap (right) illustrating DEGs identified from transcriptome data of liver tissues from control and HBV‐related ALF patients. (C) Volcano plot (left) and heatmap (right) illustrating DEGs identified from transcriptome data of liver tissues from control and HBV‐related ACLF patients. (D) Heatmap depicting the expression profiles of markers in liver tissues from the ALF and ACLF patients.

**Table S1:** The information on the used antibodies.
**Table S2:** Primer sequences for qRT‐PCR.

**Data S1:** Supplementary legends.

**Data S2:** Origin western blots.

## Data Availability

All relevant data presented in this study are included in the article and its Supporting Information files. Any other data that support the findings discussed here are available from the corresponding author upon request.

## References

[1] European Association for the Study of the Liver, Clinical Practice Guidelines Panel , J. Wendon , et al., “EASL Clinical Practical Guidelines on the Management of Acute (Fulminant) Liver Failure,” Journal of Hepatology 66 (2017): 1047–1081. 10.1016/j.jhep.2016.12.003 28417882

[2] J. P. Luyendyk , E. Morozova , and B. L. Copple , “Good Cells go Bad: Immune Dysregulation in the Transition From Acute Liver Injury to Liver Failure After Acetaminophen Overdose,” Drug Metabolism and Disposition 52 (2024): 722–728. 10.1124/dmd.123.001280 PMC11257689 38050055

[3] W. Khamri , R. D. Abeles , T. Z. Hou , et al., “Increased Expression of Cytotoxic T‐Lymphocyte‐Associated Protein 4 by T Cells, Induced by B7 in Sera, Reduces Adaptive Immunity in Patients With Acute Liver Failure,” Gastroenterology 153 (2017): 263–276.e8. 10.1053/j.gastro.2017.03.023 PMC5516432 28363639

[4] O. Krenkel , J. C. Mossanen , and F. Tacke , “Immune Mechanisms in Acetaminophen‐Induced Acute Liver Failure,” HepatoBiliary Surgery and Nutrition 3 (2014): 331–343. 10.3978/j.issn.2304-3881.2014.11.01 PMC4273118 25568858

[5] X. G. Fan , S. Y. Pei , D. Zhou , et al., “Melittin Ameliorates Inflammation in Mouse Acute Liver Failure via Inhibition of PKM2‐Mediated Warburg Effect,” Acta Pharmacologica Sinica 42 (2021): 1256–1266. 10.1038/s41401-020-00516-0 PMC8285470 32939034

[6] T. Dong , G. Hu , Z. Fan , et al., “Activation of GPR3‐β‐arrestin2‐PKM2 Pathway in Kupffer Cells Stimulates Glycolysis and Inhibits Obesity and Liver Pathogenesis,” Nature Communications 15 (2024): 807. 10.1038/s41467-024-45167-5 PMC10821868 38280848

[7] X. Li , Y. Yang , B. Zhang , et al., “Lactate Metabolism in Human Health and Disease,” Signal Transduction and Targeted Therapy 7 (2022): 305. 10.1038/s41392-022-01151-3 PMC9434547 36050306

[8] Y. Zhu , J. L. Zhang , X. J. Yan , Y. Ji , and F. F. Wang , “Exploring a New Mechanism Between Lactate and VSMC Calcification: PARP1/POLG/UCP2 Signaling Pathway and Imbalance of Mitochondrial Homeostasis,” Cell Death & Disease 14 (2023): 598. 10.1038/s41419-023-06113-3 PMC10484939 37679327

[9] W. Hong , X. Zeng , H. Wang , et al., “PGC‐1α Loss Promotes Mitochondrial Protein Lactylation in Acetaminophen‐Induced Liver Injury via the LDHB‐Lactate Axis,” Pharmacological Research 205 (2024): 107228. 10.1016/j.phrs.2024.107228 38810904

[10] J. Zhang , L. Hao , S. Li , et al., “mTOR/HIF‐1α Pathway‐Mediated Glucose Reprogramming and Macrophage Polarization by Sini Decoction Plus Ginseng Soup in ALF,” Phytomedicine 137 (2025): 156374. 10.1016/j.phymed.2025.156374 39798342

[11] A. Moles , L. Murphy , C. L. Wilson , et al., “A TLR2/S100A9/CXCL‐2 Signaling Network Is Necessary for Neutrophil Recruitment in Acute and Chronic Liver Injury in the Mouse,” Journal of Hepatology 60 (2014): 782–791. 10.1016/j.jhep.2013.12.005 PMC3960359 24333183

[12] K. Nakamura , S. Kageyama , and J. W. Kupiec‐Weglinski , “The Evolving Role of Neutrophils in Liver Transplant Ischemia‐Reperfusion Injury,” Current Transplantation Reports 6 (2019): 78–89. 10.1007/s40472-019-0230-4 PMC6786799 31602356

[13] M. Yalcinkaya , P. Fotakis , W. Liu , et al., “Cholesterol Accumulation in Macrophages Drives NETosis in Atherosclerotic Plaques via IL‐1β Secretion,” Cardiovascular Research 119 (2023): 969–981. 10.1093/cvr/cvac189 PMC10153645 36537208

[14] A. Shahzad , Y. Ni , Y. Yang , et al., “Neutrophil Extracellular Traps (NETs) in Health and Disease,” Molecular Biomedicine 6 (2025): 130. 10.1186/s43556-025-00337-9 PMC12675906 41335221

[15] E. P. Reeves , H. Lu , H. L. Jacobs , et al., “Killing Activity of Neutrophils Is Mediated Through Activation of Proteases by K+ Flux,” Nature 416 (2002): 291–297. 10.1038/416291a 11907569

[16] J. Guo , X. Zhang , D. Zhang , C. Shi , L. Wang , and Z. Gong , “Acetylation of Hint2 Mitigates Acute Liver Failure by Suppressing Neutrophil Chemotaxis and NETosis Through Maintaining Mitochondrial Calcium and Protein Homeostasis,” International Immunopharmacology 172 (2026): 116254. 10.1016/j.intimp.2026.116254 41570743

[17] V. Mutua and L. J. Gershwin , “A Review of Neutrophil Extracellular Traps (NETs) in Disease: Potential Anti‐NETs Therapeutics,” Clinical Reviews in Allergy and Immunology 61 (2021): 194–211. 10.1007/s12016-020-08804-7 PMC7395212 32740860

[18] F. Zeng , Y. Zhang , Z. H. Wang , et al., “Neutrophil Extracellular Traps Promote Acetaminophen‐Induced Acute Liver Injury in Mice via AIM2,” Acta Pharmacologica Sinica 45 (2024): 1660–1672. 10.1038/s41401-024-01239-2 PMC11272772 38589685

[19] F. A. von Meijenfeldt , R. T. Stravitz , J. Zhang , et al., “Generation of Neutrophil Extracellular Traps in Patients With Acute Liver Failure Is Associated With Poor Outcome,” Hepatology 75 (2022): 623–633. 10.1002/hep.32174 PMC9299791 34562318

[20] Y. Zhang , R. Wu , X. Zhan , et al., “Neutrophil Extracellular Traps Facilitate Liver Inflammation/Fibrosis Progression by Entering Macrophages and Triggering AIM2 Inflammasome‐Dependent Pyroptosis,” Cell Communication and Signaling: CCS 22 (2024): 556. 10.1186/s12964-024-01944-9 PMC11577833 39568027

[21] V. Mansuy‐Aubert , Q. L. Zhou , X. Xie , et al., “Imbalance Between Neutrophil Elastase and Its Inhibitor α1‐Antitrypsin in Obesity Alters Insulin Sensitivity, Inflammation, and Energy Expenditure,” Cell Metabolism 17 (2013): 534–548. 10.1016/j.cmet.2013.03.005 PMC3646573 23562077

[22] W. D. Cruz‐Pineda , O. L. Garibay‐Cerdenares , H. A. Rodríguez‐Ruíz , et al., “Changes in the Expression of Insulin Pathway, Neutrophil Elastase and Alpha 1 Antitrypsin Genes From Leukocytes of Young Individuals With Insulin Resistance,” Diabetes, Metabolic Syndrome and Obesity 15 (2022): 1865–1876. 10.2147/DMSO.S362881 PMC9215908 35757193

[23] S. Ben‐Moshe , T. Veg , R. Manco , et al., “The Spatiotemporal Program of Zonal Liver Regeneration Following Acute Injury,” Cell Stem Cell 29 (2022): 973–989.e10. 10.1016/j.stem.2022.04.008 35659879

[24] Y. Qian , J. Zhao , H. Wu , and X. Kong , “Innate Immune Regulation in Inflammation Resolution and Liver Regeneration in Drug‐Induced Liver Injury,” Archives of Toxicology 99 (2025): 115–126. 10.1007/s00204-024-03886-0 39395921

[25] M. Yan , Y. Huo , S. Yin , and H. Hu , “Mechanisms of Acetaminophen‐Induced Liver Injury and Its Implications for Therapeutic Interventions,” Redox Biology 17 (2018): 274–283. 10.1016/j.redox.2018.04.019 PMC6006912 29753208

[26] J. O. Russell and F. D. Camargo , “Hippo Signalling in the Liver: Role in Development, Regeneration and Disease,” Nature Reviews. Gastroenterology & Hepatology 19 (2022): 297–312. 10.1038/s41575-021-00571-w PMC9199961 35064256

[27] G. Zou and J. I. Park , “Wnt Signaling in Liver Regeneration, Disease, and Cancer,” Clinical and Molecular Hepatology 29 (2023): 33–50. 10.3350/cmh.2022.0058 PMC9845677 35785913

[28] Y. Chen , S. Shi , B. Li , et al., “Therapeutic Effects of Self‐Assembled Tetrahedral Framework Nucleic Acids on Liver Regeneration in Acute Liver Failure,” ACS Applied Materials & Interfaces 14 (2022): 13136–13146. 10.1021/acsami.2c02523 35285610

[29] X. Cai , J. Deng , L. Wang , et al., “Gallium‐Doped MXene Nanozymes Protect Liver Through Multi‐Death Pathway Blockade and Hepatocyte Regeneration,” Adv Sci (Weinh) 13 (2026): e09079. 10.1002/advs.202509079 PMC12970286 41487076

[30] M. Takashima , W. Ogawa , K. Hayashi , et al., “Role of KLF15 in Regulation of Hepatic Gluconeogenesis and Metformin Action,” Diabetes 59 (2010): 1608–1615. 10.2337/db09-1679 PMC2889759 20393151

[31] S. Han , J. W. Ray , P. Pathak , et al., “KLF15 Regulates Endobiotic and Xenobiotic Metabolism,” Nature Metabolism 1 (2019): 422–430. 10.1038/s42255-019-0054-7 32694878

[32] Z. Jiang , S. Z. Elsarrag , Q. Duan , et al., “KLF15 Cistromes Reveal a Hepatocyte Pathway Governing Plasma Corticosteroid Transport and Systemic Inflammation,” Science Advances 8 (2022): eabj2917. 10.1126/sciadv.abj2917 PMC8906731 35263131

[33] L. Fan , P. N. Hsieh , D. R. Sweet , and M. K. Jain , “Krüppel‐Like Factor 15: Regulator of BCAA Metabolism and Circadian Protein Rhythmicity,” Pharmacological Research 130 (2018): 123–126. 10.1016/j.phrs.2017.12.018 29288718

[34] H. Chen , L. Yang , X. F. Li , et al., “Krüppel‐Like Factor 15 Ameliorates Alcohol‐Induced Liver Injury in Mice via Regulation of the PFKFB3/AKT Axis,” Acta Pharmacologica Sinica 47 (2026): 357–369. 10.1038/s41401-025-01651-2 PMC12811621 40935894

[35] L. Chen , L. Yuan , J. Yang , Y. Pan , and H. Wang , “Identification of Key Immune‐Related Genes Associated With LPS/D‐GalN‐Induced Acute Liver Failure in Mice Based on Transcriptome Sequencing,” PeerJ 11 (2023): e15241. 10.7717/peerj.15241 PMC10166078 37168540

[36] T. Chen , S. Oh , S. Gregory , X. Shen , and A. M. Diehl , “Single‐Cell Omics Analysis Reveals Functional Diversification of Hepatocytes During Liver Regeneration,” JCI Insight 5 (2020): e141024. 10.1172/jci.insight.141024 PMC7710279 33208554

[37] O. Nissim , M. Melis , G. Diaz , et al., “Liver Regeneration Signature in Hepatitis B Virus (HBV)‐Associated Acute Liver Failure Identified by Gene Expression Profiling,” PLoS One 7 (2012): e49611. 10.1371/journal.pone.0049611 PMC3504149 23185381

[38] W. Wang , C. Dai , P. Zhu , et al., “Liver Transplant‐Facilitated CD161+Vα7.2+ MAIT Cell Recovery Demonstrates Clinical Benefits in Hepatic Failure Patients,” Nature Communications 16 (2025): 4022. 10.1038/s41467-025-59308-x PMC12041255 40301342

[39] S. Yu , S. Pei , M. Zhang , et al., “PKM2‐Mediated STAT3 Phosphorylation Promotes Acute Liver Failure via Regulating NLRP3‐Dependent Pyroptosis,” Communications Biology 7 (2024): 1694. 10.1038/s42003-024-07227-w PMC11669718 39722076

[40] S. Ben‐Moshe and S. Itzkovitz , “Spatial Heterogeneity in the Mammalian Liver,” Nature Reviews. Gastroenterology & Hepatology 16 (2019): 395–410. 10.1038/s41575-019-0134-x 30936469

[41] C. Nikopoulou , N. Kleinenkuhnen , S. Parekh , et al., “Spatial and Single‐Cell Profiling of the Metabolome, Transcriptome and Epigenome of the Aging Mouse Liver,” Nature Aging 3 (2023): 1430–1445. 10.1038/s43587-023-00513-y PMC10645594 37946043

[42] K. Street , D. Risso , R. B. Fletcher , et al., “Slingshot: Cell Lineage and Pseudotime Inference for Single‐Cell Transcriptomics,” BMC Genomics 19 (2018): 477. 10.1186/s12864-018-4772-0 PMC6007078 29914354

[43] R. Vento‐Tormo , M. Efremova , R. A. Botting , et al., “Single‐Cell Reconstruction of the Early Maternal–Fetal Interface in Humans,” Nature 563 (2018): 347–353. 10.1038/s41586-018-0698-6 PMC7612850 30429548

[44] J. Fleming , P. Magana , S. Nair , et al., “AlphaFold Protein Structure Database and 3D‐Beacons: New Data and Capabilities,” Journal of Molecular Biology 437 (2025): 168967. 10.1016/j.jmb.2025.168967 40133787

[45] Y. Yan , H. Tao , J. He , and S. Y. Huang , “The HDOCK Server for Integrated Protein‐Protein Docking,” Nature Protocols 15 (2020): 1829–1852. 10.1038/s41596-020-0312-x 32269383

[46] W. J. Quinn , J. Jiao , T. TeSlaa , et al., “Lactate Limits T Cell Proliferation via the NAD(H) Redox State,” Cell Reports 33 (2020): 108500. 10.1016/j.celrep.2020.108500 PMC7830708 33326785

[47] J. Ma , L. Tang , Y. Tan , et al., “Lithium Carbonate Revitalizes Tumor‐Reactive CD8+ T Cells by Shunting Lactic Acid Into Mitochondria,” Nature Immunology 25 (2024): 552–561. 10.1038/s41590-023-01738-0 PMC10907288 38263463

[48] Y. Yang , L. Song , L. Yu , J. Zhang , and B. Zhang , “H4K12 Lactylation Potentiates Mitochondrial Oxidative Stress via the Foxo1 Pathway in Diabetes‐Induced Cognitive Impairment,” Journal of Advanced Research 78 (2025): 391–407. 10.1016/j.jare.2025.02.020 PMC12684910 39965729

[49] A. D. Wise , E. G. TenBarge , J. d. C. Mendonça , et al., “Mitochondria Sense Bacterial Lactate and Drive Release of Neutrophil Extracellular Traps,” Cell Host & Microbe 33 (2025): 341–357.e9. 10.1016/j.chom.2025.02.003 PMC11955204 40020664

[50] W. Fan , C. Wang , K. Xu , H. Liang , and Q. Chi , “Ccl5+ Macrophages Drive Pro‐Inflammatory Responses and Neutrophil Recruitment in Sepsis‐Associated Acute Kidney Injury,” International Immunopharmacology 143 (2024): 113339. 10.1016/j.intimp.2024.113339 39418726

[51] J. Guyon , I. Fernandez‐Moncada , C. M. Larrieu , et al., “Lactate Dehydrogenases Promote Glioblastoma Growth and Invasion via a Metabolic Symbiosis,” EMBO Molecular Medicine 14 (2022): e15343. 10.15252/emmm.202115343 PMC9728051 36278433

[52] Y. Qian , A. Galan‐Cobo , I. Guijarro , et al., “MCT4‐Dependent Lactate Secretion Suppresses Antitumor Immunity in LKB1‐Deficient Lung Adenocarcinoma,” Cancer Cell 41 (2023): 1363–1380.e7. 10.1016/j.ccell.2023.05.015 PMC11161201 37327788

[53] B. Liu , Y. Peng , C. Wang , et al., “Baicalin Attenuates HIF‐1α‐Driven Histone Lactylation and Neutrophil Extracellular Trap (NET) Formation in Autoimmune Uveitis,” International Journal of Biological Macromolecules 341 (2026): 150264. 10.1016/j.ijbiomac.2026.150264 41544789

[54] C. Lood , L. P. Blanco , M. M. Purmalek , et al., “Neutrophil Extracellular Traps Enriched in Oxidized Mitochondrial DNA Are Interferogenic and Contribute to Lupus‐Like Disease,” Nature Medicine 22 (2016): 146–153. 10.1038/nm.4027 PMC4742415 26779811

[55] J. Xu , C. Chen , J. Yin , et al., “Lactate‐Induced mtDNA Accumulation Activates cGAS‐STING Signaling and the Inflammatory Response in Sjögren's Syndrome,” International Journal of Medical Sciences 20 (2023): 1256–1271. 10.7150/ijms.83801 PMC10542019 37786436

[56] J. Chen , T. Wang , X. Li , et al., “DNA of Neutrophil Extracellular Traps Promote NF‐κB‐Dependent Autoimmunity via cGAS/TLR9 in Chronic Obstructive Pulmonary Disease,” Signal Transduction and Targeted Therapy 9 (2024): 163. 10.1038/s41392-024-01881-6 PMC11180664 38880789

[57] G. K. Michalopoulos and B. Bhushan , “Liver Regeneration: Biological and Pathological Mechanisms and Implications,” Nature Reviews. Gastroenterology & Hepatology 18 (2021): 40–55. 10.1038/s41575-020-0342-4 32764740

[58] H. Jia , H. Peng , and Z. Hou , “Ajuba: An Emerging Signal Transducer in Oncogenesis,” Pharmacological Research 151 (2020): 104546. 10.1016/j.phrs.2019.104546 31740385

[59] M. Das Thakur , Y. Feng , R. Jagannathan , et al., “Ajuba LIM Proteins Are Negative Regulators of the Hippo Signaling Pathway,” Current Biology 20 (2010): 657–662. 10.1016/j.cub.2010.02.035 PMC2855397 20303269

[60] S. Hu , S. Liu , Y. Bian , et al., “Single‐Cell Spatial Transcriptomics Reveals a Dynamic Control of Metabolic Zonation and Liver Regeneration by Endothelial Cell Wnt2 and Wnt9b,” Cell Reports Medicine 3 (2022): 100754. 10.1016/j.xcrm.2022.100754 PMC9588996 36220068

[61] D. M. Alvarenga , M. S. Mattos , M. E. Lopes , et al., “Paradoxical Role of Matrix Metalloproteinases in Liver Injury and Regeneration After Sterile Acute Hepatic Failure,” Cells 7 (2018): 247. 10.3390/cells7120247 PMC6315354 30563238

[62] J. D. Rabinowitz and S. Enerbäck , “Lactate: The Ugly Duckling of Energy Metabolism,” Nature Metabolism 2 (2020): 566–571. 10.1038/s42255-020-0243-4 PMC7983055 32694798

[63] X. Cai , C. P. Ng , O. Jones , et al., “Lactate Activates the Mitochondrial Electron Transport Chain Independently of Its Metabolism,” Molecular Cell 83 (2023): 3904–3920.e7. 10.1016/j.molcel.2023.09.034 PMC10752619 37879334

[64] D. Azzouz and N. Palaniyar , “How Do ROS Induce NETosis? Oxidative DNA Damage, DNA Repair, and Chromatin Decondensation,” Biomolecules 14 (2024): 1307. 10.3390/biom14101307 PMC11505619 39456240

[65] M. Zhao , Y. Wang , L. Li , et al., “Mitochondrial ROS Promote Mitochondrial Dysfunction and Inflammation in Ischemic Acute Kidney Injury by Disrupting TFAM‐Mediated mtDNA Maintenance,” Theranostics 11 (2021): 1845–1863. 10.7150/thno.50905 PMC7778599 33408785

[66] H. Zhang , L. Liu , C. Shen , et al., “Lactate‐Induced Mitochondrial Calcium Uptake 3 Aggravates Myocardial Ischemia‐Reperfusion Injury by Promoting Neutrophil Extracellular Trap Formation,” Research 8 (2025): 0705. 10.34133/research.0705 PMC12123085 40452820

[67] T. Kietzmann , “Metabolic Zonation of the Liver: The Oxygen Gradient Revisited,” Redox Biology 11 (2017): 622–630. 10.1016/j.redox.2017.01.012 PMC5257182 28126520

[68] D. Xie and S. Ouyang , “The Role and Mechanisms of Macrophage Polarization and Hepatocyte Pyroptosis in Acute Liver Failure,” Frontiers in Immunology 14 (2023): 1279264. 10.3389/fimmu.2023.1279264 PMC10639160 37954583

[69] S. Han , B. Kim , D. Y. Hyeon , et al., “Distinctive CD39+CD9+ Lung Interstitial Macrophages Suppress IL‐23/Th17‐Mediated Neutrophilic Asthma by Inhibiting NETosis,” Nature Communications 15 (2024): 8628. 10.1038/s41467-024-53038-2 PMC11452667 39366998

[70] L. Hao , W. Zhong , X. Sun , and Z. Zhou , “TLR9 Signaling Protects Alcohol‐Induced Hepatic Oxidative Stress but Worsens Liver Inflammation in Mice,” Frontiers in Pharmacology 12 (2021): 709002. 10.3389/fphar.2021.709002 PMC8273378 34262465

[71] C. Galanos , M. A. Freudenberg , and W. Reutter , “Galactosamine‐Induced Sensitization to the Lethal Effects of Endotoxin,” Proceedings of the National Academy of Sciences of the United States of America 76 (1979): 5939–5943. 10.1073/pnas.76.11.5939 PMC411768 293694

