# Supplementary material for: Lactate‐Primed NETosis Modulates Hepatic Regeneration During Acute Liver Failure via the TLR9/KLF15/AJUBA Axis
Source: Cell Prolif. 2026 Jun 22;59(11):e70251. doi: 10.1111/cpr.70251 (PMC13325889; doi:10.1111/cpr.70251)
Supplement: Supplementary file 2 [file CPR-59-e70251-s005.docx]

**Legends of Supplementary Files**

**Fig. S1.** **Validation of neutrophil purity and metabolic profiling of ALF scRNA-seq data.** (A) Chemical structure of Cy3-labeled lactate. (B) Flow cytometry analysis illustrating the percentage of Ly6g^+^CD11b^+^ cells in the isolated neutrophil population (n=3). (C) UMAP plot visualization and dot plot showing the expression of representative marker genes used for the identification of distinct cell subclusters. (D) UMAP plots demonstrating the dynamic shift in glycolysis across cell clusters between the Control and ALF groups. (E) UMAP plots illustrating the heterogeneity of the TCA cycle across cell clusters in the Control and ALF groups. (F) UMAP plots comparing the signature of glycolysis between the KC_glycolysis_pos and KC_glycolysis_neg clusters within the Control and ALF groups.

**Fig. S2.** **Annotation of PHx scRNA-seq data and effects of AJUBA OE on hepatocyte proliferation.** (A) UMAP plot visualization and dot plot displaying the expression levels of representative marker genes used for cell cluster identification in the Control and PHx groups. (B) WB analysis of KLF15, AJUBA, YAP1, p-YAP1, and MKI67 protein levels in HepG2 cells from the CMV and AJUBA OE groups (n=6). (C) qRT-PCR analysis of *KLF15*, *AJUBA*, *YAP1*, and *MKI67* mRNA levels in HepG2 cells from the CMV and AJUBA OE groups (n=3). (D) EdU incorporation assay in HepG2 cells from the CMV and AJUBA OE groups (n=9). Scale bar = 50 μm. (E) Wound healing assay in HepG2 cells from the CMV, KLF15 OE, LKO, and AJUBA Sh groups (n=6). Scale bar = 100 μm. (F) Wound healing assay in AML12 cells from the CMV, Klf15 OE, LKO, and Ajuba Sh groups (n=6). Scale bar = 100 μm. (G) qRT-PCR analysis of *TLR9*, *RELA*, and *MYD88* mRNA levels in HepG2 cells from the CMV, CMV + LPS/D-Gal, CMV + NETs, CMV + LPS/D-Gal + NETs, AJUBA OE, AJUBA OE + LPS/D-Gal, AJUBA OE + NETs, and AJUBA OE + LPS/D-Gal + NETs groups (n=3). (H) qRT-PCR analysis of *KLF15*, *AJUBA*, *YAP1*, and *MKI6*7 mRNA levels in HepG2 cells from the CMV, CMV + LPS/D-Gal, CMV + NETs, CMV + LPS/D-Gal + NETs, AJUBA OE, AJUBA OE + LPS/D-Gal, AJUBA OE + NETs, and AJUBA OE + LPS/D-Gal + NETs groups (n=3).

**Fig. S3. Transcriptomic analysis of clinical liver tissues validates the expression changes of key markers during hepatic failure.** (A) Volcano plot (left) and heatmap (right) illustrating DEGs identified from transcriptome data of liver tissues from control and APAP-induced ALF patients. (B) Volcano plot (left) and heatmap (right) illustrating DEGs identified from transcriptome data of liver tissues from control and HBV-related ALF patients. (C) Volcano plot (left) and heatmap (right) illustrating DEGs identified from transcriptome data of liver tissues from control and HBV-related ACLF patients. (D) Heatmap depicting the expression profiles of markers in liver tissues from the ALF and ACLF patients.

**Table S1. The information on the used antibodies.**

**Table S2. Primer sequences for qRT-PCR.**
