# Supplementary material for: Lactate‐Primed NETosis Modulates Hepatic Regeneration During Acute Liver Failure via the TLR9/KLF15/AJUBA Axis
Source: Cell Prolif. 2026 Jun 22:e70251. Online ahead of print. doi: 10.1111/cpr.70251 (PMC13325889; doi:10.1111/cpr.70251)

Repeat 1

Figure 1

D

Cit-H3 (15 kDa)

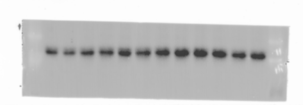

MCT1 (54 kDa)

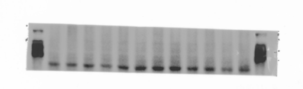

MCT1 (67 kDa)

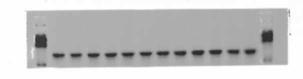

β-ACTIN (42 kDa)

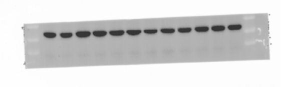

β-ACTIN (42 kDa)

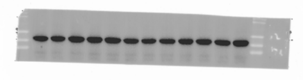

Figure 1

E

Cit-H3 (15 kDa)

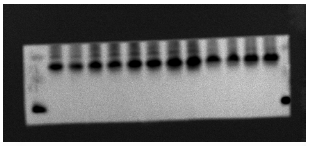

MCT1 (54 kDa)

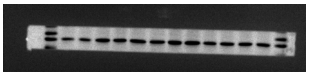

MCT1 (67 kDa)

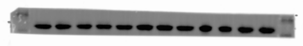

β-ACTIN (42 kDa)

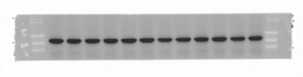

β-ACTIN (42 kDa)

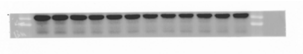

Figure 1

F

Cit-H3 (15 kDa)

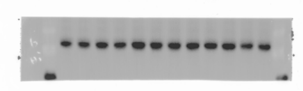

MCT1 (54 kDa)

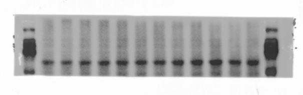

MCT1 (67 kDa)

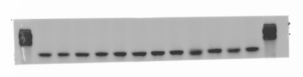

β-ACTIN (42 kDa)

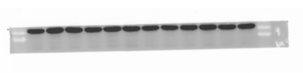

β-ACTIN (42 kDa)

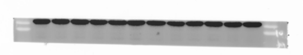

Figure 1

K

Cit-H3 (15 kDa)

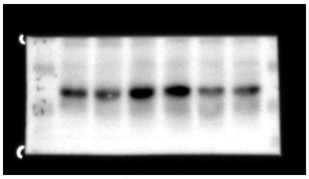

MCT1 (54 kDa)

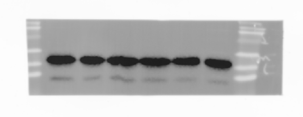

MCT1 (67 kDa)

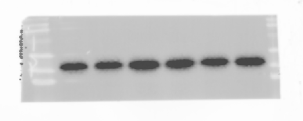

β-ACTIN (42 kDa)

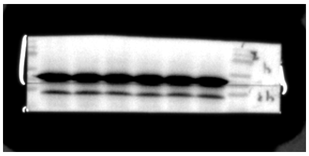

Figure 2

Q

LDHA (37 kDa)

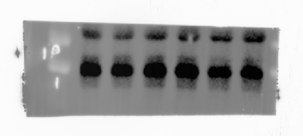

β-ACTIN (42 kDa)

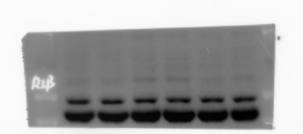

LDHB (35 kDa)

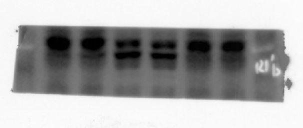

β-ACTIN (42 kDa)

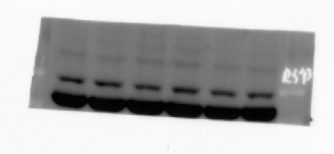

Figure 2

R

LDHA (37 kDa)

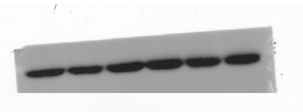

β-ACTIN (42 kDa)

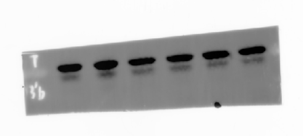

LDHB (35 kDa)

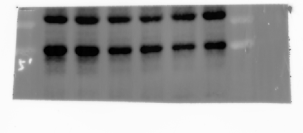

β-ACTIN (42 kDa)

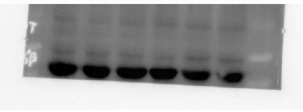

Figure 3

E

LDHA
(37 kDa)

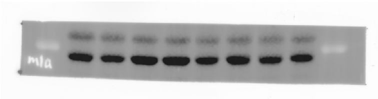

LDHB
(35 kDa)

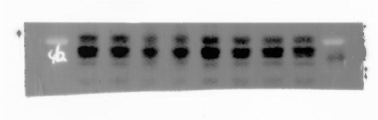

β-ACTIN
(42 kDa)

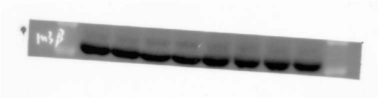

Cit-H3
(15 kDa)

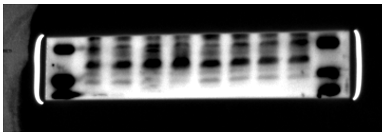

MCT1
(67 kDa)

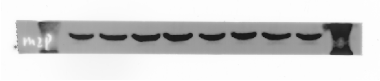

β-ACTIN
(42 kDa)

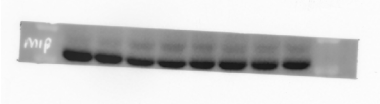

Figure 4

D

Cit-H3
(15 kDa)

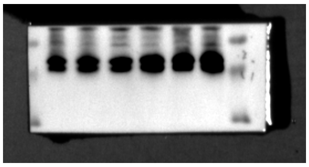

MCT1
(67 kDa)

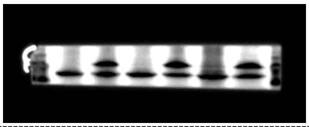

β-ACTIN
(42 kDa)

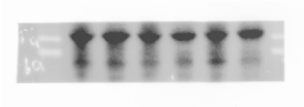

Figure 5

F

Kla

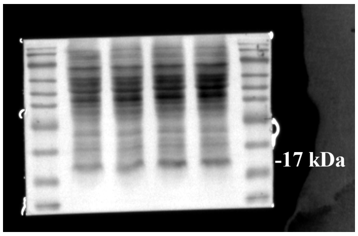

β-ACTIN
(42 kDa)

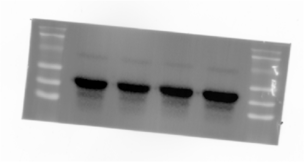

Histone H3
(17 kDa)

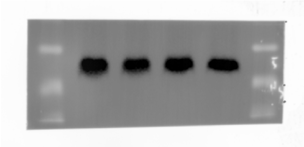

Figure 5

F

TLR9
(60 kDa)

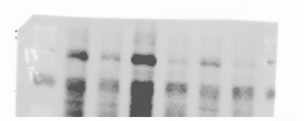

NFκB p65
(65 kDa)

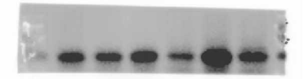

GAPDH
(35 kDa)

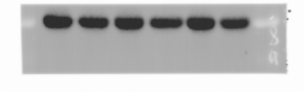

p- NFκB p65
(65 kDa)

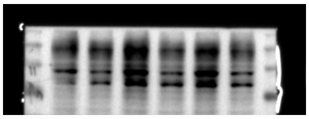

MYD88
(33 kDa)

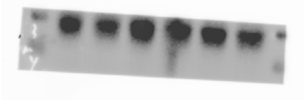

GAPDH
(35 kDa)

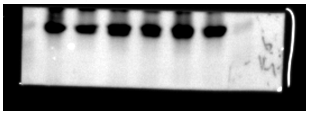

Figure 5

I

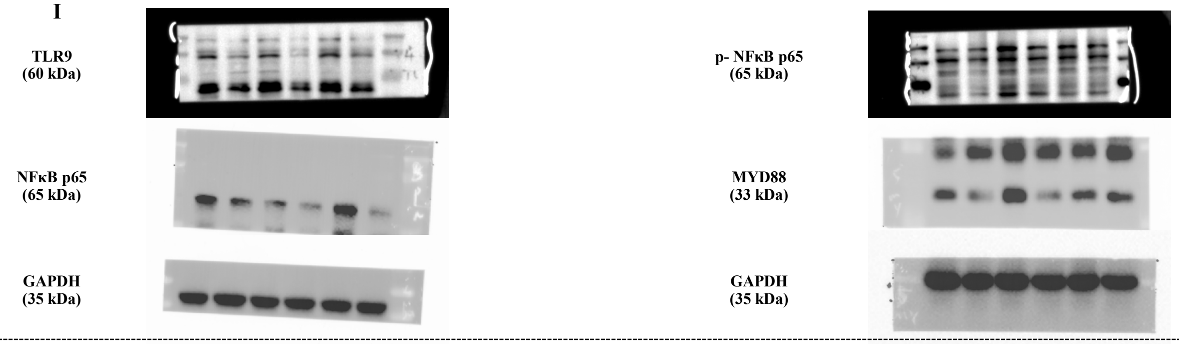

Figure 6

E-F

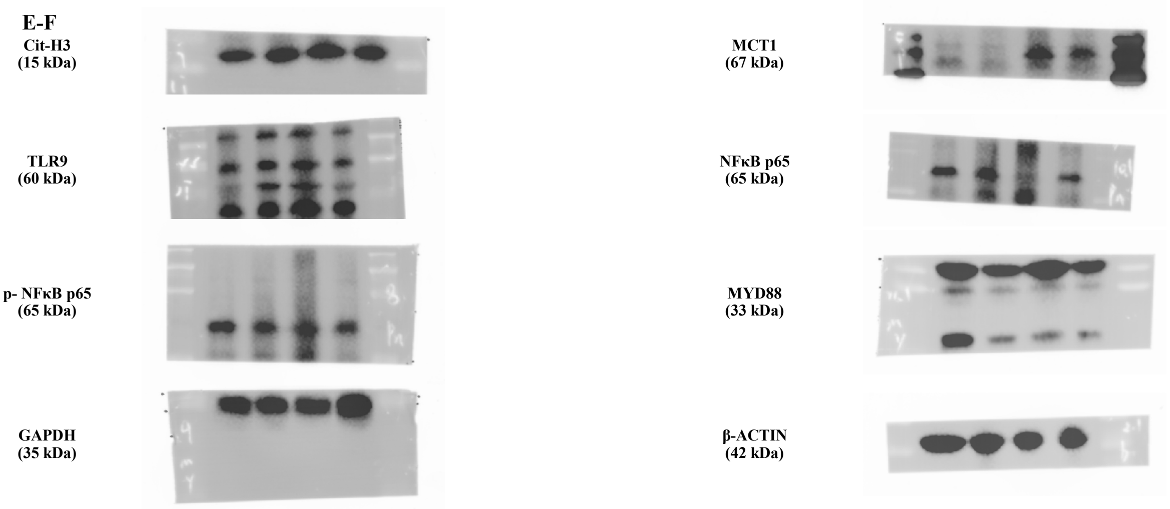

Figure 6

L-M

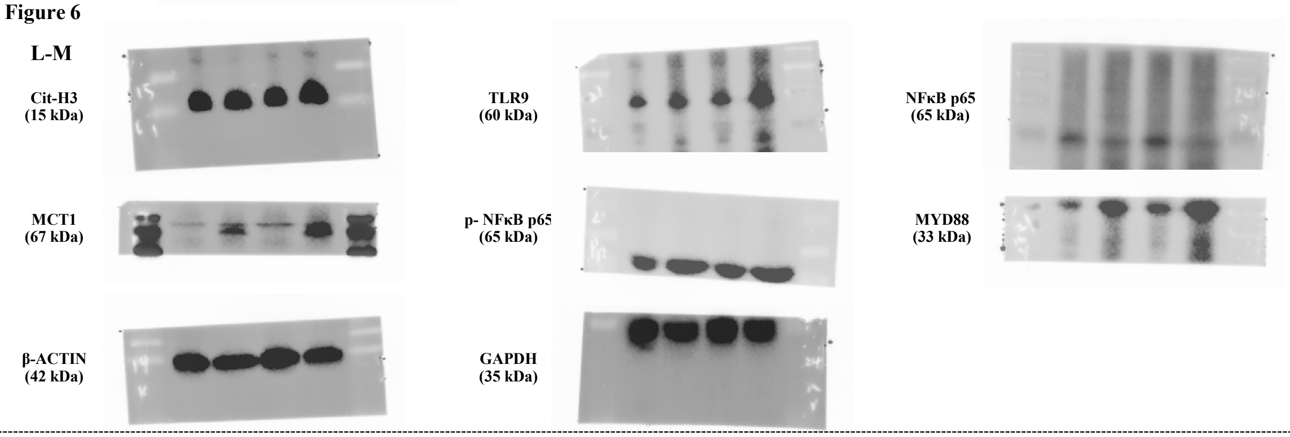

Figure 6

O

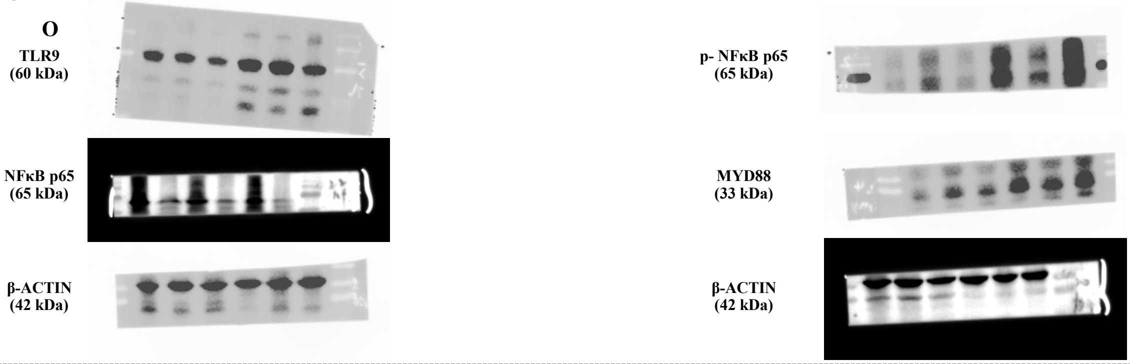

Figure 8

B

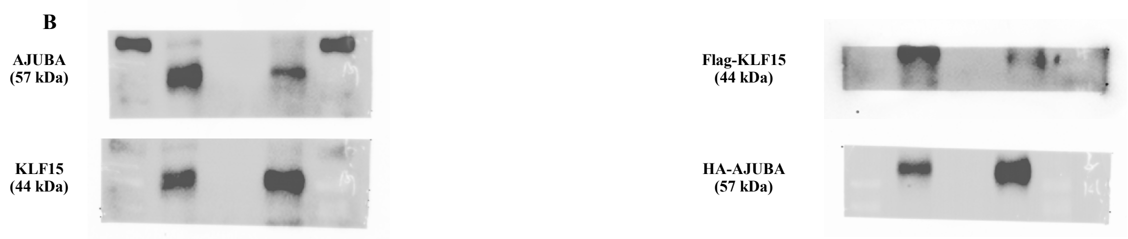

Figure 8

C

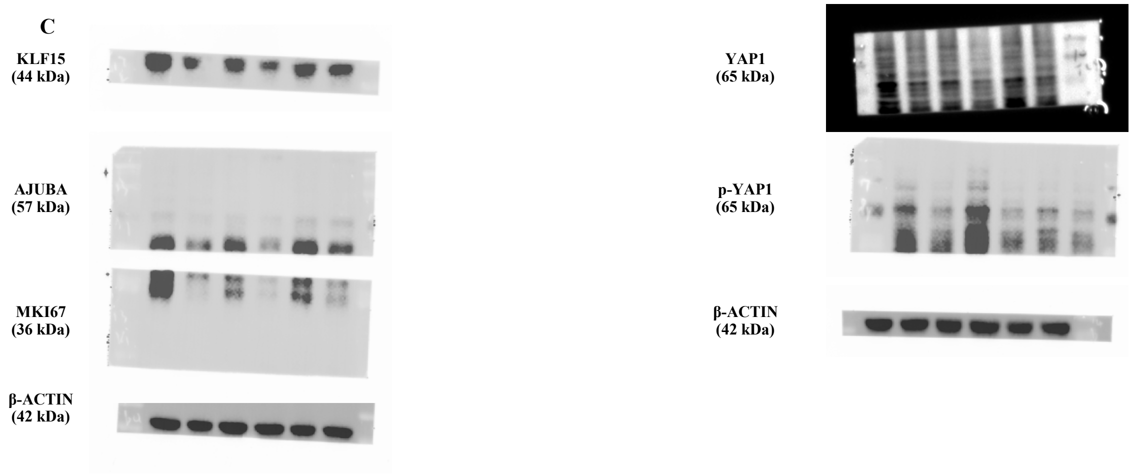

Figure 8

D

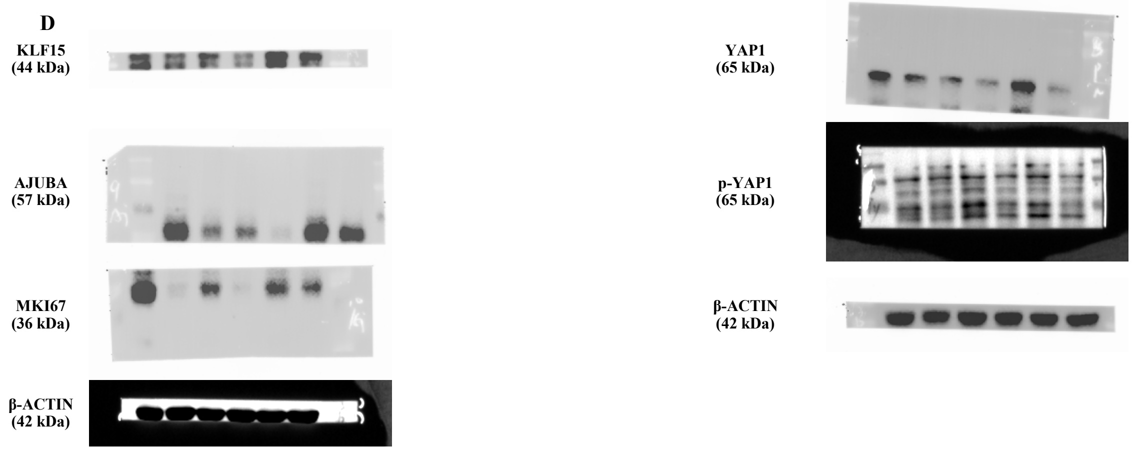

Figure 8

I

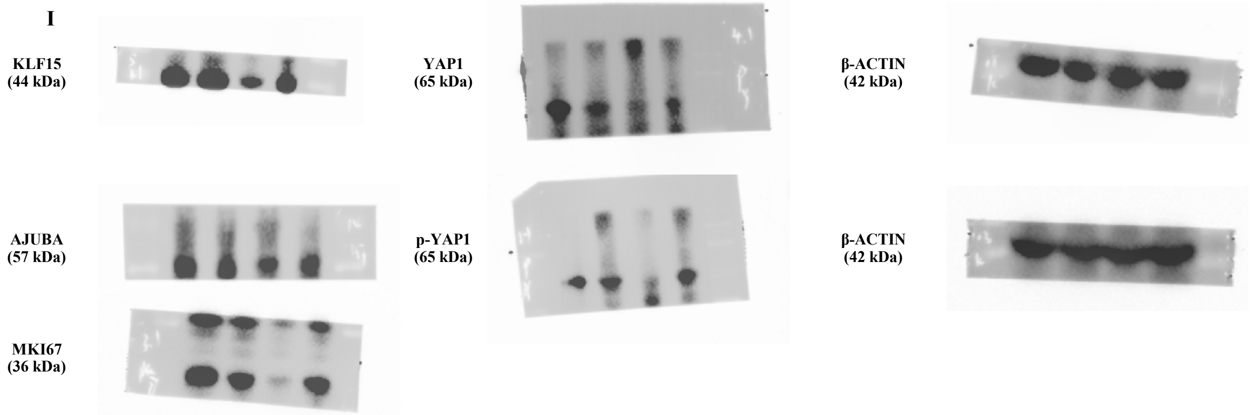

Figure 8

K

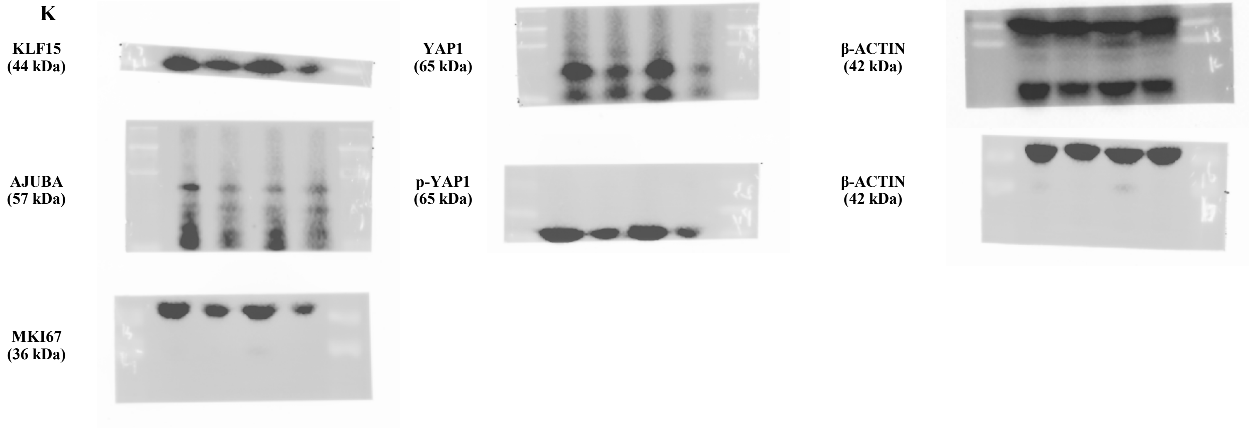

Figure 8

M

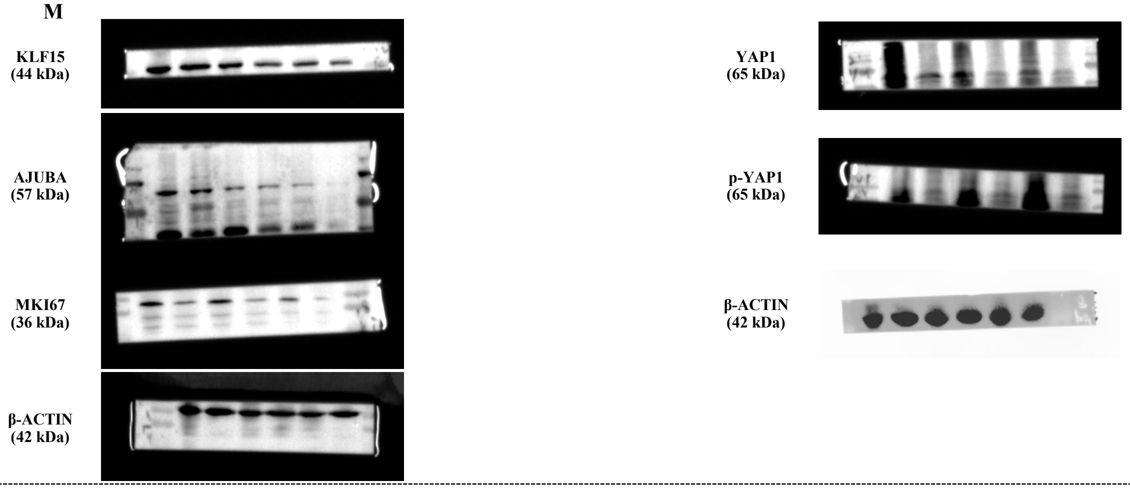

Figure 9

A

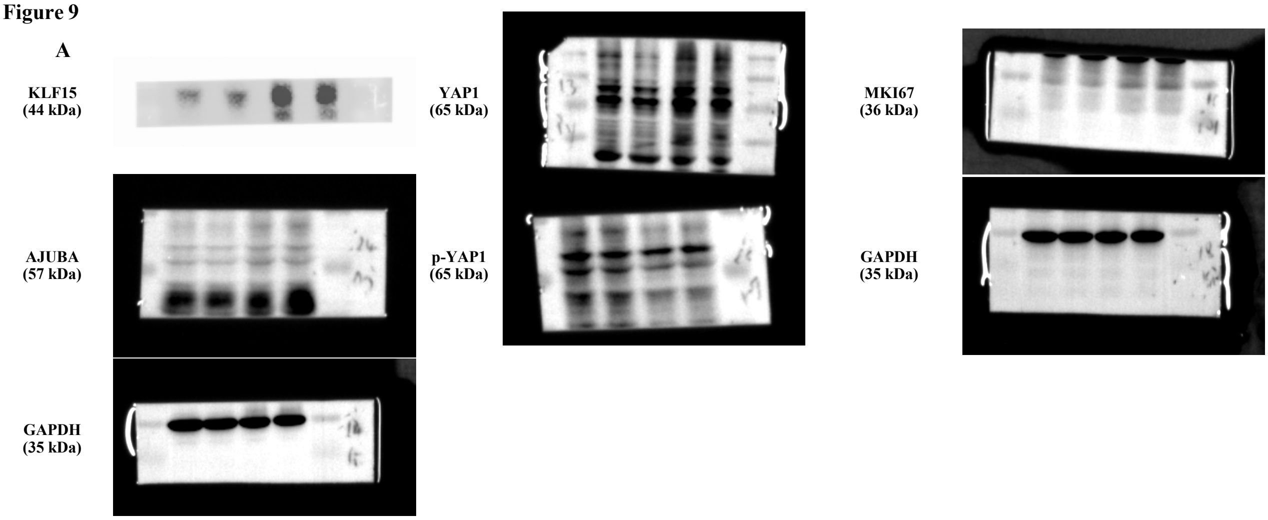

Figure 9

C

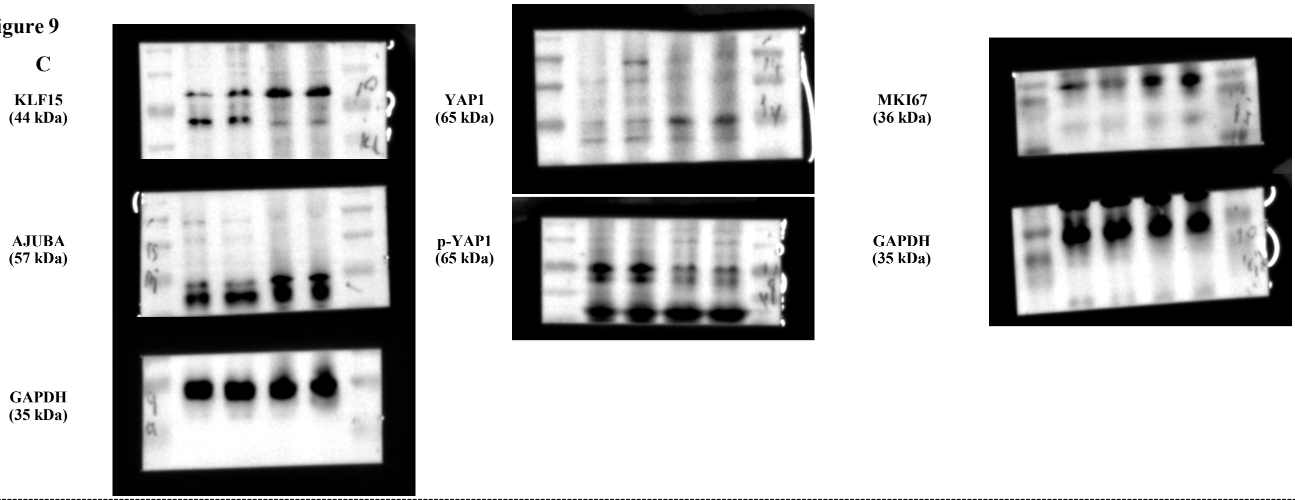

Figure 9

E

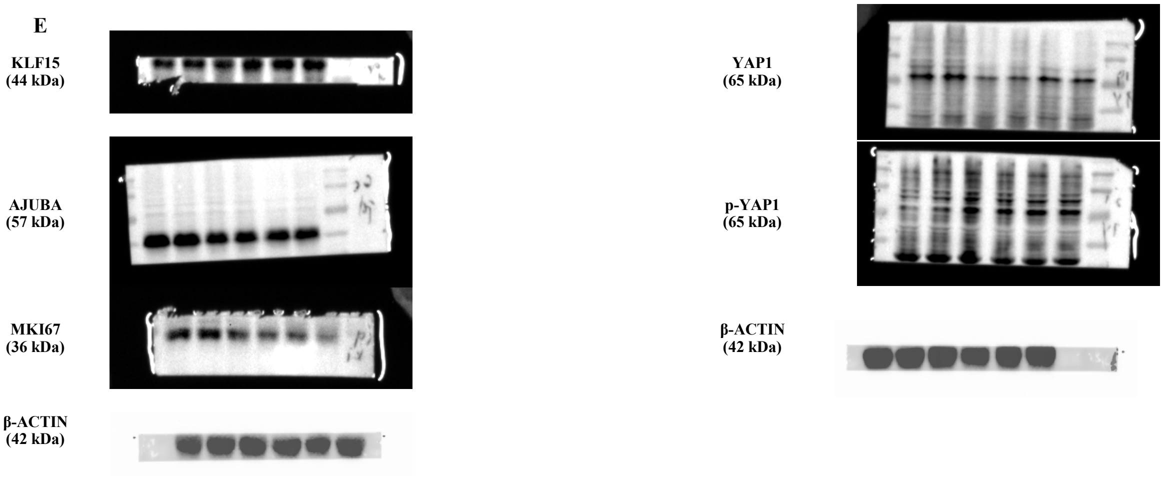

Figure 9

G

KLF15
(44 kDa)

AJUBA
(57 kDa)

MKI67
(36 kDa)

GAPDH
(35 kDa)

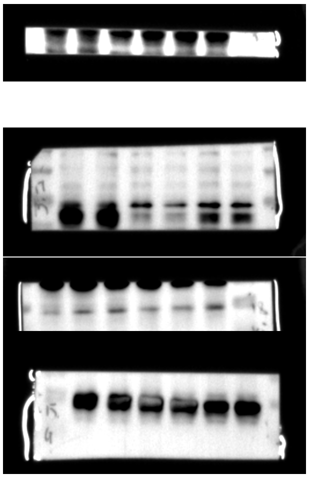

YAP1
(65 kDa)

p-YAP1
(65 kDa)

GAPDH
(35 kDa)

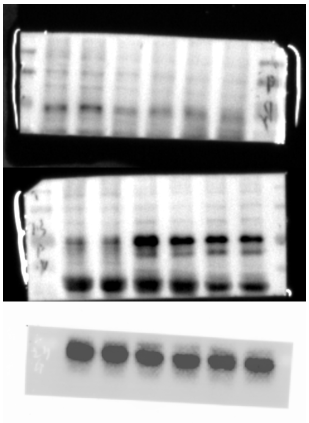

Figure 10

E

KLF15
(44 kDa)

AJUBA
(57 kDa)

MKI67
(36 kDa)

β-ACTIN
(42 kDa)

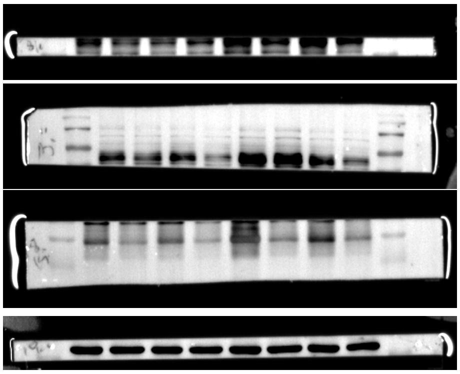

YAP1
(65 kDa)

p-YAP1
(65 kDa)

β-ACTIN
(42 kDa)

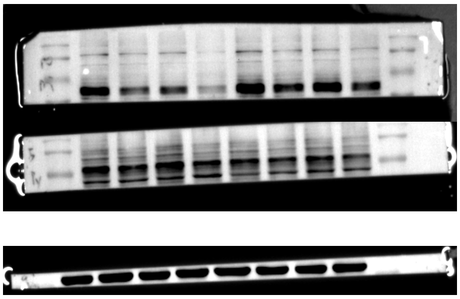

Figure 10

F

KLF15
(44 kDa)

AJUBA
(57 kDa)

MKI67
(36 kDa)

β-ACTIN
(42 kDa)

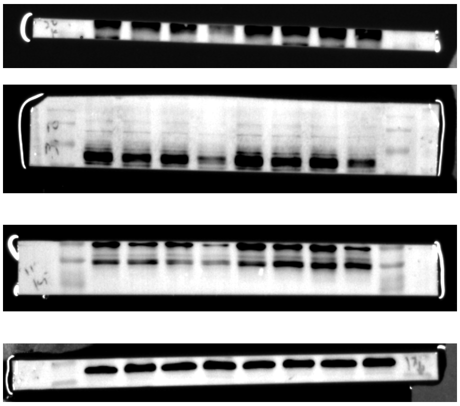

YAP1
(65 kDa)

p-YAP1
(65 kDa)

β-ACTIN
(42 kDa)

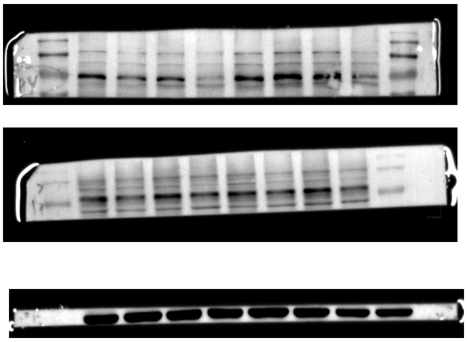

Figure S2

B

KLF15
(44 kDa)

AJUBA
(57 kDa)

GAPDH
(35 kDa)

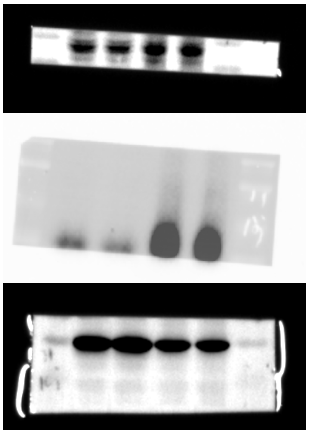

YAP1
(65 kDa)

p-YAP1
(65 kDa)

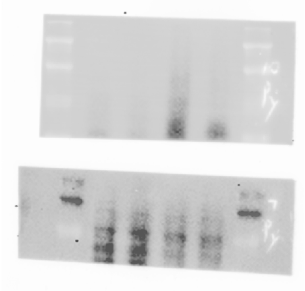

MKI67
(36 kDa)

GAPDH
(35 kDa)

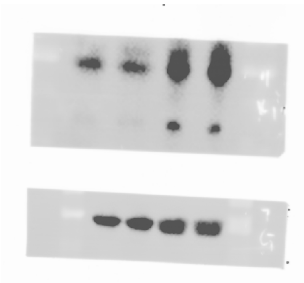

Repeat 2

Figure 1

D

Cit-H3 (15 kDa)

PAD4 (67 kDa)

β-ACTIN (42 kDa)

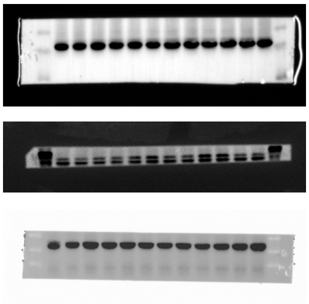

MCT1 (54 kDa)

β-ACTIN (42 kDa)

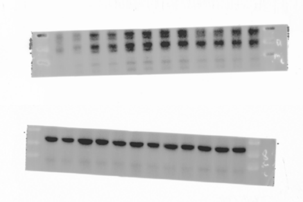

Figure 1

E

Cit-H3 (15 kDa)

PAD4 (67 kDa)

β-ACTIN (42 kDa)

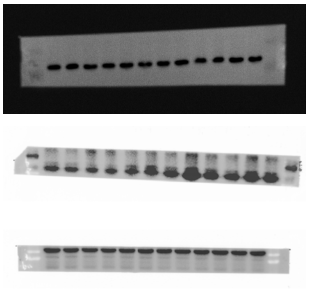

MCT1 (54 kDa)

β-ACTIN (42 kDa)

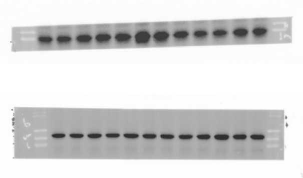

Figure 1

F

Cit-H3 (15 kDa)

PAD4 (67 kDa)

β-ACTIN (42 kDa)

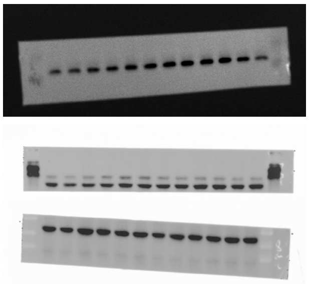

MCT1 (54 kDa)

β-ACTIN (42 kDa)

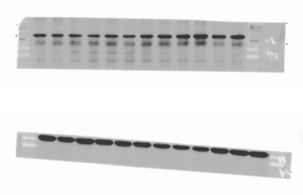

Figure 1

K

Cit-H3 (15 kDa)

PAD4 (67 kDa)

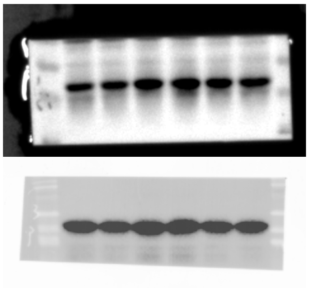

MCT1 (54 kDa)

β-ACTIN (42 kDa)

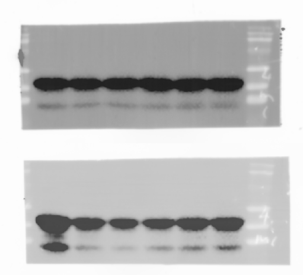

Figure 2

Q

LDHA (37 kDa)

LDHB (35 kDa)

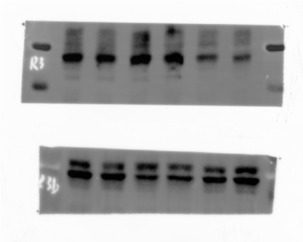

β-ACTIN (42 kDa)

β-ACTIN (42 kDa)

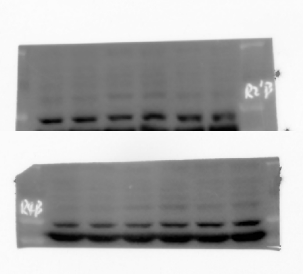

Figure 2

R

LDHA (37 kDa)

LDHB (35 kDa)

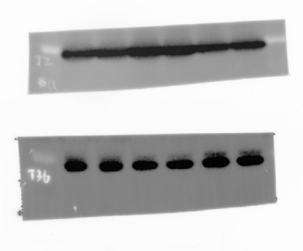

β-ACTIN (42 kDa)

β-ACTIN (42 kDa)

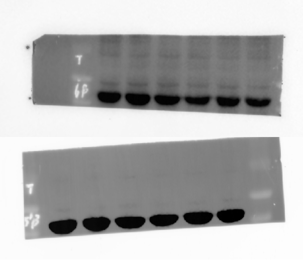

Figure 3

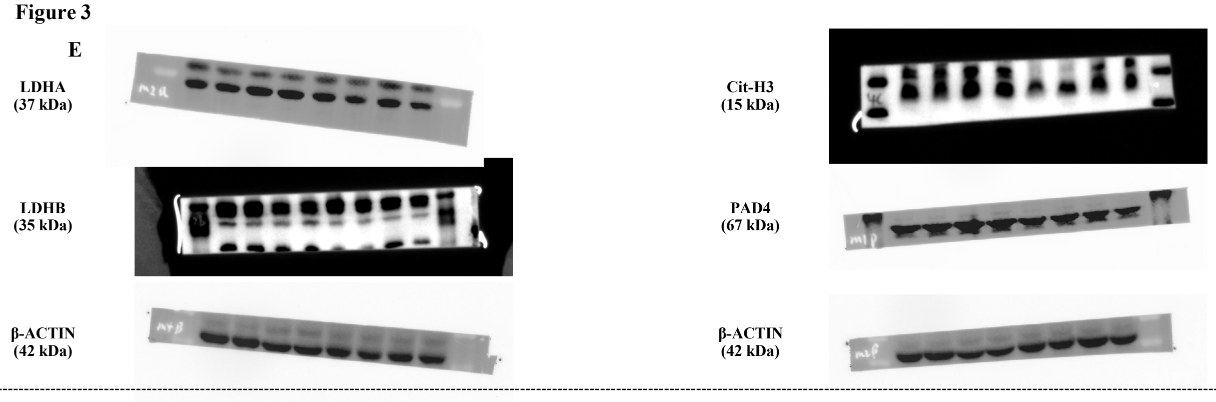

Figure 4

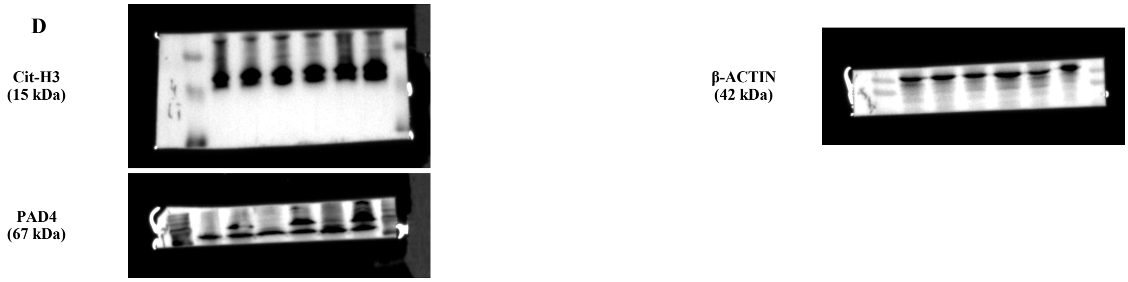

Figure 5

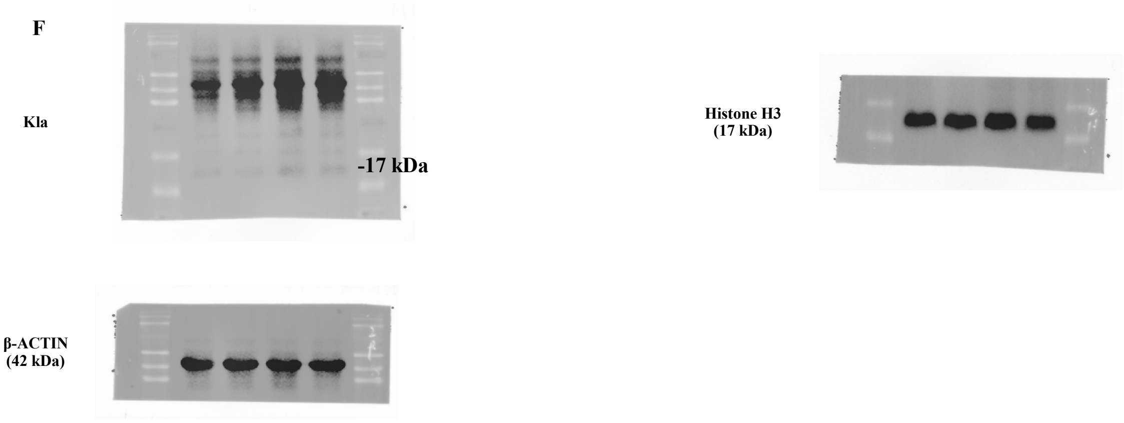

Figure 5

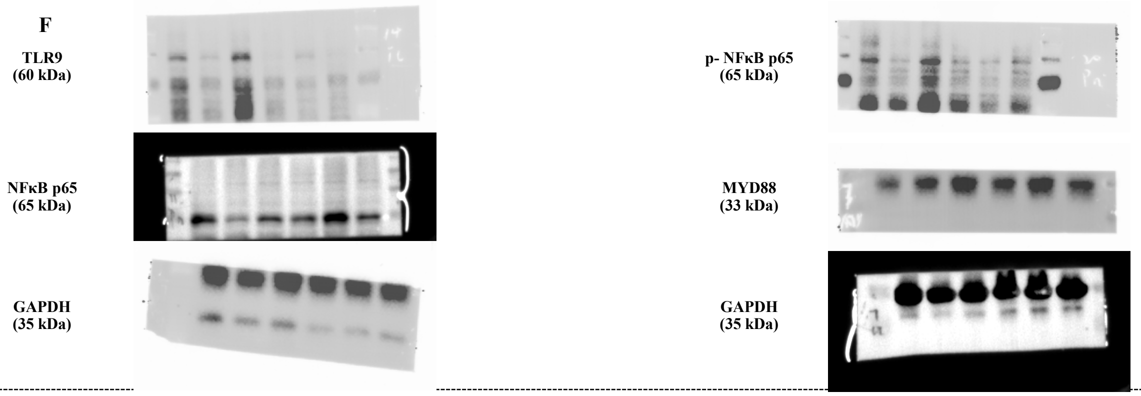

Figure 5

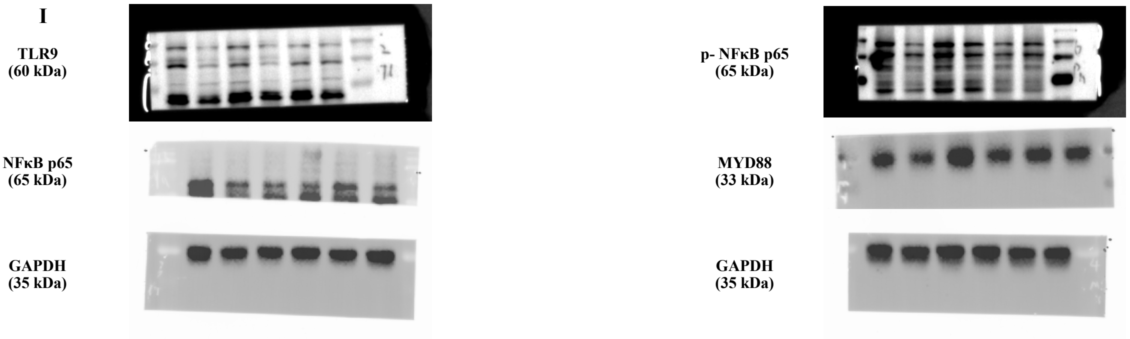

Figure 6

E-F

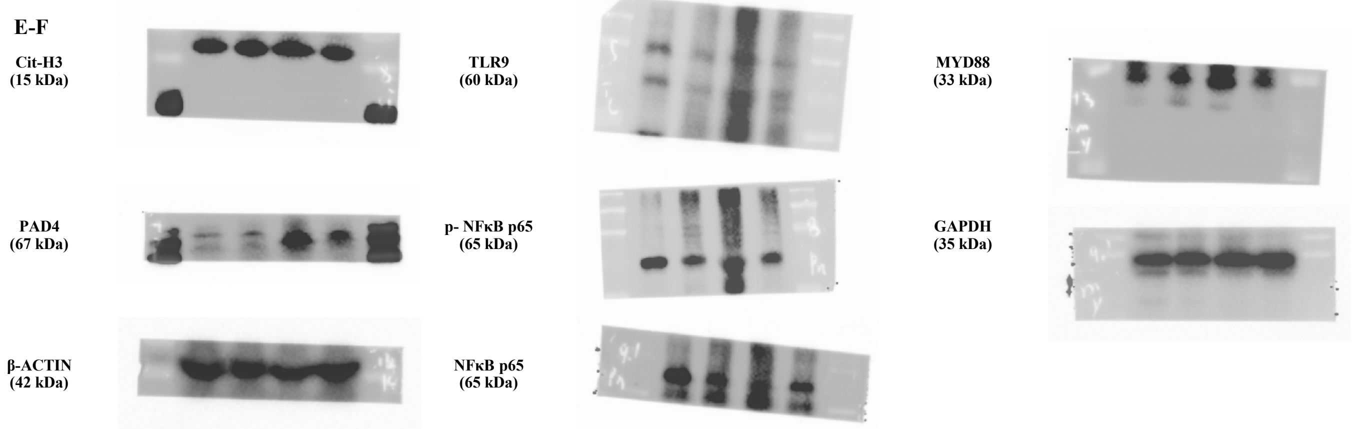

Figure 6

L-M

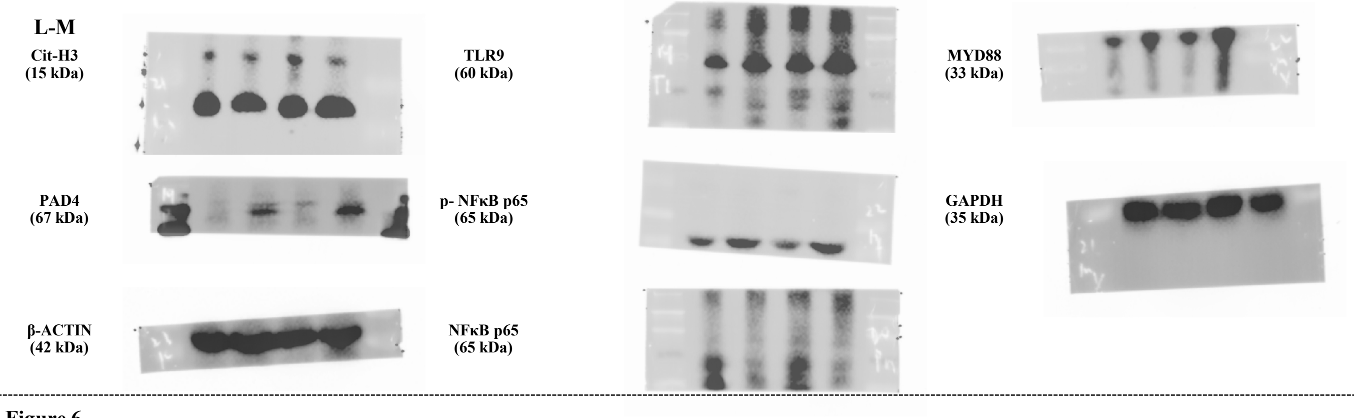

Figure 6

O

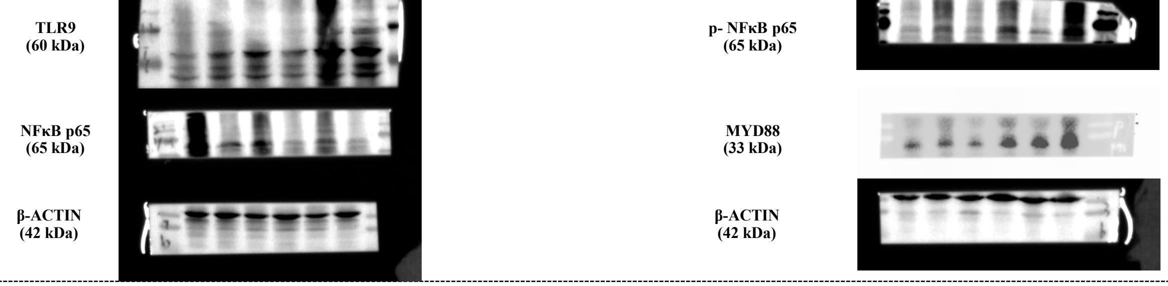

Figure 8

B

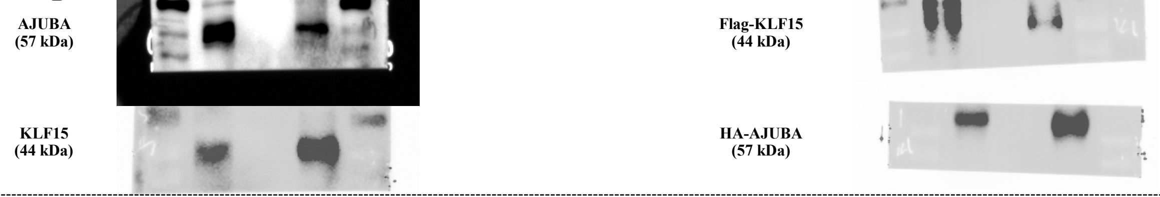

Figure 8

C

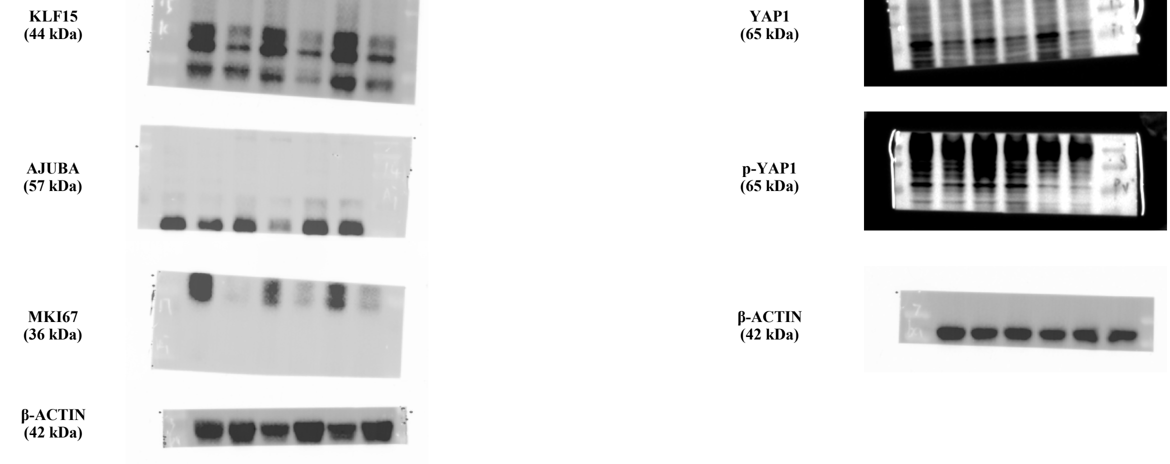

Figure 8

D

KLF15 (44 kDa)
AJUBA (57 kDa)
MKI67 (36 kDa)
β-ACTIN (42 kDa)

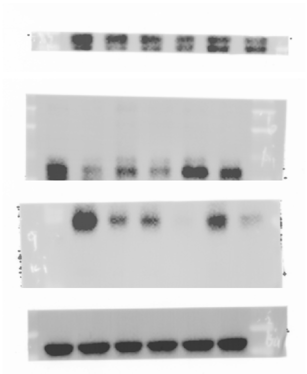

YAP1 (65 kDa)
p-YAP1 (65 kDa)
β-ACTIN (42 kDa)

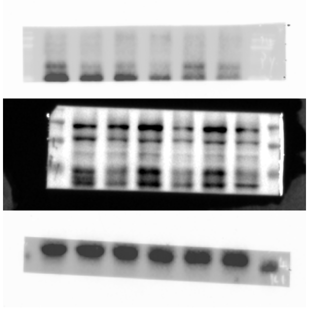

Figure 8

I

KLF15 (44 kDa)
AJUBA (57 kDa)
MKI67 (36 kDa)

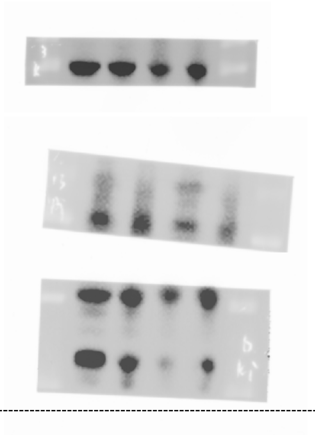

YAP1 (65 kDa)
p-YAP1 (65 kDa)

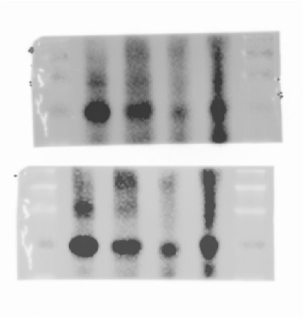

β-ACTIN (42 kDa)
β-ACTIN (42 kDa)

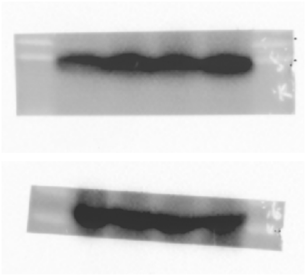

Figure 8

K

KLF15 (44 kDa)
AJUBA (57 kDa)
MKI67 (36 kDa)
β-ACTIN (42 kDa)

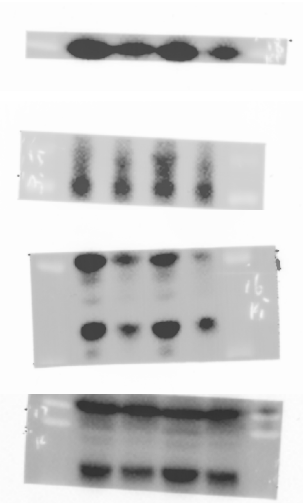

YAP1 (65 kDa)
p-YAP1 (65 kDa)
β-ACTIN (42 kDa)

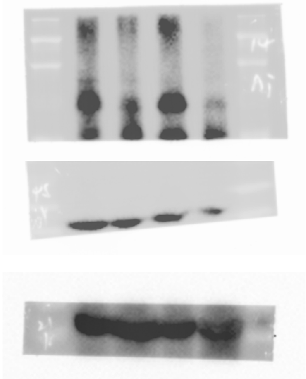

Figure 8

M

KLF15 (44 kDa)
AJUBA (57 kDa)
MKI67 (36 kDa)
β-ACTIN (42 kDa)

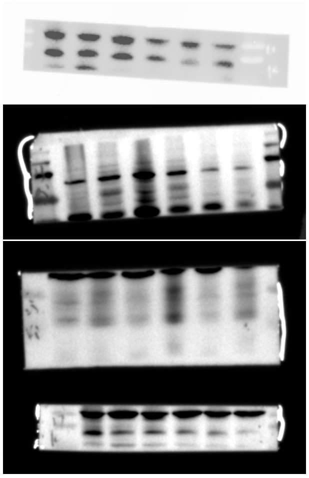

YAP1 (65 kDa)
p-YAP1 (65 kDa)
β-ACTIN (42 kDa)

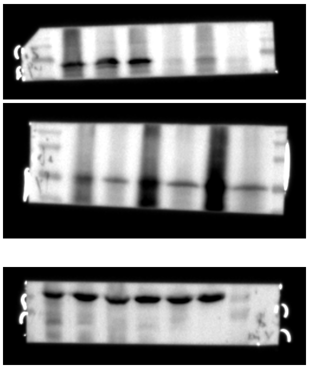

Figure 9

A

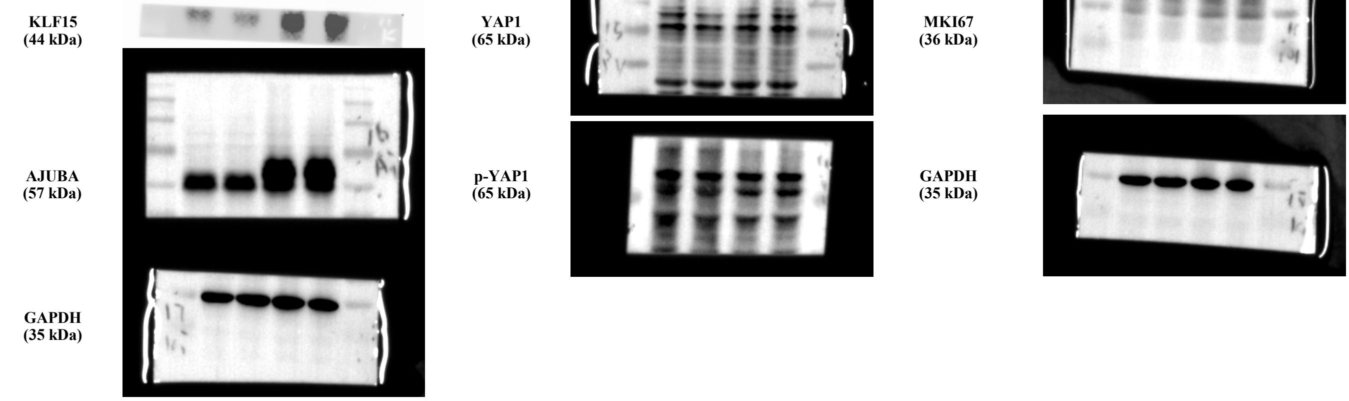

Figure 9

C

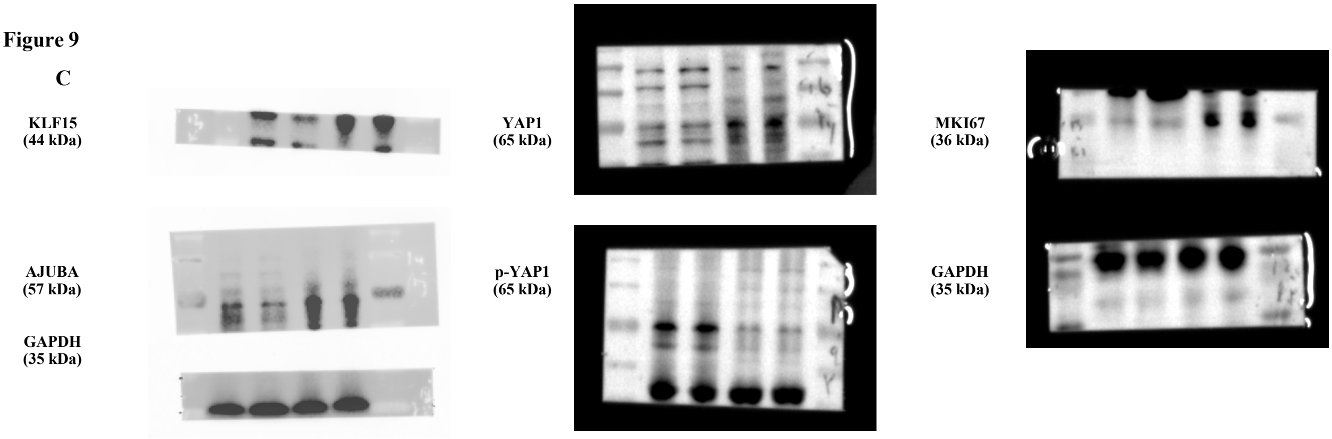

Figure 9

E

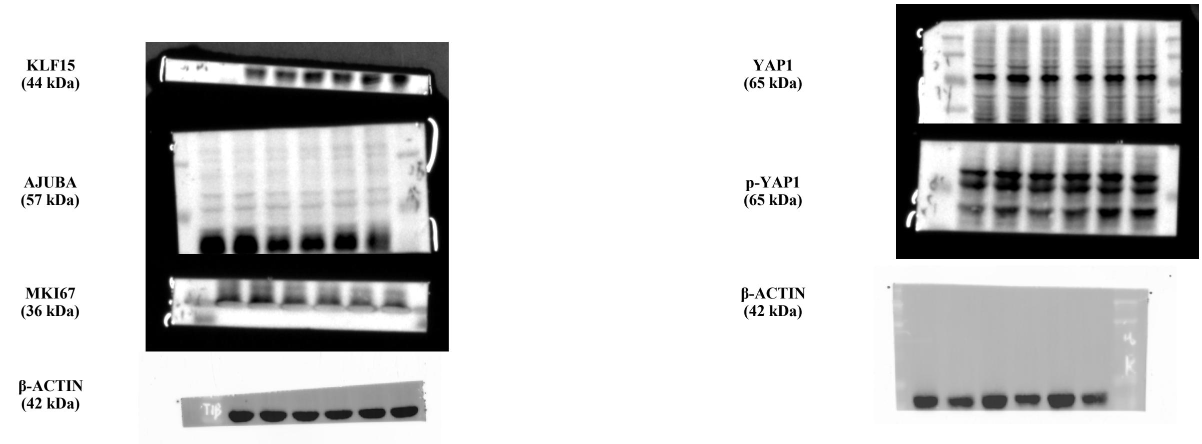

Figure 9

G

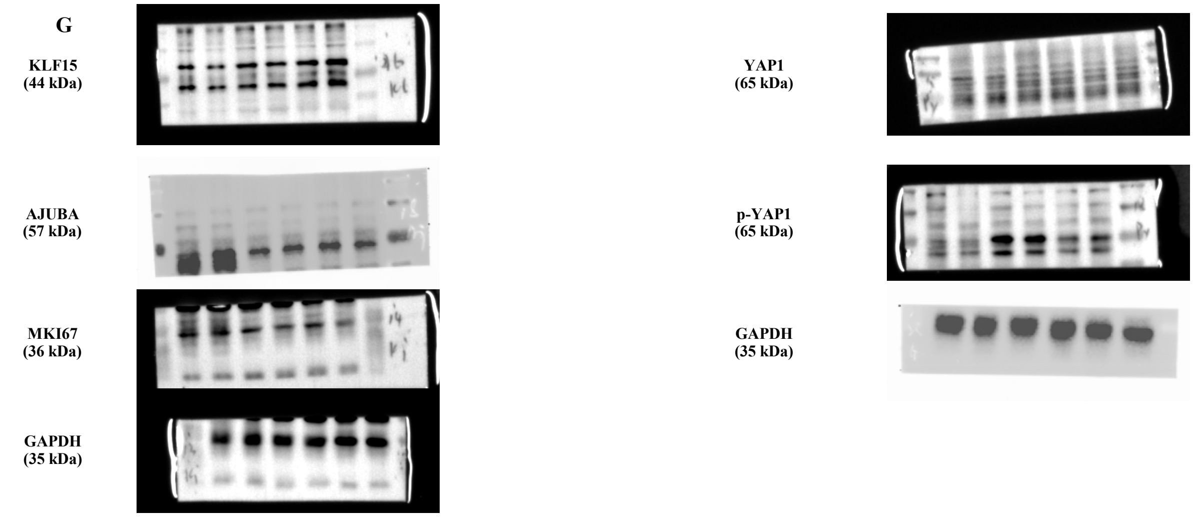

Figure 10

E

KLF15 (44 kDa)
AJUBA (57 kDa)
MKI67 (36 kDa)
β-ACTIN (42 kDa)

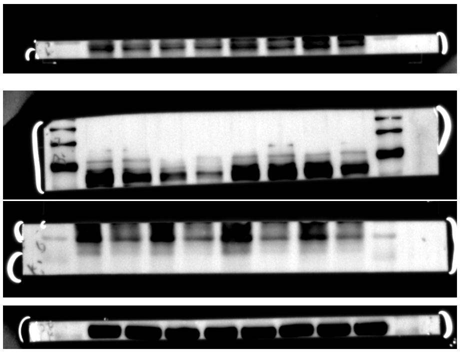

YAP1 (65 kDa)
p-YAP1 (65 kDa)
β-ACTIN (42 kDa)

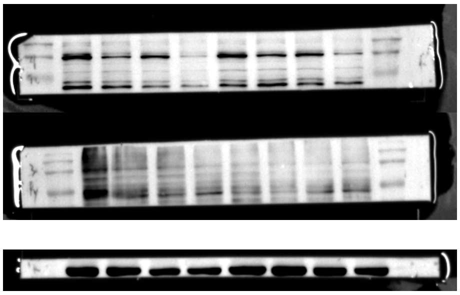

Figure 10

F

KLF15 (44 kDa)
AJUBA (57 kDa)
MKI67 (36 kDa)
β-ACTIN (42 kDa)

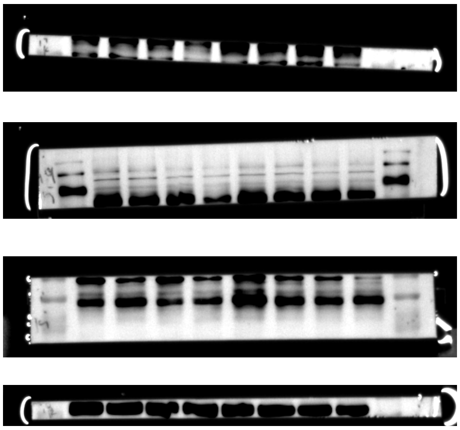

YAP1 (65 kDa)
p-YAP1 (65 kDa)
β-ACTIN (42 kDa)

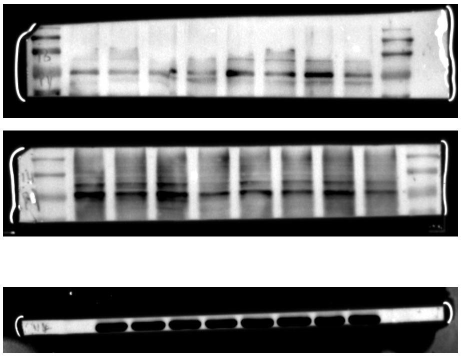

Figure S2

B

KLF15 (44 kDa)
AJUBA (57 kDa)
GAPDH (35 kDa)

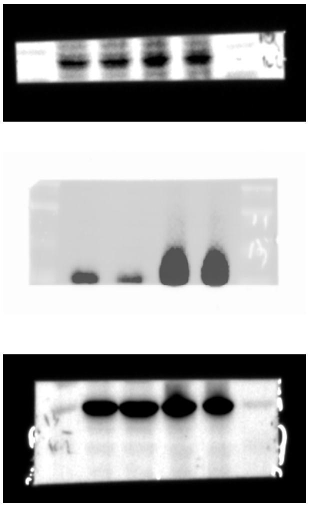

YAP1 (65 kDa)
p-YAP1 (65 kDa)

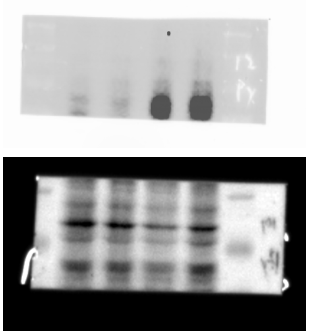

MKI67 (36 kDa)
GAPDH (35 kDa)

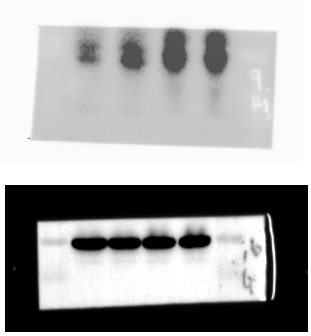

Repeat 3

Figure 1

D

Cit-H3
(15 kDa)

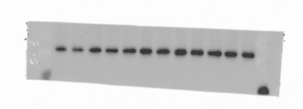

MCT1
(54 kDa)

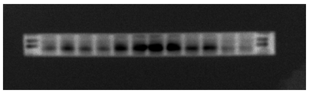

PAD4
(67 kDa)

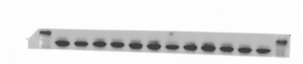

β-ACTIN
(42 kDa)

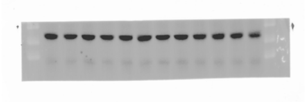

β-ACTIN
(42 kDa)

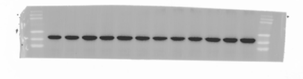

Figure 1

E

Cit-H3
(15 kDa)

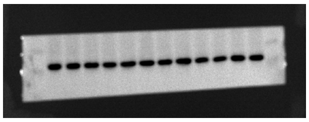

MCT1
(54 kDa)

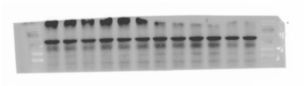

PAD4
(67 kDa)

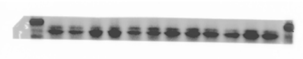

β-ACTIN
(42 kDa)

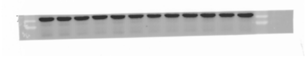

β-ACTIN
(42 kDa)

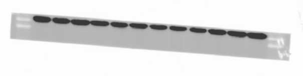

Figure 1

F

Cit-H3
(15 kDa)

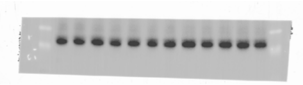

MCT1
(54 kDa)

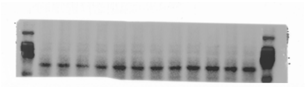

PAD4
(67 kDa)

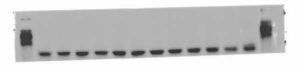

β-ACTIN
(42 kDa)

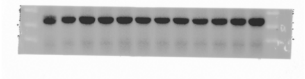

β-ACTIN
(42 kDa)

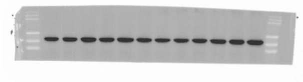

Figure 1

K

Cit-H3
(15 kDa)

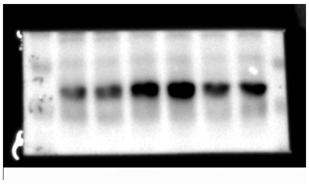

MCT1
(54 kDa)

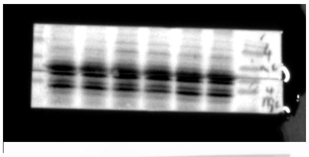

PAD4
(67 kDa)

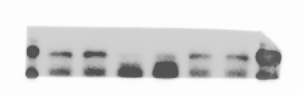

β-ACTIN
(42 kDa)

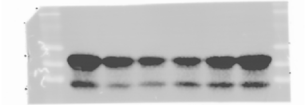

Figure 2

Q

LDHA
(37 kDa)

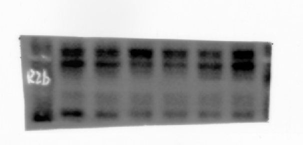

β-ACTIN
(42 kDa)

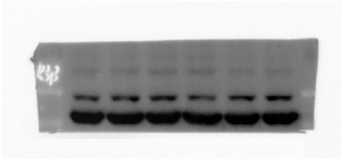

LDHB
(35 kDa)

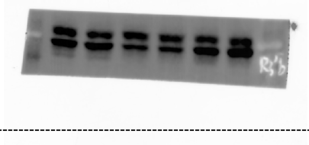

β-ACTIN
(42 kDa)

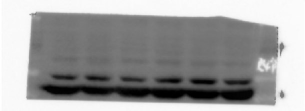

Figure 2

R

LDHA
(37 kDa)

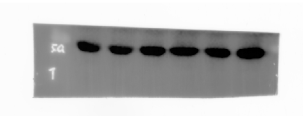

β-ACTIN
(42 kDa)

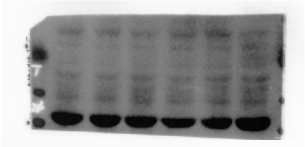

LDHB
(35 kDa)

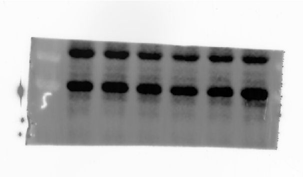

β-ACTIN
(42 kDa)

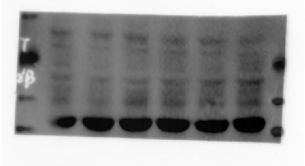

Figure 3

E

LDHA
(37 kDa)

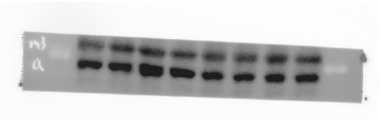

LDHB
(35 kDa)

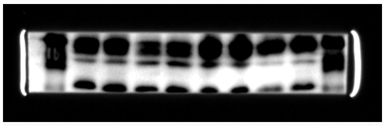

β-ACTIN
(42 kDa)

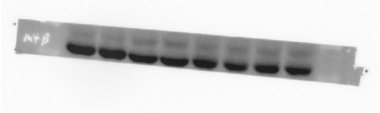

Cit-H3
(15 kDa)

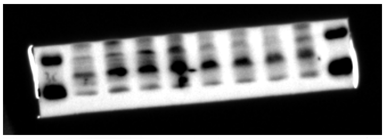

PAD4
(67 kDa)

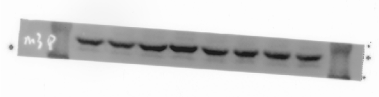

β-ACTIN
(42 kDa)

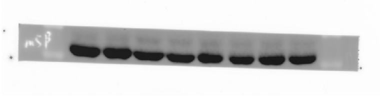

Figure 4

D

Cit-H3
(15 kDa)

PAD4
(67 kDa)

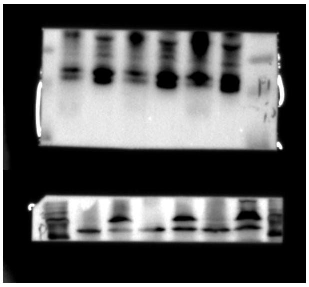

β-ACTIN
(42 kDa)

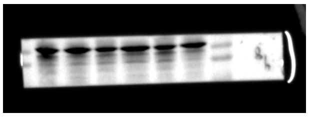

Figure 5

F

Kla

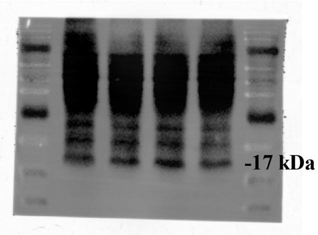

β-ACTIN
(42 kDa)

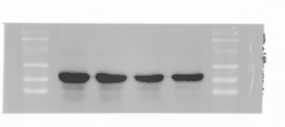

Histone H3
(17 kDa)

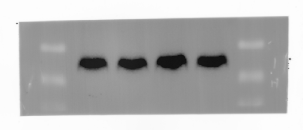

Figure 5

F

TLR9
(60 kDa)

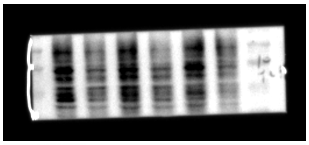

NFκB p65
(65 kDa)

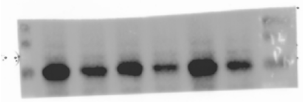

GAPDH
(35 kDa)

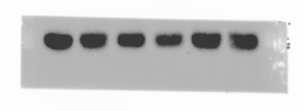

p- NFκB p65
(65 kDa)

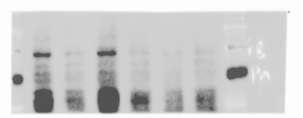

MYD88
(33 kDa)

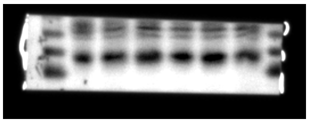

GAPDH
(35 kDa)

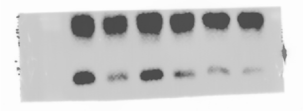

Figure 5

I

TLR9
(60 kDa)

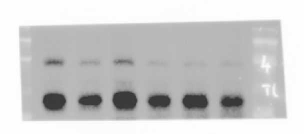

NFκB p65
(65 kDa)

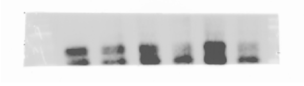

GAPDH
(35 kDa)

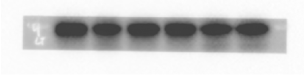

p- NFκB p65
(65 kDa)

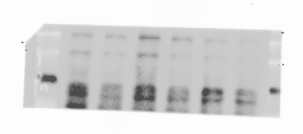

MYD88
(33 kDa)

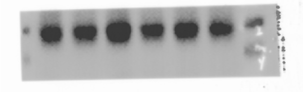

GAPDH
(35 kDa)

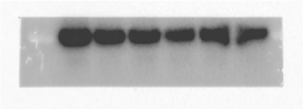

Figure 6

E-F

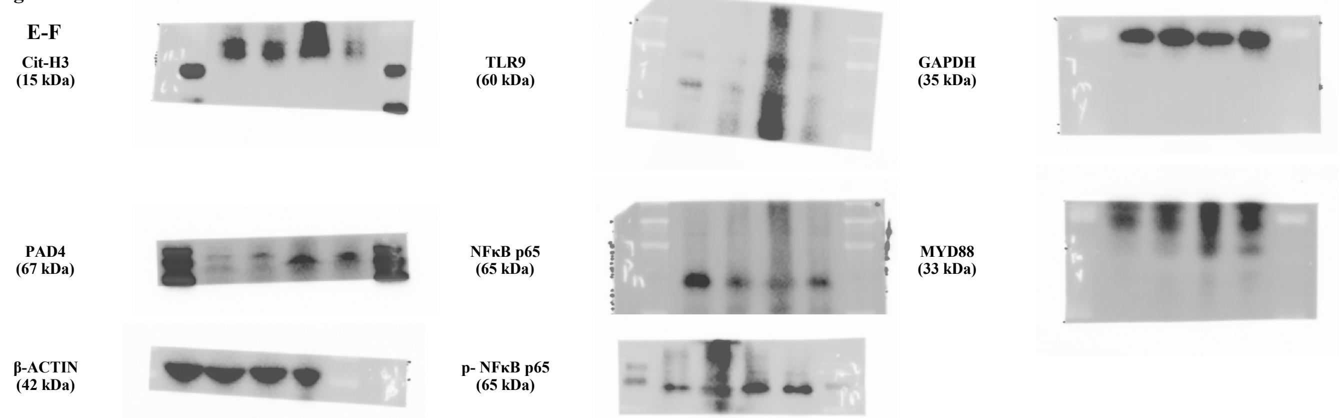

Figure 6

L-M

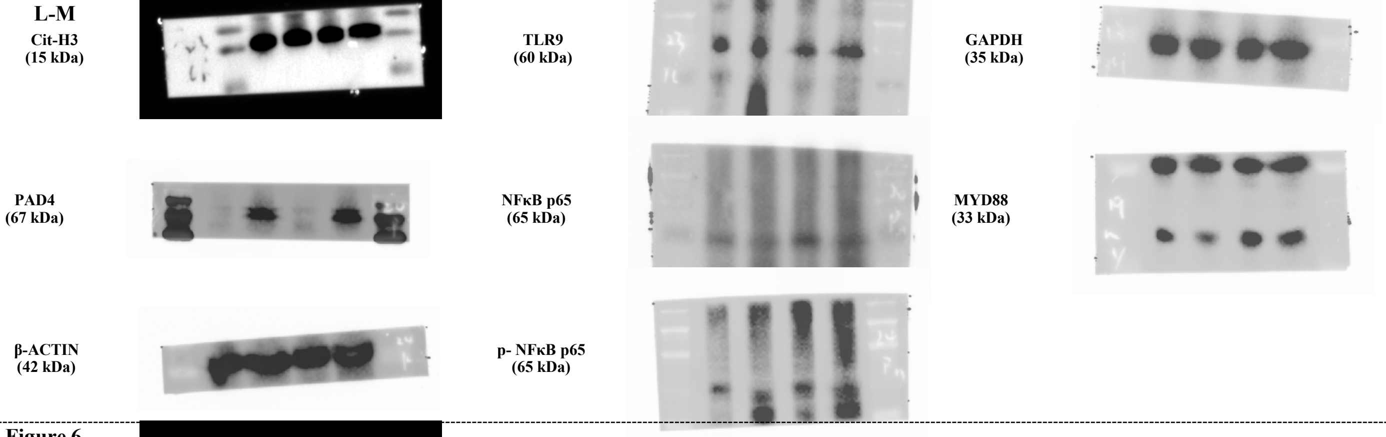

Figure 6

O

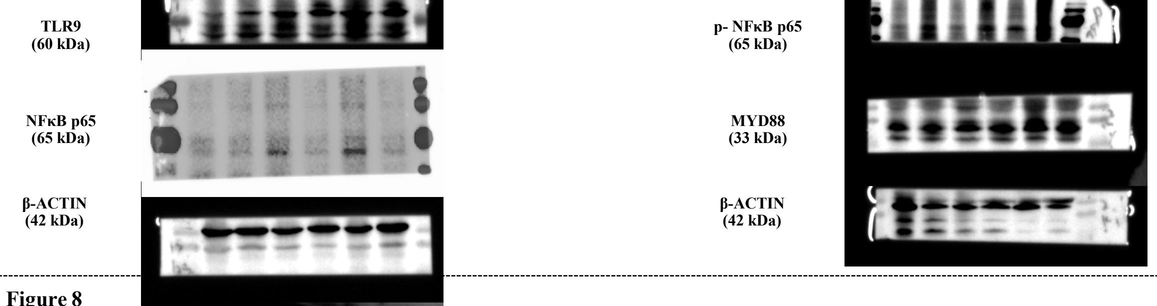

Figure 8

B

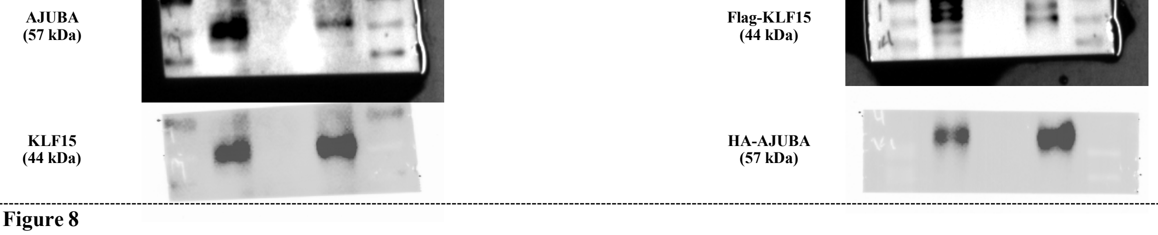

Figure 8

C

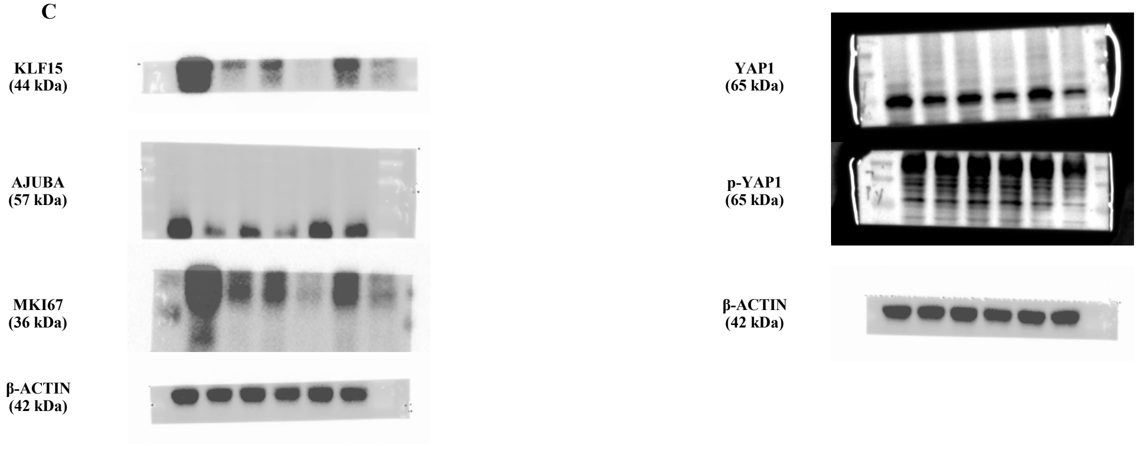

Figure 8

D

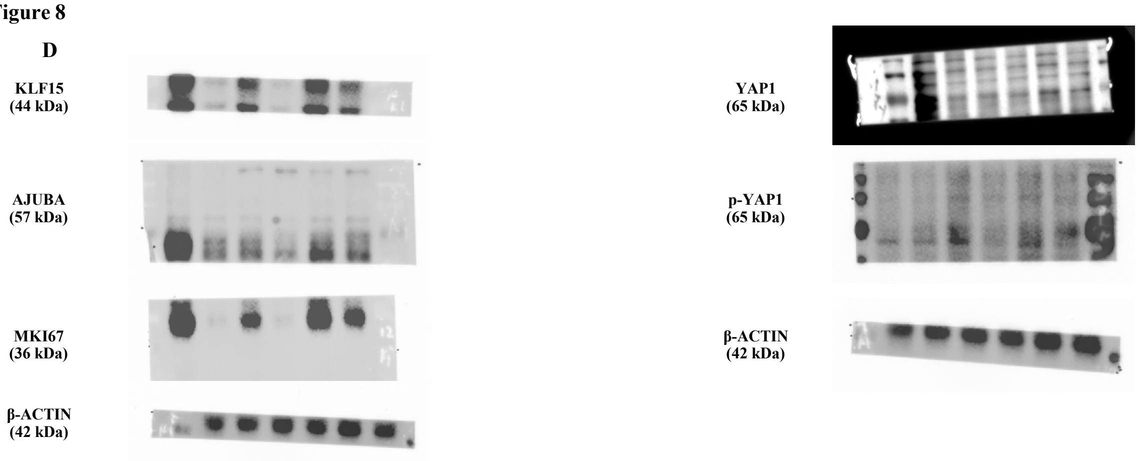

Figure 8

I

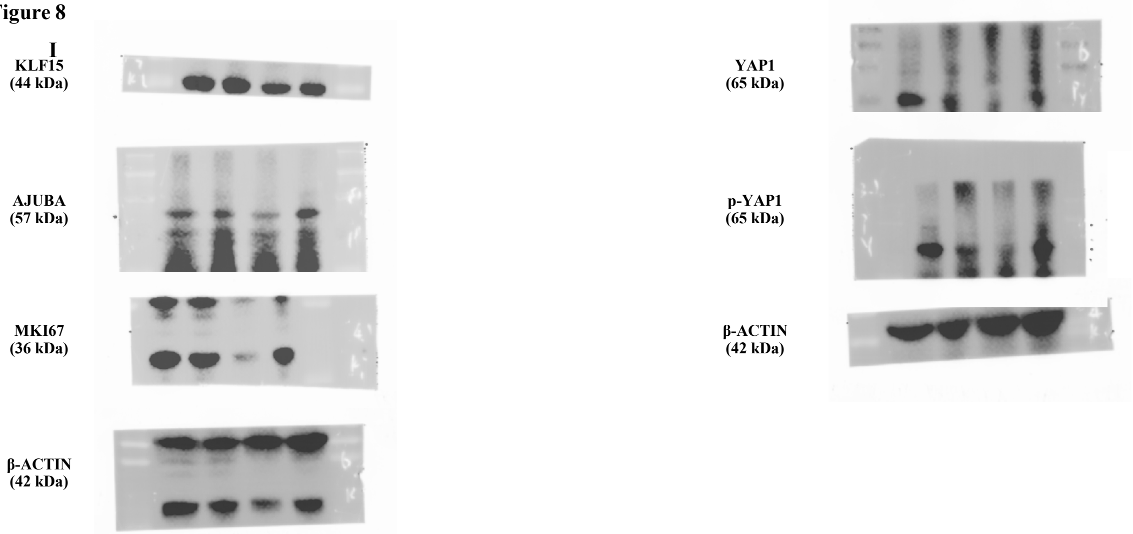

Figure 8

K

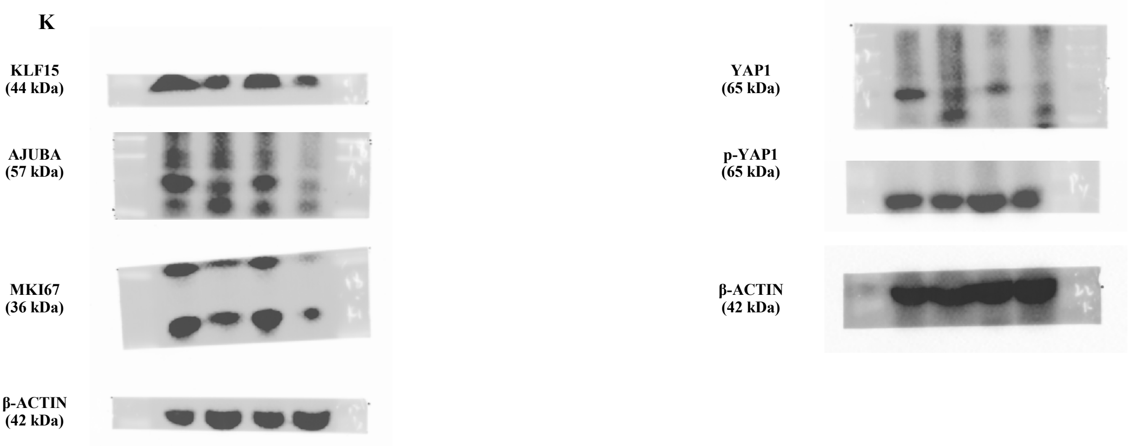

Figure 8

M

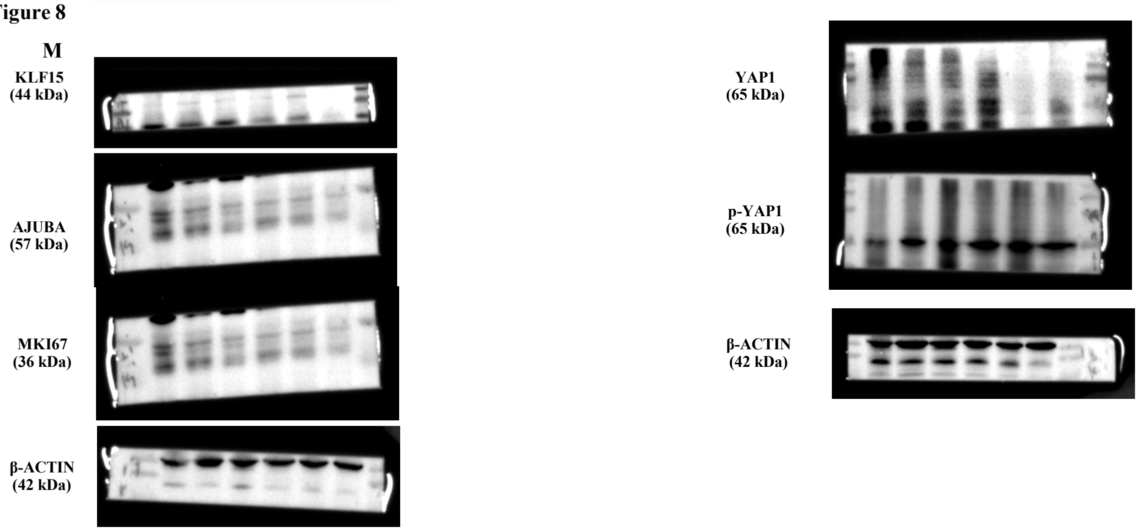

Figure 9

A

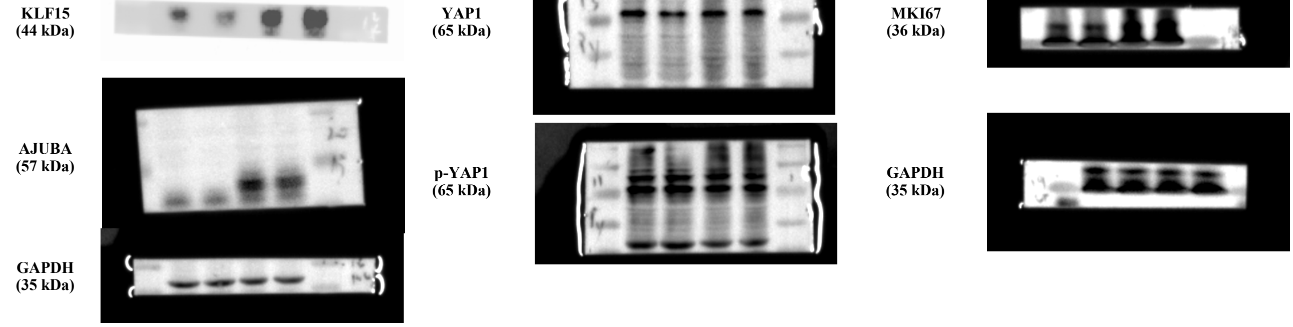

Figure 9

C

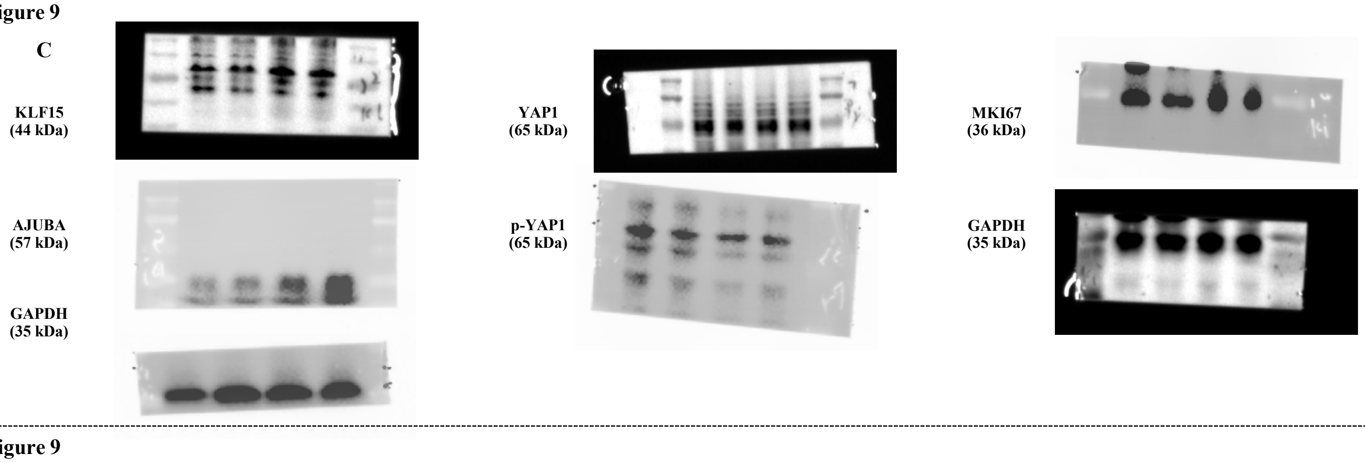

Figure 9

E

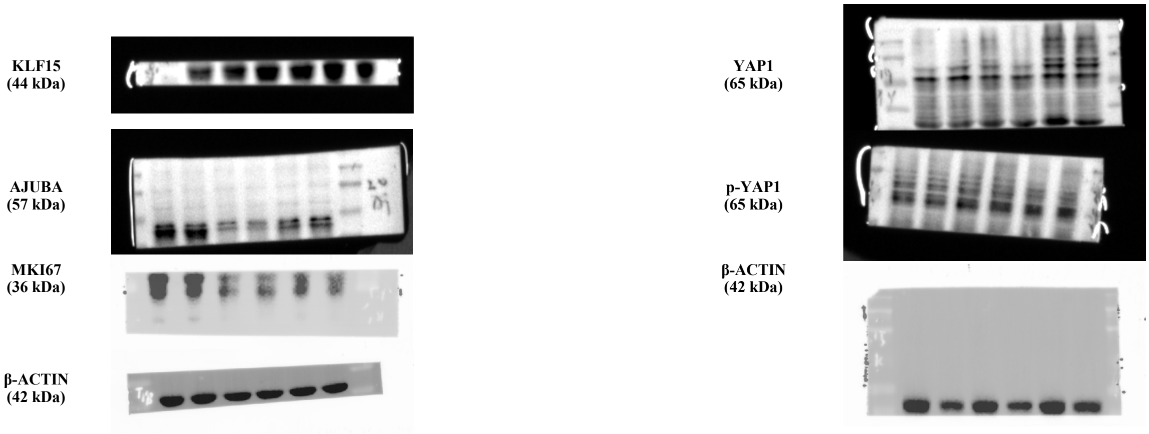

Figure 9

G

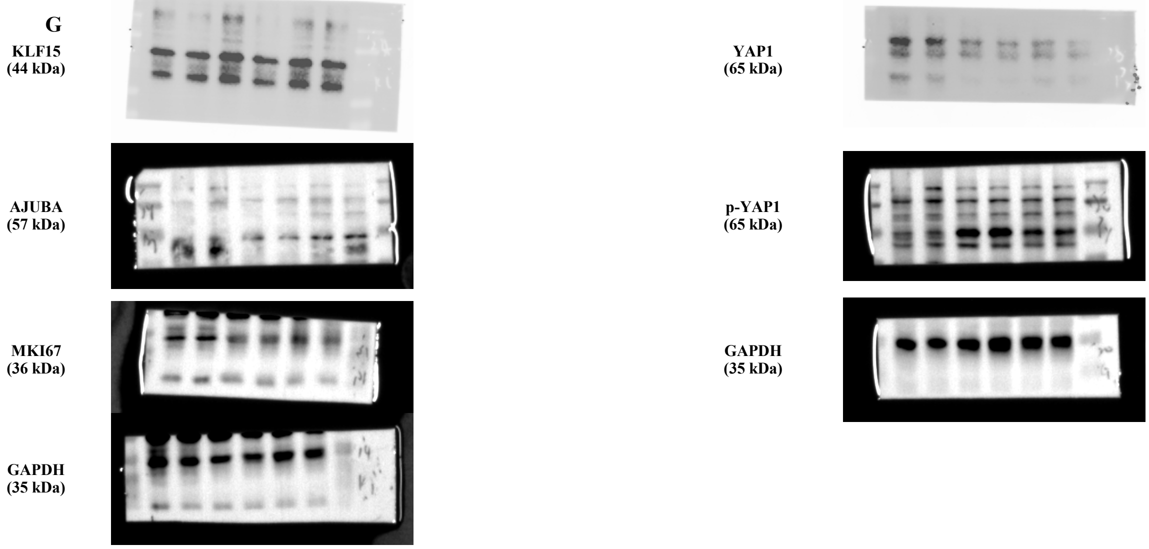

Figure 10

E

KLF15
(44 kDa)

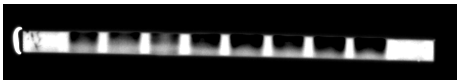

AJUBA
(57 kDa)

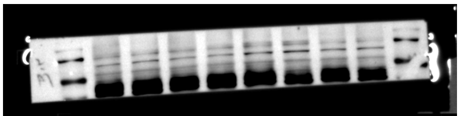

MKI67
(36 kDa)

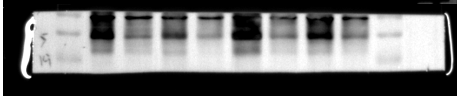

β-ACTIN
(42 kDa)

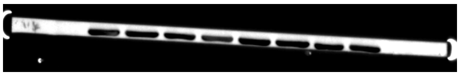

YAP1
(65 kDa)

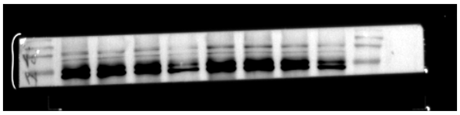

p-YAP1
(65 kDa)

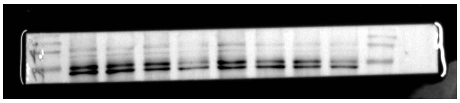

β-ACTIN
(42 kDa)

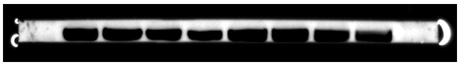

Figure 10

F

KLF15
(44 kDa)

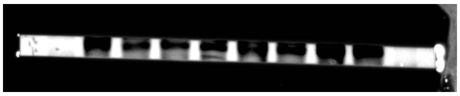

AJUBA
(57 kDa)

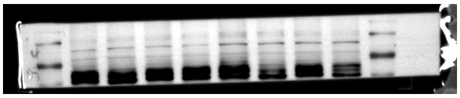

MKI67
(36 kDa)

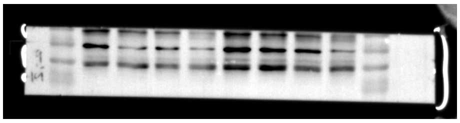

β-ACTIN
(42 kDa)

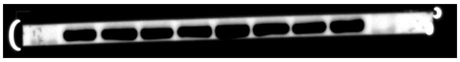

YAP1
(65 kDa)

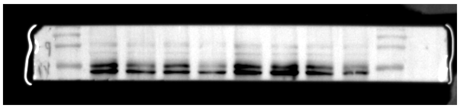

p-YAP1
(65 kDa)

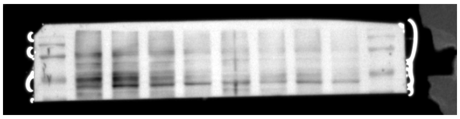

β-ACTIN
(42 kDa)

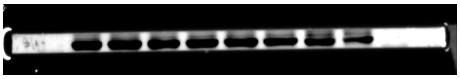

Figure S2

B

KLF15
(44 kDa)

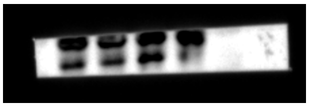

AJUBA
(57 kDa)

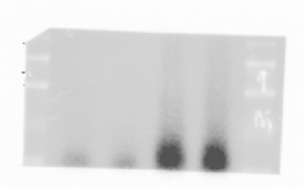

GAPDH
(35 kDa)

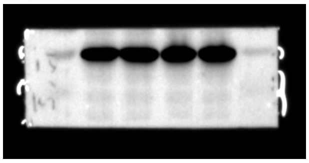

YAP1
(65 kDa)

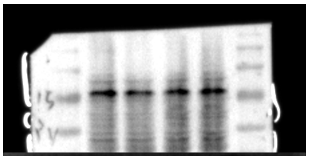

p-YAP1
(65 kDa)

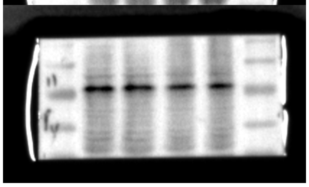

MKI67
(36 kDa)

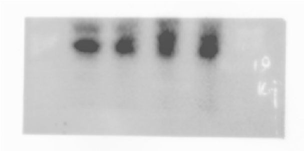

GAPDH
(35 kDa)

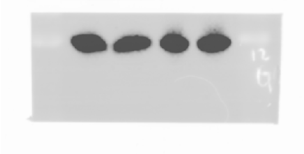

Supplement: Supplementary file 6 — Data S2: Origin western blots. [file CPR-9999-e70251-s003.pdf]
